# Adaptive insertion of a hydrophobic anchor into a poly(ethylene glycol) host for programmable surface functionalization

**DOI:** 10.1038/s41557-022-01090-0

**Published:** 2022-11-21

**Authors:** Shaohua Zhang, Wei Li, Jiabin Luan, Abhinav Srivastava, Vincenzo Carnevale, Michael L. Klein, Jiawei Sun, Danni Wang, Serena P. Teora, Sjoerd J. Rijpkema, Johannes D. Meeldijk, Daniela A. Wilson

**Affiliations:** 1 Institute for Molecules and Materials, Radboud University, Nijmegen, the Netherlands; 2 Institute for Genomics and Evolutionary Medicine (iGEM) and Department of Biology, Temple University, Philadelphia, PA USA; 3 Institute for Computational Molecular Science, Temple University, Philadelphia, PA USA; 4 Inorganic Chemistry and Catalysis, Debye Institute for Nanomaterials Science, Utrecht University, Utrecht, the Netherlands

**Keywords:** Self-assembly, Nanostructures, Supramolecular polymers

## Abstract

Covalent and non-covalent molecular binding are two strategies to tailor surface properties and functions. However, the lack of responsiveness and requirement for specific binding groups makes spatiotemporal control challenging. Here, we report the adaptive insertion of a hydrophobic anchor into a poly(ethylene glycol) (PEG) host as a non-covalent binding strategy for surface functionalization. By using polycyclic aromatic hydrocarbons as the hydrophobic anchor, hydrophilic charged and non-charged functional modules were spontaneously loaded onto PEG corona in 2 min without the assistance of any catalysts and binding groups. The thermodynamically favourable insertion of the hydrophobic anchor can be reversed by pulling the functional module, enabling programmable surface functionalization. We anticipate that the adaptive molecular recognition between the hydrophobic anchor and the PEG host will challenge the hydrophilic understanding of PEG and enhance the progress in nanomedicine, advanced materials and nanotechnology.

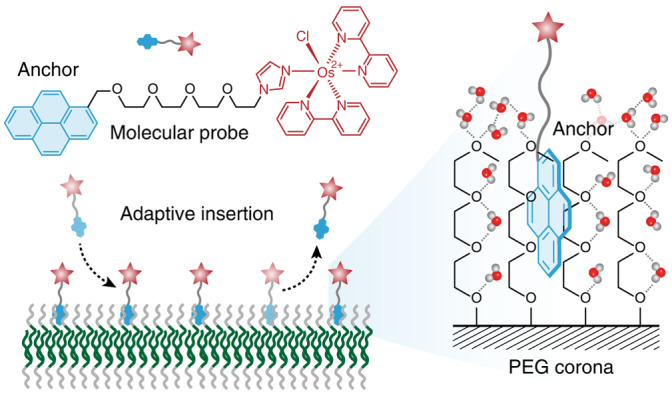

## Abstract (web summary)

Modulation of surface properties and functions can be achieved through covalent and non-covalent molecular binding, but the lack of responsiveness and requirement for specific binding groups makes spatiotemporal control challenging. Now, it has been shown that adaptive insertion of a hydrophobic anchor into a poly(ethylene glycol) host is an effective non-covalent binding strategy for programmable surface functionalization.

## Main

Spatiotemporal selective surface functionalization represents the key step towards next-generation functional systems. Living organisms have already achieved signal transfer, cargo transport, photosynthetic energy conversion and so on by controlling the distribution and orientation of functional proteins on cell membranes^1^. However, it is still a grand challenge for synthetic materials to realize such precise control due to the lack of adaptive binding chemistry^2–4^. Click chemistry provides an efficient binding strategy while sharing the non-responsive nature of the covalent binding strategy^5^. Thanks to the development of the non-covalent binding strategy, biomolecular recognition and host–guest interaction have been used to load therapeutic proteins onto polymeric particles, isolate plasma membrane proteins, load molecular catalysts onto the electrode and so on^6–10^. Although they have proved to be effective, the loaded functional modules can only be released by adding competitive host/guest molecules or through the photoisomerization of the guest molecule, which challenged the re-loading of functional modules, the recycling of host molecule-modified materials and the synthesis of guest molecules^11,12^. Moreover, the host molecules, such as cyclodextrins and cucurbiturils, have to be installed onto the materials’ surface in advance^13^. Engineering materials’ surfaces as the host may enable a simple binding strategy.

**Fig. 1:**
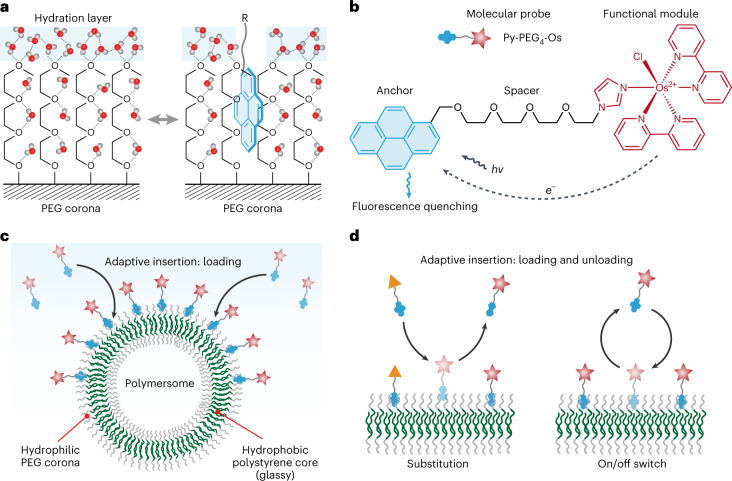
Adaptive insertion of the hydrophobic anchor into the PEG host for programmable surface functionalization. **a**, Schematic showing the insertion of polycyclic aromatic hydrocarbon into PEG corona. **b**, Design of the molecular probe. The probe consists of a Py anchor (which fluoresces in the presence of ultraviolet light) joined to a functional module through a spacer. Electron transfer from the functional module quenches the fluorescence of Py. **c**, Loading of the molecular probe onto PEG corona of polymersomes. The membrane of the polymersomes comprises a hydrophobic polystyrene core and two hydrophilic PEG coronas. **d**, Schematic of adaptive insertion for programmable surface functionalization, showing substitution and the on/off insertion switch.

Polymer and small-molecule ligands are widely used to manipulate the surface chemistry of synthetic materials. For instance, PEG—a hydrophilic and non-toxic polymer—has been engineered into stealth corona on nanocarriers, antifouling coating on implanted materials, polymer electrolytes on porous materials, capping ligand on nanoparticles and so on^14–19^. Inspired by the induced fit model for enzyme–substrate recognition, we believe that the surface-attached PEG chains, similar to the polypeptide chains of enzymes, can adapt to the size and shape of guest molecules and act as the host^20,21^. However, unlike the existence of a hydrophobic cavity in cyclodextrins and cucurbiturils for binding, PEG chains are well hydrated in an aqueous medium by forming hydrogen bonds with water molecules^22–25^. The association of a guest molecule with PEG should require the breakage of these hydrogen bonds and the removal of water molecules from the binding site, just like with protein–ligand binding^26–28^. The dehydration of PEG was thought to be very difficult due to the energy loss in breaking these hydrogen bonds and the chain conformational entropy loss^29^. Thus, although widely existing on materials’ surfaces, PEG has never been explored as the host. Recent simulations and experiments showed that the release of water molecules bonded to PEG can compensate for the chain conformational entropy loss, turning the dehydration of PEG into an energy-driven process^30–32^. Hydrophobic graphene and carbon nanotube composed of *sp*^2^-hybridized carbon atoms successfully drive the dehydration of PEG, suggesting that their binding energy is enough to break the hydrogen bonds^33–35^. We hypothesized that polycyclic aromatic hydrocarbon, bearing a similar chemical structure to graphene, can break the hydration layer and act as the guest molecule (anchor) of the PEG host (Fig. 1a). Unlike the host with the hydrophobic cavity, favourable hydration of the PEG host should allow adaptive insertion of the anchor for programmable surface functionalization.

To test this hypothesis, we designed and synthesized a series of molecular probes comprising polycyclic aromatic hydrocarbons as the anchor and an osmium complex (Os) as the model functional module (Supplementary Figs. 1–9)^36^. Figure 1b shows a representative molecular probe using the hydrophobic pyrenyl group (Py) as the anchor (Py-PEG_4_-Os). Such a design has the following merits: (1) the hydrophilic -PEG_4_-Os can increase the aqueous solubility of Py (Supplementary Figs. 10–12); (2) the polarity-sensitive fluorescence of Py can reflect the position of the anchor^37^; and (3) photoinduced electron transfer from Os to Py can quench the fluorescence of Py and validate the conformation of loaded molecular probe (Supplementary Fig. 13). As for the PEG host, we are interested in the PEG corona of polymersomes with a glassy polystyrene core due to their well-defined structure and high stability (Fig. 1c)^38–41^. We envisioned that the molecular probe can be loaded onto polymersomes by inserting its hydrophobic anchor into PEG corona by forming a van der Waals interaction. The non-covalent interaction should allow the adaptive insertion of the anchor for programmable surface functionalization (Fig. 1d).

## Results and discussion

### Loading of molecular probes onto PEG corona

**Fig. 2:**
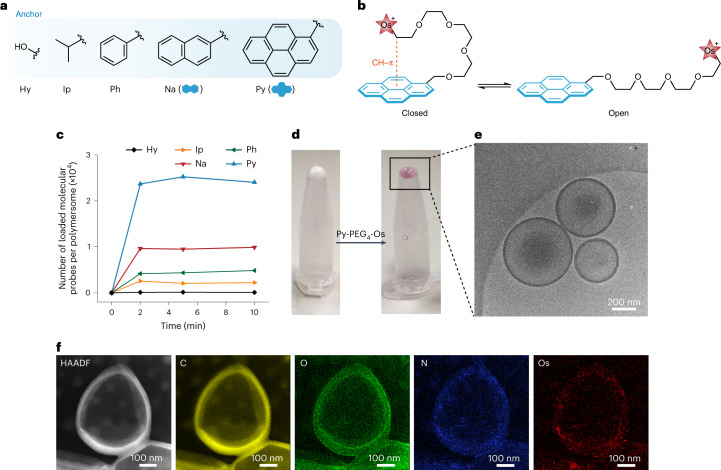
Loading of molecular probes onto PEG corona. **a**, Manipulation of the hydrophobicity of the anchor. The hydrophobicity of the anchor increases from left to right (with the increasing intensity of the blue background). The hydroxyl anchor is the end group of the PEG spacer. The other anchors are linked to the PEG spacer through an ether group. **b**, Conformation of Py-PEG_4_-Os when dissolved in water. The open and closed conformations are in equilibrium. The closed conformation is held together by the CH–π interaction. **c**, Loading process of molecular probes onto polymersomes. Each value corresponds to a mean of *n* = 2 replicates. **d**, Optical images of precipitated polymersomes before and after the modification by Py-PEG_4_-Os. **e**, Cryogenic electron microscopy image of polymersomes loaded with Py-PEG_4_-Os. **f**, High-angle annular dark-field (HAADF) image and elemental mappings of polymersomes loaded with Py-PEG_4_-Os. Source data

First, we tested the loading of molecular probes onto the PEG corona of polymersomes. Here, functional groups with increased hydrophobicity in the order of hydroxyl (Hy) < isopropyl (Ip) < phenyl (Ph) < naphthyl (Na) < Py were tested as the anchor (Fig. 2a and Supplementary Figs. 1–5). Once linked to -PEG_4_-Os with the ether group, the hydrophobic anchors can be dissolved easily in water, even for the most hydrophobic Py (Supplementary Figs. 10–12). The increased aqueous solubility of Py was mainly due to: (1) the intermolecular electrostatic repulsion between positively charged Os, which inhibited the aggregation of Py in water; and (2) the intramolecular CH–π interaction between the PEG_4_ spacer and Py, which reduced the unfavourable interaction between Py and water (Supplementary Fig. 14). No cation–π interaction was detected between Os and Py, which thus did not contribute to the solubilization of Py (Supplementary Fig. 14). Two-dimensional ^1^H–^1^H nuclear Overhauser effect spectroscopy NMR spectra and molecular dynamics simulation showed that the solubilized Py-PEG_4_-Os stays in equilibrium between the closed and open configuration (Fig. 2b and Supplementary Figs. 15–17). Once the molecular probes and polymersomes were mixed in water, Ip-, Ph-, Na- and Py-PEG_4_-Os were loaded onto polymersomes within 2 min, while negligible loading was detected for Hy-PEG_4_-Os (Fig. 2c). This suggested that the hydrophobic anchors, instead of the hydrophilic -PEG_4_-Os, were interacting with the polymersomes. With the increase in anchor hydrophobicity, the number of loaded molecular probes per polymersome became dramatically increased (Fig. 2c). The successful loading of the molecular probe was proved by the red colour of precipitated polymersomes, cryogenic electron microscopy and a positive zeta potential (Fig. 2d,e and Supplementary Fig. 18). Elemental mappings of dried polymersomes successfully detected C and O from polymersomes and N and Os from Py-PEG_4_-Os (Fig. 2f). The packing density of Py-PEG_4_-Os on polymersomes was calculated to be 0.029 ± 0.002 Os per nm^2^, corresponding to a separation distance of 5.9 ± 0.2 nm between each Os (Supplementary Fig. 19). Such a small separation distance reminded us that the electrostatic repulsion between each Os might restrict the packing density of Py-PEG_4_-Os. When NaCl was added to the loading solution to screen the potential electrostatic repulsion, the relative loading amount of Py-PEG_4_-Os was increased from 100 to 186% (Supplementary Fig. 20). For Py-PEG_4_-imidazole (Py-PEG_4_-Im)—the electrically neutral precursor of Py-PEG_4_-Os—the loading amount was not affected by NaCl. This confirmed the role of electrostatic repulsion between each Os in restricting the packing density of Py-PEG_4_-Os on PEG corona. It should be noted that Py-PEG_4_-Os is amphiphilic and the entire study (except isothermal titration calorimetry (ITC)) was carried out at a concentration lower than the critical micelle concentration (Supplementary Fig. 12). The loading behaviour may be influenced at a concentration higher than the critical micelle concentration.

### Loading mechanism of molecular probes

**Fig. 3:**
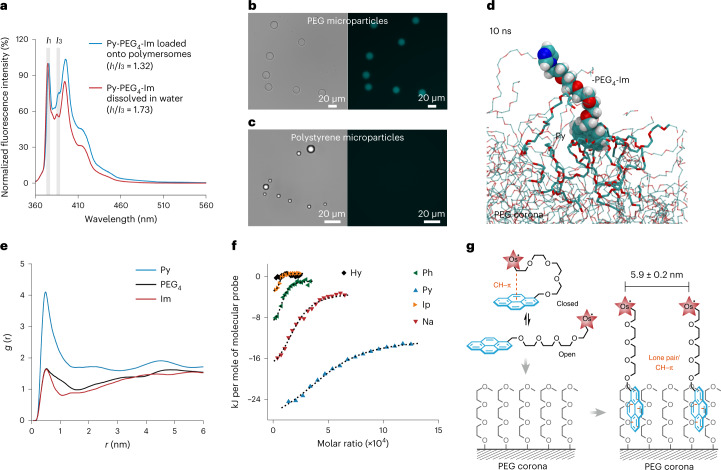
Loading mechanism of molecular probes. **a**, Normalized fluorescence of Py-PEG_4_-Im loaded onto polymersomes and Py-PEG_4_-Im dissolved in water. The fluorescence intensity was normalized at the first fluorescence peak (*I*_1_). **b**,**c**, Brightfield (left) and fluorescence images (right) of the aqueous dispersion of PEG microparticles (**b**) and polystyrene microparticles (**c**) after incubation with Py-PEG_4_-Im. **d**, Snapshot of the loading of Py-PEG_4_-Im onto the PEG corona of polymersomes at 10 ns. Py-PEG_4_-Im is shown as the space-filling model and PEG corona is shown as the framework model (cyan, C; red, O; blue, N; grey, H). **e**, Radial distribution function (*g*(r)) of Py-PEG corona, PEG_4_-PEG corona and Im-PEG corona at 8–10 ns. r is the distance between different segments of molecular probe and PEG corona. **f**, ITC analysis for the binding of molecular probes with polymersomes. The dotted lines represent the fitted plots generated by MicroCal PEAQ-ITC Analysis Software. For Ip, Ph, Na and Py (but not Hy), Δ*H* < 0 and Δ*S* > 0. **g**, Schematic showing the loading of Py-PEG_4_-Os onto PEG corona. Source data

**Extended Data Fig. 1:**
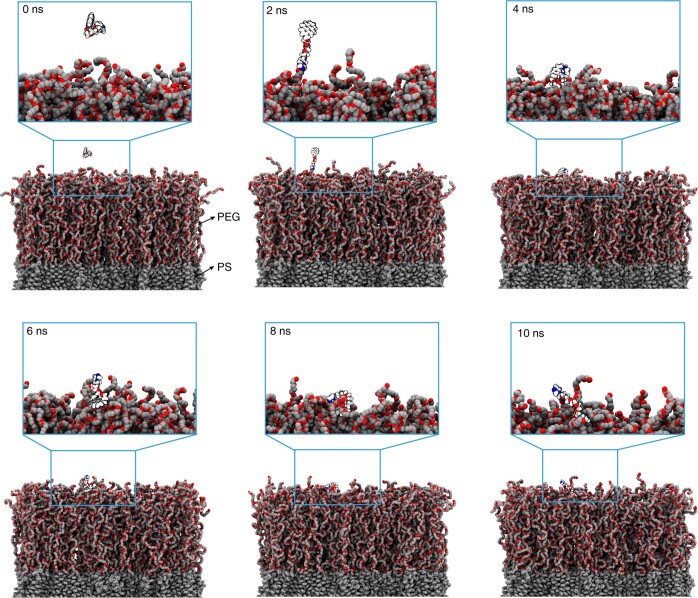
The loading of molecular probe onto PEG corona by molecular dynamics simulation. Representative snapshots at various simulation time. The molecular probe (Py-PEG_4_-Im) is shown as the space-filling model: light grey C; red O; blue N; white H. PEG corona of polymersome is shown as the space-filling model: dark grey C; red O. The dark grey region under PEG corona is PS core.

To investigate the loading mechanism, the fluorescence of Py was monitored during the loading process. Here, Py-PEG_4_-Im was used to exclude the quenching of Py by Os (Supplementary Fig. 13). During the loading process, the fluorescence intensity of Py decreased by ~80% and the ratio of the first and third fluorescence peaks (*I*_1_/*I*_3_) decreased from 1.73 to 1.43 (Supplementary Fig. 21), which indicated a dramatic decrease of polarity around Py^42^. After removing the residual Py-PEG_4_-Im in solution, *I*_1_/*I*_3_ was determined to be 1.32 (Fig. 3a). This was consistent with the literature value for Py in the PEG phase (*I*_1_/*I*_3_ = 1.35) and much higher than Py in polystyrene (*I*_1_/*I*_3_ = 0.95)^43,44^, suggesting insertion of Py into the PEG corona. From the fluorescence image, the loaded molecular probes were homogeneously distributed over the membrane of micrometre-sized polymersomes (Supplementary Fig. 22). To further validate the loading mechanism, PEG microparticles and polystyrene microparticles were used as the host, respectively^45^. After the addition of Py-PEG_4_-Im, PEG microparticles started to emit fluorescence within 5 min (Fig. 3b, Supplementary Video 1 and Supplementary Fig. 23), while no fluorescence was detected on polystyrene microparticles (Fig. 3c). This confirmed the role of the PEG corona in hosting the hydrophobic anchor. The loading mechanism was further studied by molecular dynamics simulation (Extended Data Fig. 1 and Supplementary Videos 2 and 3). Py-PEG_4_-Im was successfully loaded onto the PEG corona in 10 ns by inserting Py into the PEG corona and leaving -PEG_4_-Im on the surface (Fig. 3d). The radical distribution function for Py showed a much higher peak (at ~0.49 nm) than for PEG_4_ and Im (Fig. 3e and Supplementary Fig. 24), suggesting a stronger interaction between Py and PEG corona. Consistently, the interaction energy for Py and PEG corona was higher than for PEG_4_ and Im, confirming the role of Py as the anchor of PEG corona (Supplementary Fig. 25). Moreover, the subunits of PEG (O–CH_2_–CH_2_–O) within the radius of 1.4 nm from the centre of mass of Py retained the *trans*-gauche-*trans* conformation, demonstrating that the insertion of Py did not induce a dramatic conformational change of the PEG corona (Fig. 3d). The insertion process was further studied by ITC (Supplementary Fig. 26). When the aqueous solution of Ip-, Ph-, Na- or Py-PEG_4_-Os was injected into the aqueous dispersion of polymersomes, a positive entropy change (Δ*S* > 0) and negative enthalpy change (Δ*H* < 0) were detected (Fig. 3f and Supplementary Tables 1 and 2). For Hy-PEG_4_-Os, no interaction was detected, again validating that only hydrophobic anchors were binding with PEG corona. During insertion, the formation of a lone pair/CH–π interaction between Py and PEG corona is exothermic (Δ*H* < 0)^35^, whereas the disruption of the well-defined aqueous shell of PEG corona is endothermic (Δ*H* > 0) (Supplementary Fig. 27). The overall negative Δ*H* (−17.9 kJ mol^−1^) suggested that the binding energy between Py and PEG corona was enough to break the aqueous shell of the PEG corona. Moreover, the insertion of Py would restrict the available conformations of molecular probes (Δ*S* < 0), and may simultaneously induce the release of water molecules from the PEG corona (Δ*S* > 0). The overall positive Δ*S* (TΔ*S* = 8.3 kJ mol^−1^) confirmed the release of water molecules from PEG corona (that is, the dehydration of PEG corona). The negative Δ*H* and positive Δ*S* contributed to the thermodynamically favourable insertion of the hydrophobic anchor into the PEG corona of polymersomes (Δ*G* < 0). With the increase of anchor hydrophobicity, a higher binding stoichiometry was obtained (Supplementary Table 1), which was consistent with the adsorption experiments (Fig. 2c). Moreover, the ITC isotherm for Py-PEG_4_-Os did not return to zero at the end of the titration, which should be caused by the dissociation of the aggregates of Py-PEG_4_-Os (1.34 mM) and the additional binding enabled by the accumulated Cl^−^ (Supplementary Figs. 28 and 29).

Based on the above results, we proposed a loading mechanism for Py-PEG_4_-Os onto the PEG corona of polymersomes (Fig. 3g). Solubilized Py-PEG_4_-Os stays in equilibrium between the closed and open configuration. When polymersomes are introduced, Py embeds itself into the PEG corona to escape from the water, leaving the hydrophilic PEG_4_-Os on the surface of the PEG corona. The lone pair/CH–π interaction between Py and PEG corona anchors Py-PEG_4_-Os to the PEG corona and the electrostatic repulsion between each Os determines the packing density of Py-PEG_4_-Os on the PEG corona. Besides PEG-*b*-PS polymersomes and PEG microparticles, the molecular probes were successfully loaded onto poly(ethylene glycol)-*b*-poly(d,l-lactide) (PEG-*b*-PDLLA) polymersomes and PEG-modified gold nanoparticles (Supplementary Figs. 30–34). This highlighted the potential of the insertion strategy to functionalize diverse PEG hosts.

### Conformation of molecular probes loaded onto PEG corona

**Fig. 4:**
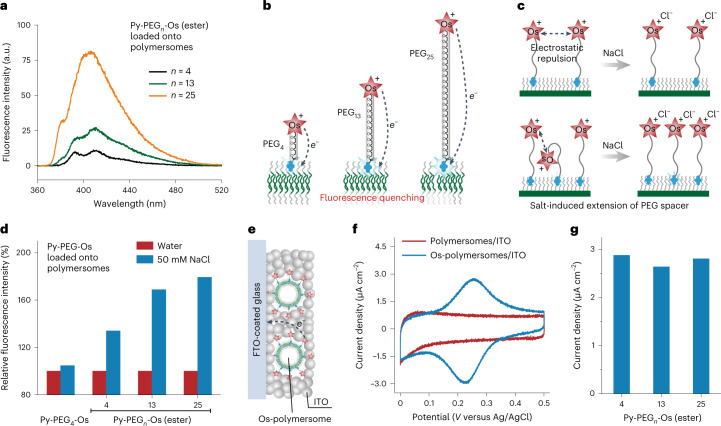
Conformation of molecular probes loaded onto PEG corona. **a**, Fluorescence of Py-PEG_*n*_-Os (ester) loaded onto polymersomes. The loading amount of Py-PEG_*n*_-Os (ester) was the same for the different PEG spacers (*n* = 4, 13 or 25). **b**, Schematic of the fluorescence quenching of inserted Py by photoinduced electron transfer from Os to Py. **c**, Influence of the packing density of molecular probes on the surface accessibility of Os. Salt induced extension of the PEG spacer by screening the electrostatic repulsion between Os. **d**, Fluorescence change of Py-PEG_4_-Os or Py-PEG_*n*_-Os (ester) loaded onto polymersomes by adding NaCl. **e**, Schematic of an electrode prepared by drop-casting the mixture of Os-polymersomes and ITO nanoparticles onto FTO-coated glass. **f**,**g**, Cyclic voltammograms (**f**) and peak current (**g**) of Os-polymersomes/ITO modified electrode. The peak current density is the mean value of three cycles. Source data

The conformation of loaded molecular probes—especially the surface accessibility of functional modules—influences their function^46,47^. To investigate the conformation, we designed different molecular probes: Py-PEG_n_-Os (ester) with an ester bond to link Py and PEG_n_-Os; and Py-Os without the PEG_4_ spacer (Supplementary Figs. 6–9). All of these molecular probes were successfully loaded onto polymersomes, demonstrating the robustness of the insertion of the hydrophobic anchor (Supplementary Fig. 35). Py-PEG_4_-Os (ester) exhibited a higher loading amount than Py-PEG_4_-Os, indicating the increased binding affinity of Py with hydrophobic ester linkage^48^. The loading amount of Py-PEG_n_-Os (ester) was not influenced by the length of PEG_*n*_ spacers (*n* = 4, 13 or 25), allowing us to quantitively compare their fluorescence. As shown in Fig. 4a, the fluorescence intensity of loaded Py-PEG_*n*_-Os became visibly increased with the length of the PEG spacer. Since Os can quench the fluorescence of Py by photoinduced electron transfer, the increased fluorescence should be caused by the enlarged distance between Os and Py owing to the longer PEG_*n*_ spacer (Fig. 4b and Supplementary Fig. 13)^49^. Such distance-sensitive fluorescence allowed us to unravel the conformation of loaded molecular probes by monitoring the fluorescence of inserted Py.

The electrostatic repulsion between each Os has been shown to restrict the packing density of Py-PEG_4_-Os on PEG corona. To achieve a higher packing density for Py-PEG_*n*_-Os (ester), the PEG_*n*_ spacer might be bent to keep the distance between each adjacent Os (Fig. 4c), which would reduce the surface accessibility of Os. To validate the conformation, the fluorescence of loaded molecular probes was monitored when NaCl was added. If PEG_*n*_ spacers were bent, the addition of NaCl would induce their extension by shielding the electrostatic repulsion between each Os, increasing the average distance between Os and Py, and leading to the increased fluorescence (Fig. 4c). For loaded Py-PEG_*n*_-Os (ester), the fluorescence intensity was increased after the addition of NaCl, suggesting that parts of the PEG_*n*_ spacers were bent (Fig. 4d). For loaded Py-PEG_4_-Os, the fluorescence intensity remained almost the same, suggesting that all PEG_4_ spacers were extended. Salt-induced extension of the PEG spacer allowed us to control the surface accessibility of Os under a high packing density (Fig. 4c). To further confirm the surface accessibility, polymersomes loaded with Py-PEG_*n*_-Os (ester) (Os-polymersomes) and indium tin oxide (ITO) nanoparticles were mixed and drop-cast on fluorine-doped tin oxide (FTO)-coated glass (Fig. 4e and Supplementary Fig. 36). Redox peaks of Os were successfully detected using the salt solution as the electrolyte, suggesting that Os could be reached by ITO nanoparticles (Fig. 4f). Moreover, the peak current was not influenced by the length of the PEG spacer, indicating the excellent surface accessibility of Os (Fig. 4g). Electrostatic repulsion between each Os and the lone pair/CH–π interaction between Py and the PEG corona enabled the unique conformation of molecular probes on the PEG corona. Salt-induced extension of the PEG_*n*_ spacer allowed us to control the surface accessibility of Os.

### Adaptive insertion for programmable surface functionalization

**Fig. 5:**
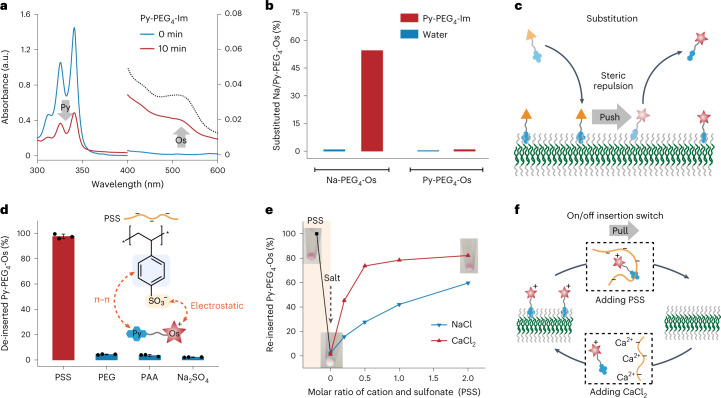
Adaptive insertion for programmable surface functionalization. **a**–**c**, Substitution of molecular probes loaded onto polymersomes. **a**, Loaded Na-PEG_4_-Os was released into solution after the addition of Py-PEG_4_-Im for 0 or 10 min and the absorbance at various wavelengths was measured. The right-hand y-axis represents absorbance (a.u.). The dotted line is the absorbance of the loaded Na-PEG_4_-Os. **b**, Substitution of Na-PEG_4_-Os or Py-PEG_4_-Os loaded onto polymersomes by adding Py-PEG_4_-Im or water. Each value corresponds to a mean of *n* = 2 replicates. **c**, Schematic of the substitution of molecular probes loaded onto polymersomes. **d**–**f**, On/off insertion switch by alternately adding PSS and CaCl_2_. **d**, De-insertion of loaded Py-PEG_4_-Os by adding PSS, PEG, PAA or Na_2_SO_4_. The data are presented as means ± s.d. of *n* = 3 independent experiments. **e**, Re-insertion of Py-PEG_4_-Os by adding CaCl_2_ or NaCl. Each value corresponds to a mean of *n* = 2 replicates. **f**, Schematic of the on/off insertion switch. Source data

The non-covalent interaction with PEG corona should allow adaptive insertion of the hydrophobic anchor for programmable surface functionalization (Fig. 1d). Here, we first investigated the substitution of the loaded molecular probes. Py-PEG_4_-Im and Na-PEG_4_-Os were chosen for the substitution experiment owing to their complementary absorbance and different binding affinity to PEG corona. When Py-PEG_4_-Im was added into the dispersion of polymersomes loaded with Na-PEG_4_-Os, the absorbance of Os in the supernatant increased, while the absorbance of Py decreased (Fig. 5a). This validated the successful substitution of the loaded Na-PEG_4_-Os by Py-PEG_4_-Im. However, Py-PEG_4_-Os loaded onto polymersomes could not be substituted by Py-PEG_4_-Im (Fig. 5b). This demonstrated that the substitution can only occur between molecular probes with different hydrophobic anchors. Py exhibited a higher binding affinity than Na to PEG corona. During substitution, Py-PEG_4_-Im should first be loaded onto the blank region between two loaded Na-PEG_4_-Os molecules and then Na-PEG_4_-Os should be pushed into the solution by steric repulsion (Fig. 5c).

To enable programmable surface functionalization, we investigated the on/off insertion switch of the hydrophobic anchor by introducing the competing reagents of PEG corona (Fig. 5d and Supplementary Fig. 37). Thanks to the glassy polystyrene core, the polymersomes remained stable when incubated with different reagents, including poly(sodium 4-styrenesulfonate) (PSS), PEG, poly(acrylic acid sodium salt) (PAA) or Na_2_SO_4_ (Supplementary Fig. 38). PEG failed in de-inserting the Py-PEG_4_-Os from polymersomes, which should be attributed to its well-hydrated polymer chain. When PSS was added, nearly all of the loaded Py-PEG_4_-Os was released into the solution (Fig. 5d). Since PSS was not adsorbed onto polymersomes (Supplementary Fig. 37), Py-PEG_4_-Os was not substituted, but instead was pulled into the solution by PSS. The pulling force should arise from the π–π interaction between the phenyl group of PSS and Py and the electrostatic attraction between the sulfonate group of PSS and Os (Fig. 5d). PAA or Na_2_SO_4_, which can only pull Py-PEG_4_-Os by electrostatic attraction, failed to induce de-insertion, highlighting the role of multiple interactions. Considering the nature of the pulling force, we tried to use salt to screen the electrostatic attraction between PSS and Py-PEG_4_-Os and to induce the re-insertion of Py-PEG_4_-Os. As shown in Fig. 5e, CaCl_2_ can more efficiently induce the re-insertion of Py-PEG_4_-Os than NaCl, which may be due to stronger interaction between Ca^2+^ and PSS^50^. Above all, PSS can switch off the insertion of Py-PEG_4_-Os by wrapping around it via π–π and electrostatic interaction, and CaCl_2_ can switch on the insertion of Py-PEG_4_-Os by screening electrostatic attraction between Py-PEG_4_-Os and PSS (Fig. 5f). By manipulating the interaction between PSS and Py-PEG_4_-Os, we enabled the on/off insertion switch of the hydrophobic anchor.

## Conclusion

In summary, we report adaptive molecular recognition between the hydrophobic anchor and the PEG host for programmable surface functionalization. This non-covalent binding strategy is distinctive in its high efficiency, short functionalization time, mild conditions, controlled molecular orientation and capability of dynamic loading. We envision that this molecular recognition mode will enable the design of next-generation functional systems and enhance the progress in nanomedicine, nanotechnology and advanced materials.

## Methods

### Synthesis

Molecular probes were synthesized and characterized by NMR, mass spectrometry and Fourier-transform infrared spectroscopy. Details can be found in the Supplementary Information.

### Preparation of polymersomes

Into a 1 ml mixture of tetrahydrofuran and 1,4-dioxane (tetrahydrofuran:1,4-dioxane = 4:1 by volume) was dissolved 10 mg poly(ethylene glycol)-*b*-polystyrene (PEG_44_-*b*-PS_178_). After stirring for 30 min, 1 ml water was injected at a rate of 1 ml h^−1^. Then, 10 ml water was added to quench the polystyrene. After centrifugation and water wash (three times), the polymersomes were dispersed in water. The concentration of polymersomes was measured with a NanoSight LM10 system.

### Loading of the molecular probe onto polymersomes

The aqueous solution of molecular probes (Hy-, Ip-, Ph-, Na- or Py-PEG_4_-Os) was added to the aqueous dispersion of polymersomes. The concentration of molecular probes was ~21 µM and the concentration of polymersomes was 3.8 × 10^−4^ μM in the final mixture. After shaking for a certain time, 300 µl of the mixture was collected and centrifuged to remove the polymersomes. The supernatant was diluted twice with water and measured with an ultraviolet–visible spectrometer. The collected polymersomes were washed with water three times and used for other characterizations.

### Fluorescence measurements

The binding process between Py-PEG_4_-Im and polymersomes was monitored using a JASCO FP-8300ST Spectrofluorometer. Into Py-PEG_4_-Im (4.5 μM; 1.2 ml) was added 5 µl aqueous dispersion of polymersomes (5.6 × 10^−3^ μM) or water 20 times. The excitation wavelength was 342 nm. The excitation and emission bandwidths were 2.5 nm.

### Loading of molecular probe onto PEG and polystyrene microparticles

PEG and polystyrene microparticles were prepared by microfluidic setup and interface precipitation, respectively (see Supplementary Section 6.3). The microparticles were incubated with Py-PEG_4_-Im in water in the chamber slide. The concentration of Py-PEG_4_-Im was 33 μM in the incubation solution. After incubating for at least 10 min, the microparticles were visualized using a Leica DMi8 widefield microscope (excitation: 395 nm). The images were processed with Leica Application Suite X.

### Simulation details

The polymer brush of PEG-*b*-PS was constructed using the Polymer Builder feature of the CHARMM-GUI interface^51^. In total, 44 and 50 monomer units of PEG and polystyrene, respectively, were used to construct the polymer brush configuration. Atom typing, charges and parameters were assigned using the CHARMM General Force Field (CGenFF) program^52^. CHARMM36 (ref. ^53^) all-atom force fields were used for the brush configuration. The resulting PEG-*b*-PS polymer unit was replicated 20 times along the *x* and *y* directions to obtain a configuration containing 400 molecules. These were then energy minimized using the steepest descent method with 5,000 steps and 10 fs time steps. An isothermal–isobaric equilibration was carried out for 10 ns with 2 fs time steps. A constant temperature of 283 K was maintained using the velocity re-scaling method^54^. The pressure was maintained at 1 bar using semi-isotropic pressure coupling by the Berensden method^55^ with a coupling constant of 5 ps. Van der Waals and Coulombic interactions were cut off at 1 nm. Long-range interactions were corrected using the particle mesh Ewald summation^56^ with a grid size of 0.12 nm. Constraints were applied to hydrogen bonds using the LINCS^57^ algorithm. Periodic boundary conditions were applied in all directions. The equilibrated configuration was then fully hydrated by the simple point-charge (SPC) water model^58^ consisting of 123,825 water molecules. An NPT equilibration of the solvated system was carried out for 10 ns with the same set of input parameters as described above.

The initial configuration of Py-PEG_4_-Im was prepared using the PACKMOL^59^ software package, wherein the molecular probe was solvated with 1,150 SPC water molecules. CHARMM36 all-atom force fields were used for the molecular probe and GROMACS-run input topology for the probe was obtained using VMD^60^. An initial energy minimization followed by 100 ns NPT equilibration run was carried out for the molecular probe. The same set of run input parameters was used as described above. The most stable configuration of the molecule probe was inserted in the solvated polymer brush configuration, followed by equilibration. The probe insertion configuration was then allowed to energy minimize followed by a 10 ns NPT equilibration run. All simulations are performed using the GROMACS 2020.3 (ref. ^61^) software package.

### ITC measurements

An aqueous solution of the molecular probe was injected into the aqueous dispersion of polymersomes for ITC measurements. The concentration of polymersomes was 2.10 × 10^−3^ or 2.82 × 10^−3^ μM. The concentration of the molecular probe was 0.490 mM for Hy-PEG_4_-Os, 0.491 mM for Ip-PEG_4_-Os, 0.485 mM for Ph-PEG_4_-Os, 0.696 mM for Na-PEG_4_-Os and 1.340 mM for Py-PEG_4_-Os. Then, 2 µl aqueous solution of molecular probes was injected into the dispersion of polymersomes 20 times. The injection interval was 200 s. The stirring speed was 750 r.p.m. ITC data were analysed with MicroCal PEAQ-ITC Analysis Software.

### Electrochemical characterization

Cyclic voltammograms were performed with a three-electrode system using Ag/AgCl (saturated KCl) as the reference electrode and platinum foil (1.0 cm × 1.0 cm × 0.1 cm) as the counter electrode. The working electrodes were prepared by drop-casting the mixture of Py-PEG_n_-Os (ester)-loaded polymersomes (from ~0.1 mg PEG_44_-*b*-PS_178_) and ITO nanoparticles (0.4 mg) onto FTO-coated glass with Parafilm as spacers for a constant surface area of 1.0 cm × 1.0 cm. The electrolyte solution was composed of 40 mM 2-(N-morpholino)ethanesulfonic acid (MES) and 50 mM KCl (pH = 6.5). Without ITO nanoparticles, no obvious redox peaks of Os could be detected.

### Substitution

Polymersomes (from 4 mg PEG_44_-*b*-PS_178_) were modified with an excess amount of Na-PEG_4_-Os. After being washed with water three times, the polymersomes were dispersed in 0.8 ml water. To this was added 0.4 ml saturated solution of Py-PEG_4_-Im or water. After shaking for 10 min, polymersomes were removed by centrifugation and the supernatant was analysed with an ultraviolet–visible spectrometer to determine the amount of released Na-PEG_4_-Os. The amount of loaded Na-PEG_4_-Os was measured by adding PSS (12.5 mM).

### On/off insertion switch

Polymersomes (from 2 mg PEG_44_-*b*-PS_178_) were modified with an excess amount of Py-PEG_4_-Os. After being washed with water three times, the polymersomes were dispersed in 0.9 ml water. To the above dispersion of polymersomes was added 0.1 ml 100 mM PSS, 100 mM PEG, 100 mM PAA or 100 mM Na_2_SO_4_ (the concentrations of PSS, PEG and PAA refer to the concentration of monomeric units without special notification). After shaking for 10 min, the polymersomes were collected by centrifugation. The supernatant was analysed with the ultraviolet–visible spectrometer to determine the amount of released Py-PEG_4_-Os.

The released Py-PEG_4_-Os was re-loaded onto polymersomes by adding NaCl or CaCl_2_ to the mixture of polymersomes and PSS. After shaking for 10 min, polymersomes were collected by centrifugation and the supernatant was analysed with the ultraviolet–visible spectrometer to determine the amount of re-loaded Py-PEG_4_-Os. By manipulating the concentration of salt, a different molar ratio of cation/sulfonate (PSS) was achieved. The adsorption of PSS on polymersomes in water or NaCl solution was excluded by incubating PSS and polymersomes. The concentration of PSS in the supernatant after incubation was measured with the ultraviolet–visible spectrometer.

## Online content

Any methods, additional references, Nature Portfolio reporting summaries, source data, extended data, supplementary information, acknowledgements, peer review information; details of author contributions and competing interests; and statements of data and code availability are available at 10.1038/s41557-022-01090-0.

## Supplementary information

Supplementary Information Supplementary Figs. 1–38 and Tables 1 and 2.

Supplementary Video 1 Loading of molecular probe onto PEG hydrogel microparticles.

Supplementary Video 2 Molecular dynamics simulation of the loading of molecular probe onto PEG corona in a space-filling model (0–10 ns).

Supplementary Video 3 Molecular dynamics simulation of the loading of molecular probe onto PEG corona in a framework model (0–10 ns).

## Data Availability

All data supporting the findings of this study are available within the paper and its Supplementary Information files. Source data are provided with this paper.

## Source data

Source Data Fig. 2 Numerical data for the plots in panel c.
Source Data Fig. 3 Numerical data for the plots in panels a, e and f.
Source Data Fig. 4 Numerical data for the plots in panels a, d, f and g.
Source Data Fig. 5 Numerical data for the plots in panels a, b, d and e.
