# Supplementary material for: Adaptive insertion of a hydrophobic anchor into a poly(ethylene glycol) host for programmable surface functionalization
Source: Nat Chem. 2022 Nov 21;15(2):240–7. doi: 10.1038/s41557-022-01090-0 (PMC9899690; doi:10.1038/s41557-022-01090-0)
Supplement: Supplementary file 1 — Supplementary Figs. 1–38 and Tables 1 and 2. [file 41557_2022_1090_MOESM1_ESM.pdf]

nature chemistry

Article

https://doi.org/10.1038/s41557-022-01090-0

Adaptive insertion of a hydrophobic anchor into a poly(ethylene glycol) host for programmable surface functionalization

In the format provided by the authors and unedited

Supplementary Information for

Adaptive insertion of a hydrophobic anchor into a poly(ethylene glycol) host for programmable surface functionalization

Shaohua Zhang[1], Wei Li[1], Jiabin Luan[1], Abhinav Srivastava[2,3], Vincenzo Carnevale[2,3], Michael L. Klein[3], Jiawei Sun[1], Danni Wang[1], Serena P. Teora[1], Sjoerd J. Rijpkema[1], Johannes D. Meeldijk[4], Daniela A. Wilson[1]*

[1]Institute for Molecules and Materials, Radboud University, Heyendaalseweg 135, 6525 AJ Nijmegen, The Netherlands

[2]iGEM - Institute for Genomics and Evolutionary Medicine & Department of Biology, Temple University, Philadelphia, Pennsylvania, USA

[3]Institute for Computational Molecular Science, Temple University, Philadelphia, Pennsylvania, USA

[4]Inorganic Chemistry and Catalysis, Debye Institute for Nanomaterials Science, Utrecht University, 3584 CG Utrecht, The Netherlands

*Correspondence to: d.wilson@science.ru.nl

This PDF file includes:

Contents

1. Materials

2. Instruments and characterizations

3. Synthesis of poly(ethylene glycol)-*b*-polystyrene (PEG-*b*-PS)

4. Synthesis and characterization of molecular probes

5. Conformation of molecular probes dissolved in water

6. The loading of molecular probes onto PEG modified surface

7. Conformation of molecular probes loaded onto PEG corona

8. Adaptive loading of molecular probes

9. References

Contents

1. Materials 3

2. Instruments and characterizations.. 3

3. Synthesis of poly(ethylene glycol)-*b*-polystyrene (PEG-*b*-PS).. 4

4. Synthesis and characterization of molecular probes 5

4.1 Hydroxyl-PEG_4-Os (Hy-PEG_4-Os, 1).. 5

4.2 Pyrenyl-PEG_4-Os (Py-PEG_4-Os, 2) 9

4.3 Isopropyl-PEG_4-Os (Ip-PEG_4-Os, 3) 12

4.4 Phenyl-PEG_4-Os (Ph-PEG_4-Os, 4)... 15

4.5 Naphthyl-PEG_4-Os (Na-PEG_4-Os, 5) 18

4.6 Pyrenyl-Os (Py-Os, 6).. 21

4.7 Pyrenyl-PEG_4-Os (ester) (Py-PEG_4-Os (ester), 7).. 23

4.8 Pyrenyl-PEG_{13}-Os (ester) (Py-PEG_{13}-Os (ester), 8) 25

4.9 Pyrenyl-PEG_{25}-Os (ester) (Py-PEG_{25}-Os (ester), 9) 28

5. Conformation of molecular probes dissolved in water 30

5.1 Critical micelle concentration of molecular probes 30

5.2 Fluorescence of molecular probes dissolved in water 34

5.3 Conformation of molecular probes dissolved in water 35

6. The loading of molecular probes onto PEG modified surface................................... 39

6.1 The loading of molecular probes onto PEG-*b*-PS polymersomes 39

6.2 Distribution of molecular probe on polymersomes 44

6.3 The loading of molecular probes onto PEG and PS microparticles............................ 45

6.4 Loading mechanism by molecular dynamics simulation.. 47

6.5 Interaction between molecular probes and PEG-*b*-PS polymersomes 49

6.6 The loading of molecular probes onto PEG-*b*-PDLLA polymersomes......................... 55

6.7 The loading of molecular probes onto PEG modified gold nanoparticles..................... 57

7. Conformation of molecular probes loaded onto PEG corona 63

7.1 The loading of different molecular probes onto PEG-*b*-PS polymersomes 63

7.2 Surface accessibility of the loaded Os 63

8. Adaptive loading of molecular probes 64

8.1 The adsorption of PSS onto PEG-*b*-PS polymersomes 64

8.2 Stability of PEG-*b*-PS polymersomes.. 65

9. References.. 66

1. Materials

Potassium hexachloroosmate(IV) (39% Os, K_2OsCl_6) and sodium dithionite ($Na_2S_2O_4$) were purchased from Acros Organics. 2,2′-Bipyridine (bpy), tetraethylene glycol (PEG_4), *p*-Toluenesulfonyl chloride (TsCl), imidazole, 1-bromo-2-methylpropane, benzyl bromide, 2-(bromomethyl)naphthalene, 1-(bromomethyl)pyrene, 1-pyrenecarboxylic acid (Py-COOH), 1-pyrenemethanol (Py-OH), sodium hydride (NaH, 60% dispersion in mineral oil), dicyclohexylcarbodiimide (DCC), poly(sodium 4-styrenesulfonate) (PSS, average M_w ~70000), poly(acrylic acid sodium salt) (PAA, average Mw ~5100), PEG (average M 380-420, BioUltra 1000, average M_n 2050 Da), indium tin oxide (nanopowder, 30 nm), fluorine-doped tin oxide (FTO) coated glass slide (surface resistivity ~7 Ω/sq), poly(ethylene glycol) diacrylate (PEGDA, M_n 575 Da), potassium thioacetate, and 2-hydroxy-4′-(2-hydroxyethoxy)-2-methylpropiophenone (Irgacure 2959) were purchased from Sigma-Aldrich. Imidazole (Im), and cesium carbonate ($CeCO_3$) were purchased from Fluorochem Ltd. Polystyrene (PS, M_w 20kDa, PDI = 1.02) was purchased from PSS Polymer Standards Service GmbH. Poly(ethylene oxide)-*b*-poly(1,2-butadiene) (PEG_{22}-*b*-PBD_{37}, PDI=1.01) was purchased from Polymer Source Inc. Water used in all experiments was deionized water produced by Millipore instrument.

2. Instruments and characterizations

Instruments: Nuclear Magnetic Resonance (NMR) characterization was carried out on a Bruker AVANCE HD nanobay console with a 9.4 T Ascend magnet (400 MHz). NOESY spectra were measured on a Bruker AVANCE III console with a 11.7 T UltraShield Plus magnet (500 MHz) equipped with a Bruker Prodigy cryoprobe. NMR spectra were recorded at 298 K unless otherwise specified. Chemical shifts are given in parts per million (ppm) to tetramethylsilane (TMS, δ 0.00 ppm) as the internal standard. Coupling constants are reported as J-values in Hz. Mass spectra were measured with Bruker Microflex LRF Maldi-TOF system and JEOL AccuTOF CS JMS-T100CS. FTIR was measured by Shimadzu spirit-T. The absorbance of the molecular probes was measured with JASCO V-630 UV-vis spectrophotometer. Cryo-EM was performed by JEOL TEM 2100. TEM was measured with JEOL JEM-1400 FLASH. Fluorescence was measured with JASCO FP-8300ST Spectrofluorometer. Elemental mappings were acquired on Spectra300 from Thermo Fisher Scientific at an acceleration voltage of 80 kV. ITC was measured with Auto-iTC200. Cyclic voltammograms were measured with Multi Autolab Series instruments (M101).

SEM was measured with JEOL 6330 Cryo Field Emission Scanning Electron Microscope. Size and zeta potential of polymersomes were measured with Malvern DLS-Zetasizer. The concentration of polymersomes was measured with NanoSight LM10. Fluorescent microscopy images were taken with Leica DMi8 widefield microscope (excitation: 395 nm). Confocal z-stack images were taken with Leica SP8 AOBS-beamsplitting white-light laser confocal laser scanning microscope (excitation: 405 nm diode laser).

Characterization:

MALDI-TOF: 1 uL of sample solution (water, 2 mg mL^{-1}), 15 uL of CHCA (ethanol, 10 mg mL^{-1}), and 4 uL of NaTFA (water, 10 mg mL^{-1}) were mixed. 1 μL of the mixed solution was spotted onto the MALDI plate, which was dried before being inserted into the instrument for analysis.

FTIR: Molecular probe was dissolved in chloroform, and placed on IR plates. After drying, FTIR was measured.

3. Synthesis of poly(ethylene glycol)-*b*-polystyrene (PEG-*b*-PS)

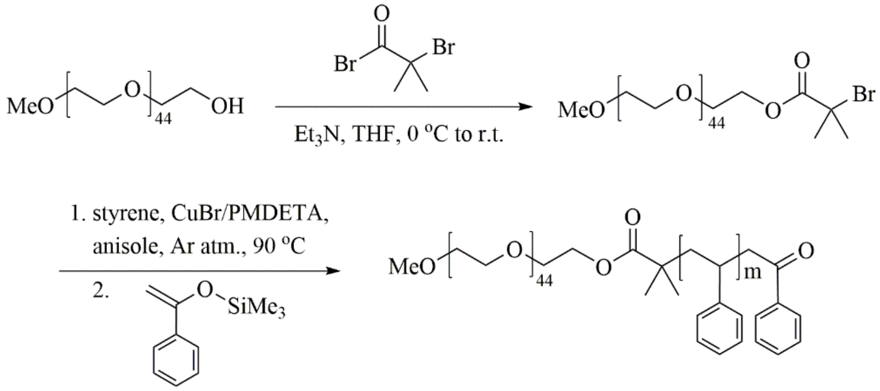

PEG-*b*-PS was synthesized by atom-transfer radical-polymerization (ATRP), as described in our previous publication[1]. The block copolymer was dissolved in $CDCl_3$ for ^{1}H NMR. The length of PS was determined to be 178 by ^{1}H NMR spectrum. PDI of PEG_{44}-*b*-PS_{178} was 1.01 by GPC.

^{1}H NMR (400 MHz, $CDCl_3$): δ 7.24-6.28 (br. m, PS arom.), 3.64 (br. s, PEG backbone), 3.38 (s, 3H, CH_3-O-CH_2), 2.27-1.16 (br. m, PS backbone), 0.88 (br. m, 6H, C(O)-C(CH_3)$_2$-CH_2).

4. Synthesis and characterization of molecular probes

4.1 Hydroxyl-PEG$_4$-Os (Hy-PEG$_4$-Os, 1)

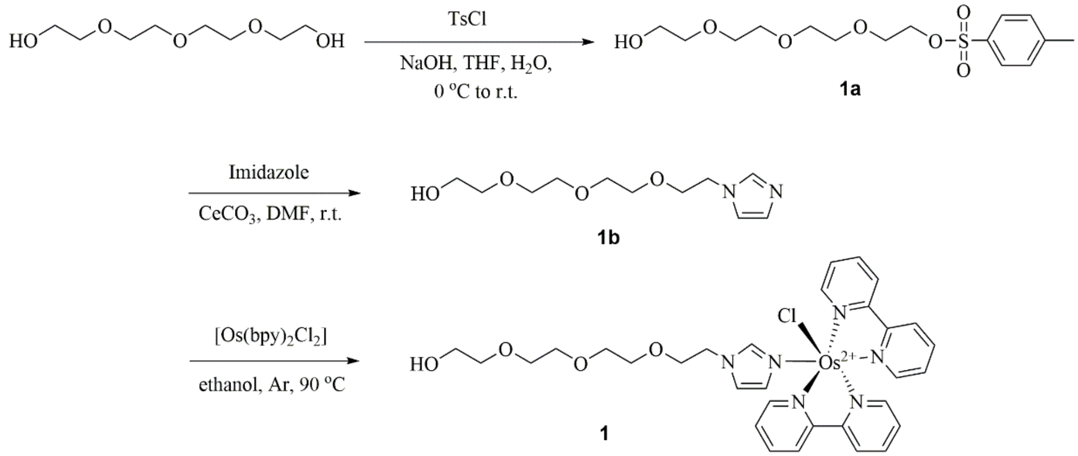

i. Synthesis of [Os(bpy)$_2$Cl$_2$]. K_2OsCl_6 (0.10 g, 0.21 mmol, 1 eq.) and 2,2′-bipyridine (72 mg, 0.46 mmol, 2.2 eq.) were dissolved in dimethylformamide (DMF, 5 mL), which was heated to reflux for 6 h (140 °C, Ar). After cooling to 21 °C, the mixture was centrifuged to remove the precipitate (KCl). Diethyl ether (50 mL) was added dropwise to the supernatant under stirring. After 1 h of stirring at 0 °C, the precipitate ([Os(bpy)$_2$Cl$_2$]Cl) was collected by centrifugation and dried. [Os(bpy)$_2$Cl$_2$]Cl was dissolved in DMF/methanol (1.5 mL, 2:1 v/v). A solution of $Na_2S_2O_4$ (0.10 g, 0.57 mmol, 2.7 eq.) in water (10 mL) was added dropwise under stirring. The mixture was incubated at 0 °C to induce the crystallization of [Os(bpy)$_2$Cl$_2$]. [Os(bpy)$_2$Cl$_2$] was collected by centrifugation, washed with water, methanol, and ethanol, and dried to obtain [Os(bpy)$_2$Cl$_2$] as a black solid (80 mg, 67%). [Os(bpy)$_2$Cl$_2$] was dissolved in dichloromethane (DCM) for UV-vis measurement.

UV-vis (DCM): 240 nm (0.98, $\pi \rightarrow \pi^*$ bpy), 298 nm (1.66, $\pi \rightarrow \pi^*$ bpy), 383 nm (0.36, d → d Os^{2+}), 463 nm (0.33, d → d Os^{2+}), 558 nm (0.37, d → d Os^{2+})[2].

ii. Synthesis of α-tosyl-ω-hydroxyl PEG (PEG$_4$-Ts, 1a). Tetraethylene glycol (PEG$_4$) (20.0 g, 103 mmol, 1 eq.) was dissolved in tetrahydrofuran (THF, 30 mL) and cooled to 0 °C. NaOH (1.38 g, 34 mmol, 0.33 eq.) in water (10 mL) was added, and the resulting mixture was stirred vigorously for 20 min. *p*-Toluenesulfonyl chloride (TsCl) (6.20 g, 32 mmol, 0.31 eq.) in THF (35 mL) was added dropwise over 2 h. The mixture was stirred at 0 °C for 2 h, and then at 21 °C for 20 h. The mixture was then poured into ice-cold water (100 mL), which was extracted with DCM

(4 ×25 mL). The combined organic layer was washed with water (2x), and subsequently with saturated brine (2x) and dried with Na_2SO_4. The solid was filtered off, and the filtrate was concentrated under reduced pressure. The crude product was purified by column chromatography (AcOEt). PEG_4-Ts **1a** was obtained as viscous yellowish oil after being dried *in vacuo* (6.0 g, 54%).

^{1}H NMR (400 MHz, $CDCl_3$) δ 7.80 (d, *J* = 8.3 Hz, 2H, C_{arom}*H*-C-SO_3), 7.34 (d, *J* = 8.3 Hz, 2H, C_{arom}*H*-C-CH_3), 4.16 (t, *J* = 4.9 Hz, 2H, C*H_2*-SO_3), 3.55-3.76 (m, 14H, PEG backbone), 2.45 (s, 3H, -C*H_3*). **^{13}C NMR** (101 MHz, $CDCl_3$) δ 144.8, 133.0, 129.8, 128.0, 72.5, 70.8, 70.7, 70.5, 70.4, 69.3, 68.7, 61.8, 21.7. **MALDI-TOF MS** (m/z): $[M + Na]^+$ Calcd for $C_{15}H_{24}O_7SNa$, 371.114; Found, 371.330.

iii. Synthesis of Hydroxyl-PEG$_4$-Imidazole (Hy-PEG$_4$-Im, 1b). PEG_4-Ts (2.0 g, 5.74 mmol, 1.0 eq.), imidazole (312 mg, 4.58 mmol, 0.8 eq.), and $CeCO_3$ (5.6 g, 17.2 mmol, 3 eq.) were added to DMF (10 mL). The mixture was stirred at 21 °C for 2 days. The mixture was diluted with DCM (30 mL) and filtrated to remove the solid. The mixture was washed with saturated $NaHCO_3$ (2×100 mL) and saturated brine (2×100 mL). The organic layer was dried over Na_2SO_4, and concentrated under reduced pressure. The crude product was purified by column chromatography (gradient, AcOEt → acetone), yielding **1b** as a viscous colorless oil (890 mg, 80%).

^{1}H NMR (400 MHz, $CDCl_3$) δ 7.77 (s, 1H, Im*H*), 7.03 (s, 1H, Im*H*), 6.93 (s, 1H, Im*H*), 4.11 (t, *J* = 5.1 Hz, 2H, C*H_2*-N), 3.57-3.79 (m, 14H, PEG backbone). **MALDI-TOF MS** (m/z): $[M + H]^+$ Calcd for $C_{11}H_{21}O_4N_2$, 245.295; Found, 244.883.

iv. Synthesis of Hydroxyl-PEG$_4$-[Os(bpy)$_2$(Im)Cl] (Hy-PEG$_4$-Os, 1). [$Os(bpy)_2Cl_2$] (33 mg, 57 µmol, 1.1 eq.) and PEG_4-Im (12.8 mg, 52 µmol, 1 eq.) were added to EtOH (8 mL), which was heated to reflux for 6 days (90 °C, Ar). After cooling to 21 °C, the solvent was removed under reduced pressure, and the solid was dissolved in methanol. The obtained solution was centrifuged (14000 rpm, 2 min), and the supernatant was concentrated. Et_2O was added and the obtained solution was incubated at -20 °C, which was centrifuged to obtain the precipitate (2x). After washing with Et_2O and subsequently being dried, the precipitate was further purified by column chromatography (gradient, DCM → DCM/MeOH, 9:1 v/v). The silica that could be potentially washed-out during column chromatography was removed by dissolving the product in DCM and

filtrating through a thin sand layer. Hy-PEG$_4$-Os **1** was obtained as a dark-red solid (33 mg, 77%). Hy-PEG$_4$-Os was dissolved in $CDCl_3$ for ^{1}H NMR and ^{13}C NMR (Supplementary Fig. 1).

^{1}H NMR (400 MHz, $CDCl_3$) δ 9.80 (d, *J* = 5.6 Hz, 1H, bpy), 8.69 (d, *J* = 8.1 Hz, 1H, bpy), 8.59 (d, *J* = 8.1 Hz, 1H, bpy), 8.39 (d, *J* = 5.6 Hz, 1H, bpy), 8.31 (d, *J* = 8.1 Hz, 1H, bpy), 8.15 (d, *J* = 8.1 Hz, 1H, bpy), 7.96 (s, 1H, Im*H*), 7.37-7.71 (m, 8H, bpy), 7.12 (s, 1H, Im*H*), 6.85-7.03 (m, 2H, bpy), 6.52 (s, 1H, Im*H*), 4.16 (s, 2H, CH_2-N), 3.46-3.73 (m, 14H, PEG backbone). **^{13}C NMR** (101 MHz, $CDCl_3$) δ 163.5, 162.1, 161.0, 160.6, 151.6, 151.0, 151.0, 150.5, 138.8, 135.4, 134.2, 134.0, 133.8, 127.8, 127.6, 127.0, 126.6, 126.2, 124.9, 123.7, 122.9, 122.8, 121.9, 72.6, 70.6, 70.4, 70.3, 70.2, 69.8, 61.4, 48.2. **HR-MS** (ESI, positive, methanol) (m/z): $[M]^+$ Calcd for $C_{31}H_{36}O_4N_6ClOs$, 783.21013; Found, 783.20869. **FTIR** $\bar{\upsilon}$ = 3328, 2867, 1598, 1525, 1457, 1418, 1353, 1287, 1254, 1097, 1039, 1012, 935, 886, 824, 764, 725, 658 cm^{-1}.

v. Synthesis of [Os(bpy)$_2$(Im)Cl] (Os). Os was prepared by refluxing the mixture of [Os(bpy)$_2$Cl$_2$] (25.3 mg, 44 µmol, 1.1 eq.) and imidazole (2.7 mg, 40 µmol, 1 eq.) in ethanol. Os was purified by recrystallization and column chromatography (gradient, DCM → DCM/MeOH, 9:1 v/v). The silica that could be potentially washed-out during column chromatography was removed by dissolving the product in DCM and filtrating through a thin sand layer. After drying under a high vacuum, Os was obtained as a dark-red solid (19 mg, 75%).

^{1}H NMR (400 MHz, $CDCl_3$) δ 9.91 (d, *J* = 5.6 Hz, 1H, bpy), 8.29 (d, *J* = 5.6 Hz, 1H, bpy), 8.24 (d, *J* = 8.1 Hz, 1H, bpy), 8.15 (d, *J* = 8.1 Hz, 1H, bpy), 8.06-8.12 (m, 1H, bpy), 8.03 (d, *J* = 8.1 Hz, 1H, bpy), 7.30-7.63 (m, 8H, bpy), 7.18 (s, 1H, Im*H*), 7.09 (s, 1H, Im*H*), 6.99 (s, 1H, Im*H*), 6.77-6.92 (m, 2H, bpy). **^{13}C NMR** (101 MHz, $CDCl_3$) δ 164.0, 162.1, 160.6, 160.3, 151.6, 151.3, 151.0, 150.6, 135.8, 134.8, 134.2, 133.1, 132.6, 127.3, 127.0, 126.9, 126.8, 126.2, 123.6, 122.8, 122.6, 122.4, 118.1. **MALDI-TOF MS** (m/z): $[M + H]^+$ Calcd for $C_{23}H_{21}N_6ClOs$, 608.113; Found, 607.753.

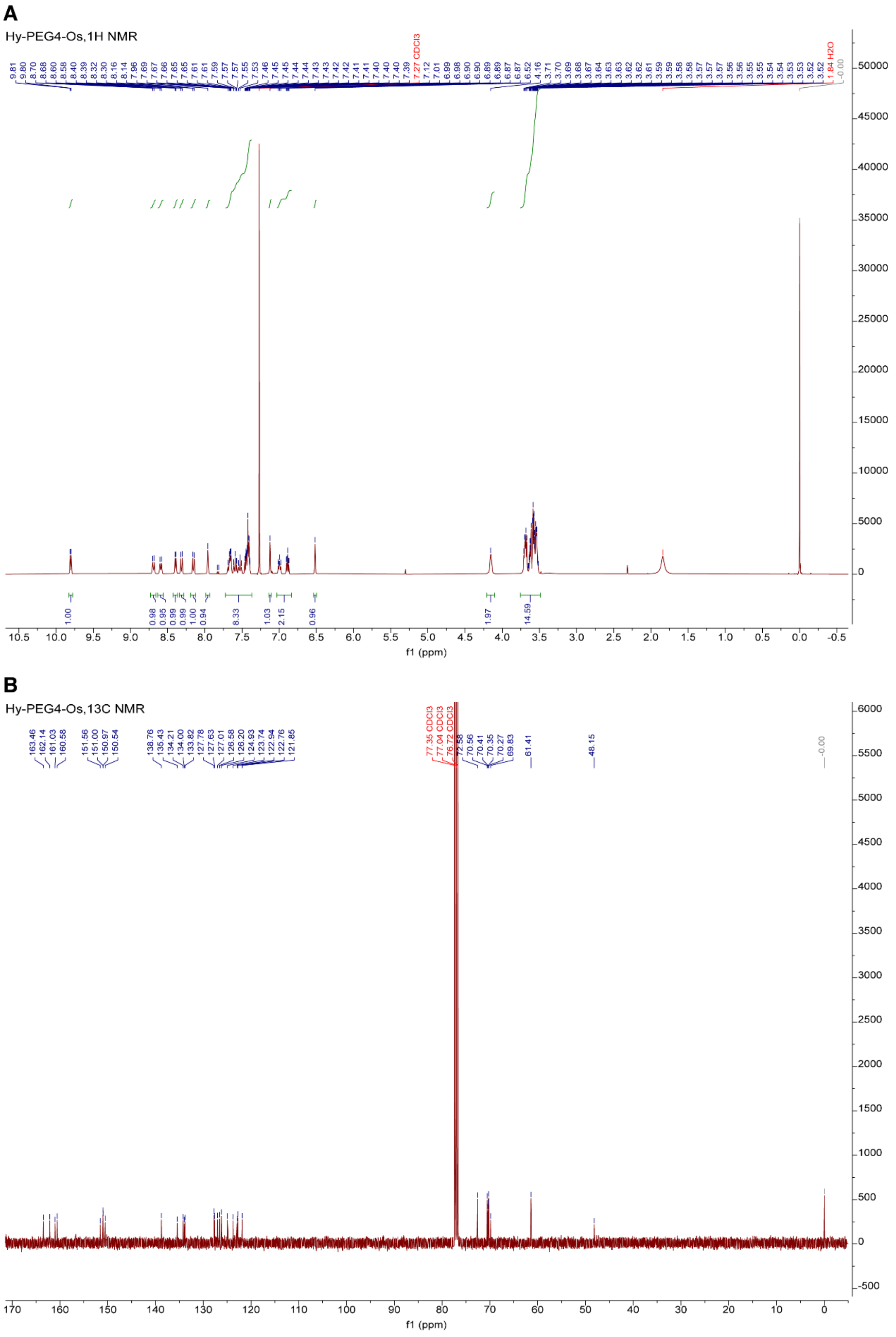

Figure 1. (**A**) ^{1}H NMR and (**B**) ^{13}C NMR spectra of Hy-PEG$_4$-Os in $CDCl_3$.

4.2 Pyrenyl-PEG$_4$-Os (Py-PEG$_4$-Os, 2)

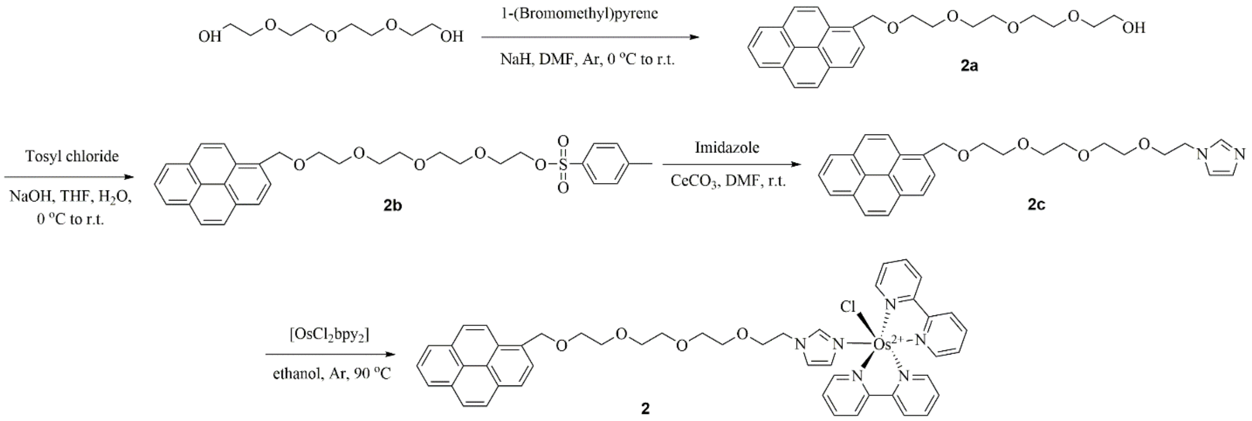

Py-PEG$_4$-Os was synthesized through a similar route as Hy-PEG$_4$-Os, where an additional step for the synthesis of Py-PEG$_4$-OH (**2a**) was added. Briefly, PEG$_4$ (970 mg, 5.0 mmol, 1 eq.) was dissolved in DMF (10 mL), which was cooled to 0 °C. NaH (100 mg, 2.5 mmol, 0.5 eq.), washed with heptane, was added to the solution. After being stirred for 30 min at 0 °C, 1-(bromomethyl)pyrene (502 mg, 2.0 mmol, 0.4 eq.) was added. The mixture was stirred at 0 °C for 1 h, and stirred at 21 °C for 24 h. The reaction was stopped by adding MeOH and water. The mixture was extracted with DCM (3 ×25 mL), and the combined organic layer was washed with saturated $NaHCO_3$ (2×50 mL) and saturated brine (2×50 mL). The organic layer was dried with Na_2SO_4, and concentrated under reduced pressure. The crude product was purified by column chromatography (gradient, DCM → AcOEt), yielding **2a** as a viscous brown oil (613 mg, 75%). Py-PEG$_4$-OH was used for the subsequent reactions analogous to the procedures of Hy-PEG$_4$-Os.

i. Py-PEG$_4$-OH (2a)

^{1}H NMR (400 MHz, $CDCl_3$) δ 8.39 (d, *J* = 9.3 Hz, 1H, Py*H*), 7.94-8.22 (m, 8H, Py*H*), 5.27 (s, 2H, Py-CH_2-O), 3.51-3.79 (m, 16H, (PEG backbone). **^{13}C NMR** (101 MHz, $CDCl_3$) δ [ppm] = 131.3, 131.3, 131.2, 130.8, 129.4, 127.7, 127.4, 127.1, 125.9, 125.2, 124.9, 124.7, 124.5, 123.6, 72.5, 71.8, 70.7, 70.6, , 70.5, 70.3, 69.5, 61.7. **MALDI-TOF MS** (m/z): $[M + H]^+$ Calcd for $C_{25}H_{29}O_5$, 409.202; Found, 408.521.

ii. Py-PEG$_4$-Ts (2b)

^{1}H NMR (400 MHz, $CDCl_3$) δ 8.39 (d, *J* = 9.3 Hz, 1H, Py*H*), 7.97-8.22 (m, 8H, Py*H*), 7.75 (d, *J* = 8.3 Hz, 2H, $C_{arom}H$-C-SO_3), 7.27 (2H, $C_{arom}H$-C-CH_3), 5.27 (s, 2H, Py-CH_2-O), 4.10 (t, *J* = 4.9 Hz, 2H, CH_2-SO_3), 3.47-3.79 (m, 14H, PEG backbone), 2.38 (s, 3H, -CH_3). **^{13}C NMR** (101 MHz, $CDCl_3$) δ 144.7, 133.0, 131.4, 131.3, 131.2, 130.8, 129.8, 129.4, 128.0, 127.7, 127.4, 127.4, 127.1,

126.0, 125.2, 124.9, 124.7, 124.5, 123.6, 71.9, 70.7, 70.6, 70.5, 69.5, 69.2, 68.6, 21.6. **MALDI-TOF MS** (m/z): $[M + H]^+$ Calcd for $C_{32}H_{35}O_7S$, 563.210; Found, 526.894.

iii. Py-PEG$_4$-Im (2c)

^{1}H NMR (400 MHz, $CDCl_3$) δ 8.40 (d, *J* = 9.3 Hz, 1H, Py*H*), 7.97-8.22 (m, 8H, Py*H*), 7.46 (s, 1H, Im*H*), 7.01 (s, 1H, Im*H*), 6.91 (s, 1H, Im*H*), 5.28 (s, 2H, Py-C*H*$_2$-O), 3.96 (t, *J* = 5.2 Hz, 2H, C*H*$_2$-N), 3.45-3.80 (m, 14H, PEG backbone). **^{13}C NMR** (101 MHz, $CDCl_3$) δ 137.5, 131.4, 131.3, 131.3, 130.8, 129.4, 129.2, 127.7, 127.4, 127.1, 126.0, 125.2, 125.2, 125.0, 124.7, 124.5, 123.6, 119.4, 71.9, 70.8, 70.7, 70.6, 70.4, 69.6, 46.9. **MALDI-TOF MS** (m/z): $[M + H]^+$ Calcd for $C_{28}H_{31}O_4N_2$, 459.228; Found, 459.704.

iv. Py-PEG$_4$-Os (2) (Supplementary Fig. 2)

^{1}H NMR (400 MHz, $CDCl_3$) δ 9.76 (d, *J* = 5.6 Hz, 1H, bpy), 8.59 (d, *J* = 8.1 Hz, 1H, bpy), 8.49 (d, *J* = 8.1 Hz, 1H, bpy), 8.37 (d, *J* = 9.3 Hz, 1H, Py*H*), 8.30 (d, *J* = 5.6 Hz, 1H, bpy), 7.97-8.25 (m, 10H, Py*H* and bpy), 7.91 (s, 1H, Im*H*), 7.29-7.61 (m, 8H, bpy), 6.97 (s, 1H, Im*H*), 6.81-6.95 (m, 2H, bpy), 6.41 (s, 1H, Im*H*), 5.25 (s, 2H, Py-C*H*$_2$-O), 4.00 (t, *J* = 4.8 Hz, 2H, C*H*$_2$-N), 3.38-3.79 (m, 14H, PEG backbone). **^{13}C NMR** (101 MHz, $CDCl_3$) δ 163.5, 162.0, 160.9, 160.5, 151.4, 150.9, 150.3, 138.7, 135.3, 134.2, 133.9, 133.8, 131.4, 131.3, 131.2, 130.8, 129.4, 127.7, 127.6, 127.5, 127.5, 127.5, 127.2, 126.9, 126.6, 126.2, 126.1, 125.3, 125.3, 124.9, 124.8, 124.7, 124.5, 123.7, 123.6, 123.0, 122.8, 121.7, 71.8, 70.7, 70.6, 70.5, 70.4, 70.3, 69.6, 48.1. **HR-MS** (ESI, positive, methanol) (m/z): $[M]^+$ Calcd for $C_{48}H_{46}O_4N_6ClOs$, 997.28838; Found, 997.28827. **FTIR** $\bar{\upsilon}$ = 2869, 1598, 1525, 1458, 1418, 1351, 1287, 1254, 1090, 1038, 1013, 946, 849, 821, 748, 660 cm^{-1}.

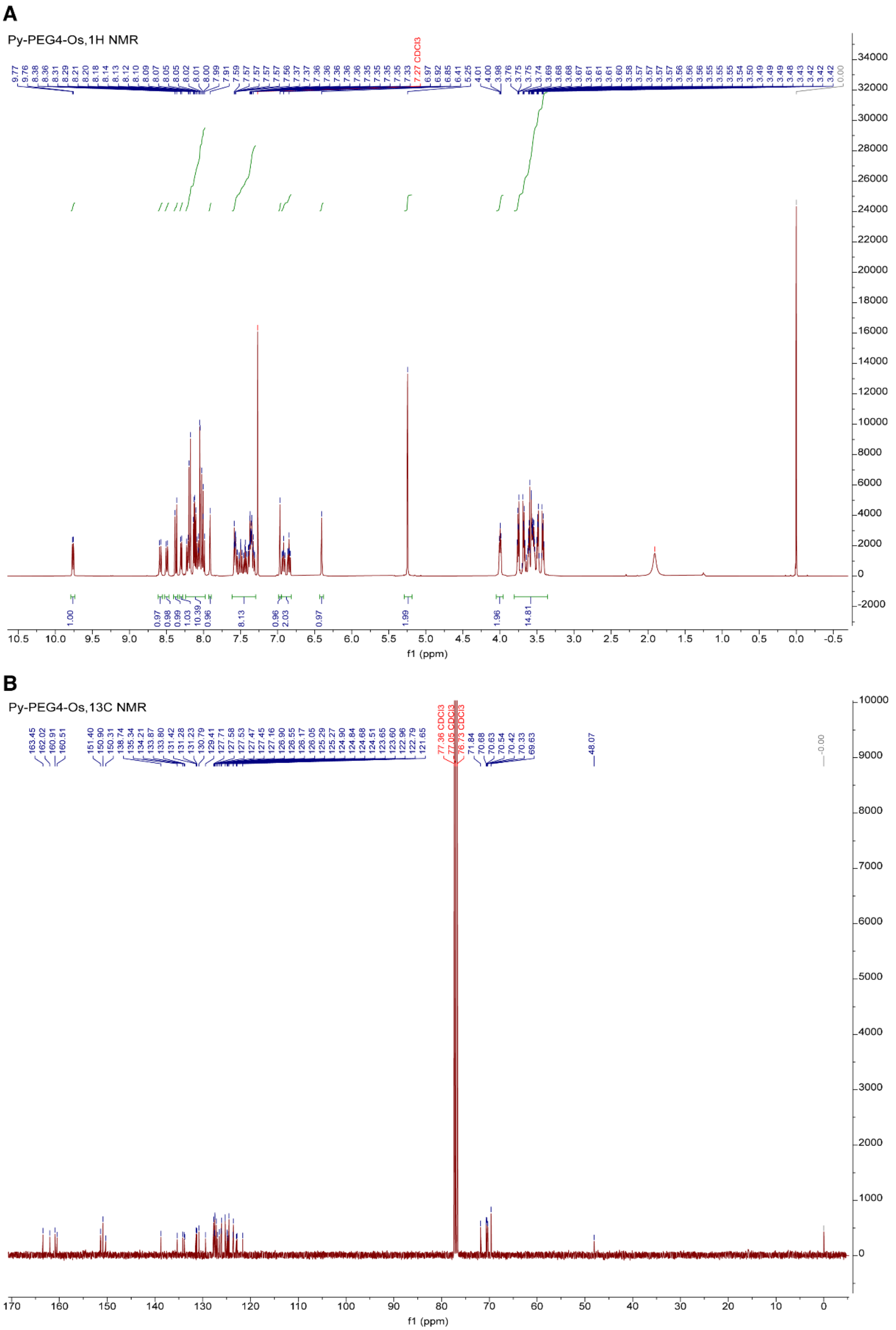

Figure 2. (**A**) ^{1}H NMR and (**B**) ^{13}C NMR spectra of Py-PEG$_4$-Os in $CDCl_3$.

4.3 Isopropyl-PEG4-Os (Ip-PEG4-Os, 3)

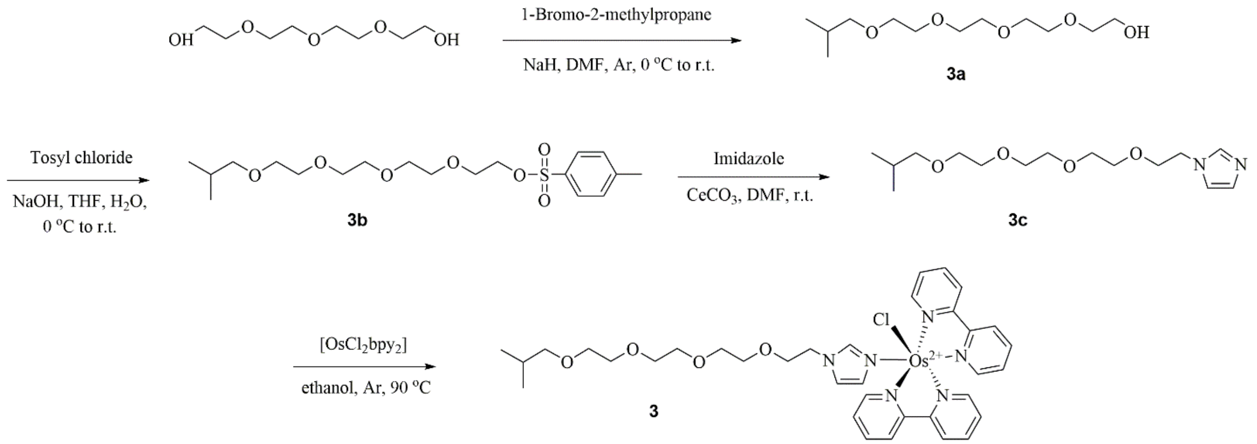

Ip-PEG4-Os (**3**) was synthesized through a similar route as Py-PEG4-Os.

i. Ip-PEG4-OH (3a)

^{1}H NMR (400 MHz, $CDCl_3$) δ 3.77-3.55 (m, 16H, PEG backbone), 3.22 (d, *J* = 6.7 Hz, 2H, CH-C*H*$_2$-O), 2.55 (t, 1H, -O*H*), 1.87 (nonet, *J*= 6.7 Hz, 1H, $(CH_3)_2$-C*H*-CH_2), 0.90 (d, *J* = 6.7 Hz, 6H, (C*H*$_3$)$_2$-CH). **^{13}C NMR** (101 MHz, $CDCl_3$) δ 78.3, 72.5, 70.7, 70.6, 70.4, 70.3, 61.8, 28.3, 19.4. **MALDI-TOF MS** (m/z): $[M + Na]^+$ Calcd for $C_{12}H_{26}O_5Na$, 273.168; Found, 273.019.

ii. Ip-PEG4-Ts (3b)

^{1}H NMR (400 MHz, $CDCl_3$) δ 7.80 (d, *J* = 8.3 Hz, 2H, C_{arom}*H*-C-SO_3), 7.34 (d, *J* = 8.3 Hz, 2H, C_{arom}*H*-C-CH_3), 4.16 (t, *J* = 4.9 Hz, 2H, C*H*$_2$-SO_3), 3.53-3.74 (m, 14H, PEG backbone), 3.21 (d, *J* = 6.7 Hz, 2H, CH-C*H*$_2$-O), 2.45 (s, 3H, -C*H*$_3$), 1.86 (nonet, *J*= 6.7 Hz, 1H, $(CH_3)_2$-C*H*-CH_2), 0.89 (d, *J* = 6.7 Hz, 6H, (C*H*$_3$)$_2$-CH). **^{13}C NMR** (101 MHz, $CDCl_3$) δ [ppm] = 144.8, 133.1, 129.8, 128.0, 78.3, 70.8, 70.7, 70.6, 70.5, 70.3, 69.2, 68.7, 28.3, 21.7, 19.4. **MALDI-TOF MS** (m/z): $[M + Na]^+$ Calcd for $C_{19}H_{32}O_7SNa$, 427.177; Found, 427.550.

iii. Ip-PEG4-Im (3c)

^{1}H NMR (400 MHz, $CDCl_3$) δ 7.53 (s, 1H, Im*H*), 7.04 (s, 1H, Im*H*), 6.99 (s, 1H, Im*H*), 4.11 (t, *J* = 5.2 Hz, 2H, C*H*$_2$-N), 3.54-3.79 (m, 14H, PEG backbone), 3.22 (d, *J* = 6.7 Hz, 2H, CH-C*H*$_2$-O), 1.87 (nonet, *J*= 6.7 Hz, 1H, $(CH_3)_2$-C*H*-CH_2), 0.89 (d, *J* = 6.7 Hz, 6H, (C*H*$_3$)$_2$-CH). **^{13}C NMR** (101 MHz, $CDCl_3$) δ 137.5, 129.3, 119.4, 78.3, 70.8, 70.7, 70.6, 70.5, 70.3, 47.1, 28.3, 19.4. **MALDI-TOF MS** (m/z): $[M + H]^+$ Calcd for $C_{15}H_{29}O_4N_2$, 301.213; Found, 301.174.

iv. Ip-PEG4-Os (3) (Supplementary Fig. 3)

^{1}H NMR (400 MHz, $CDCl_3$) δ 9.81 (d, *J* = 5.6 Hz, 1H, bpy), 8.64 (d, *J* = 8.1 Hz, 1H, bpy), 8.54 (d, *J* = 8.1 Hz, 1H, bpy), 8.38 (d, *J* = 5.6 Hz, 1H, bpy), 8.32 (d, *J* = 8.1 Hz, 1H, bpy), 8.16 (d, *J* =

8.1 Hz, 1H, bpy), 7.95 (s, 1H, Im*H*), 7.36-7.70 (m, 8H, bpy), 7.06 (s, 1H, Im*H*), 6.85-7.02 (m, 2H, bpy), 6.51 (s, 1H, Im*H*), 4.14 (t, J = 4.8 Hz, 2H, CH_2-N), 3.48-3.73 (m, 14H, PEG backbone), 3.20 (d, J = 6.7 Hz, 2H, CH-CH_2-O), 1.85 (nonet, J= 6.7 Hz, 1H, $(CH_3)_2$-C*H*-CH_2), 0.88 (d, J = 6.7 Hz, 6H, $(CH_3)_2$-CH). **^{13}C NMR** (101 MHz, $CDCl_3$) δ 163.5, 162.1, 161.0, 160.6, 151.5, 151.0, 150.4, 138.8, 135.4, 134.2, 133.9, 133.9, 127.8, 127.6, 127.0, 126.6, 126.2, 124.8, 123.6, 123.0, 122.8, 121.7, 78.3, 70.6, 70.5, 70.4, 70.3, 69.8, 48.2, 28.3, 19.4. **HR-MS** (ESI, positive, methanol) (m/z): $[M]^+$ Calcd for $C_{35}H_{44}O_4N_6ClOs$, 839.27273; Found, 839.27228. **FTIR** ῡ = 3391, 2869, 1690, 1599, 1526, 1459, 1418, 1355, 1288, 1256, 1199, 1100, 1040, 1013, 948, 822, 768, 724, 660 cm^{-1}.

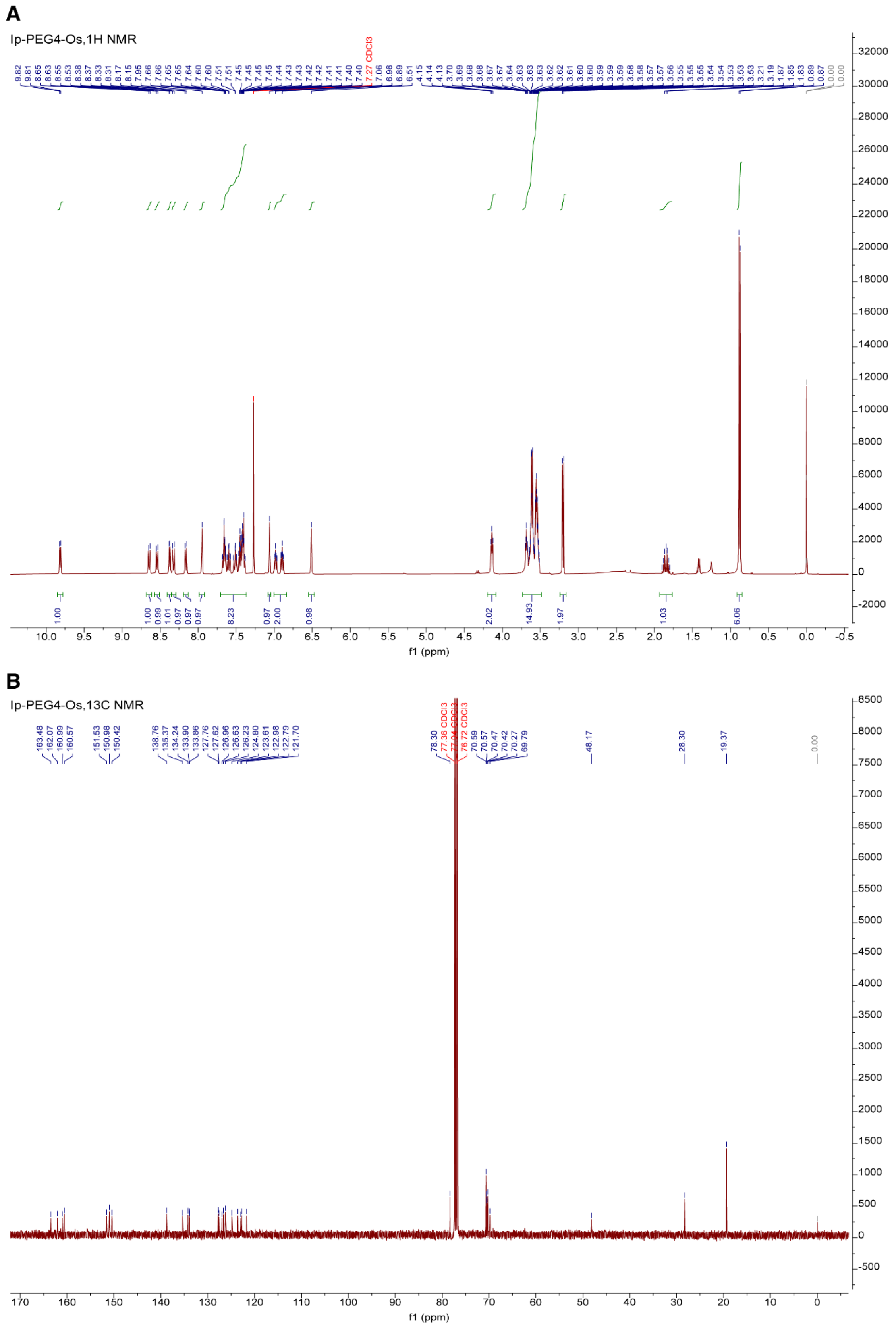

Figure 3. (**A**) ^{1}H NMR and (**B**) ^{13}C NMR spectra of Ip-PEG$_4$-Os in $CDCl_3$.

4.4 Phenyl-PEG$_4$-Os (Ph-PEG$_4$-Os, 4)

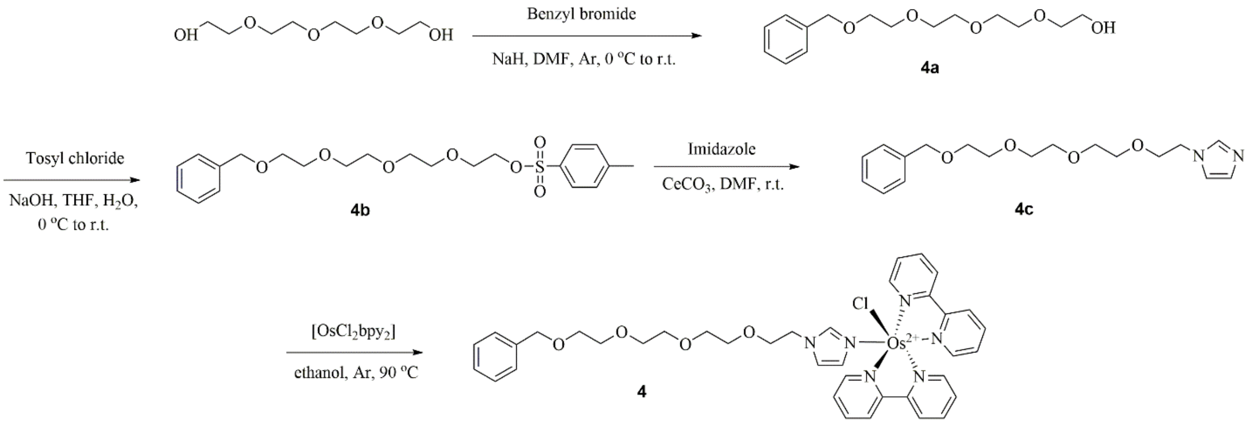

Ph-PEG$_4$-Os (**4**) was synthesized through a similar route as Py-PEG$_4$-Os.

i. Ph-PEG$_4$-OH (4a)

^{1}H NMR (400 MHz, $CDCl_3$) δ 7.27-7.38 (m, 5H, Ph*H*), 4.57 (s, 2H, Ph-C*H*$_2$-O), 3.57-3.75-3.57 (m, 16H, PEG backbone), 2.46 (t, 1H, -O*H*). **^{13}C NMR** (101 MHz, $CDCl_3$) δ 138.3, 128.4, 127.8, 127.6, 73.3, 72.5, 70.7, 70.6, 70.4, 69.5, 61.8. MALDI-TOF MS (m/z): $[M + Na]^+$ Calcd for $C_{15}H_{24}O_5Na$, 307.152; Found, 307.186.

ii. Ph-PEG$_4$-Ts (4b)

^{1}H NMR (400 MHz, $CDCl_3$) δ 7.79 (d, J = 8.3 Hz, 2H, C_{arom}*H*-C-SO_3), 7.26-7.36 (m, 7H, Ph*H* and C_{arom}*H*-C-CH_3), 4.56 (s, 2H, Ph-C*H*$_2$-O), 4.15 (t, J = 4.9 Hz, 2H, C*H*$_2$-SO_3), 3.53-3.71 (m, 14H, PEG backbone), 2.44 (s, 3H, -C*H*$_3$). **^{13}C NMR** (101 MHz, $CDCl_3$) δ 144.8, 138.3, 133.1, 129.8, 128.4, 128.0, 127.7, 127.6, 73.3, 70.8, 70.7, 70.6, 69.5, 69.2, 68.7, 21.6. **MALDI-TOF MS** (m/z): $[M + Na]^+$ Calcd for $C_{22}H_{30}O_7SNa$, 461.161; Found, 461.636.

iii. Ph-PEG$_4$-Im (4c)

^{1}H NMR (400 MHz, $CDCl_3$) δ 7.52 (s, 1H, Im*H*), 7.27-7.36 (m, 5H, Ph*H*), 7.03 (s, 1H, Im*H*), 6.98 (s, 1H, Im*H*), 4.56 (s, 2H, Ph-C*H*$_2$-O), 4.08 (t, J = 5.2 Hz, 2H, C*H*$_2$-N), 3.54-3.76 (m, 14H, PEG backbone). **^{13}C NMR** (101 MHz, $CDCl_3$) δ 138.3, 137.6, 129.3, 128.4, 127.8, 127.6, 119.4, 73.3, 70.7, 70.6, 70.5, 69.5, 47.0. **MALDI-TOF MS** (m/z): $[M + H]^+$ Calcd for $C_{18}H_{27}O_4N_2$, 335.197; Found, 335.279.

iv. Ph-PEG$_4$-Os (4) (Supplementary Fig. 4)

^{1}H NMR (400 MHz, $CDCl_3$) δ 9.80 (d, J = 5.6 Hz, 1H, bpy), 8.68 (d, J = 8.1 Hz, 1H, bpy), 8.59 (d, J = 8.1 Hz, 1H, bpy), 8.38 (d, J = 5.6 Hz, 1H, bpy), 8.29 (d, J = 8.1 Hz, 1H, bpy), 8.14 (d, J = 8.1 Hz, 1H, bpy), 7.95 (s, 1H, Im*H*), 7.36-7.70 (m, 8H, bpy), 7.27-7.36 (m, 5H, Ph*H*), 7.05 (s, 1H,

Im*H*), 6.84-7.02 (m, 2H, bpy), 6.53 (s, 1H, Im*H*), 4.54 (s, 2H, Ph-CH_2-O), 4.11 (t, *J* = 4.8 Hz, 2H, CH_2-N), 3.46-3.72 (m, 14H, PEG backbone). **^{13}C NMR** (101 MHz, $CDCl_3$) δ 163.5, 162.1, 161.0, 160.6, 151.5, 151.0, 151.0, 150.5, 138.8, 138.3, 135.4, 134.1, 133.8, 128.4, 127.8, 127.7, 127.0, 126.6, 126.2, 125.0, 123.8, 122.9, 122.7, 121.7, 73.3, 70.6, 70.6, 70.5, 70.4, 69.8, 69.5, 48.2. **HR-MS** (ESI, positive, methanol) (m/z): $[M]^+$ Calcd for $C_{38}H_{42}O_4N_6ClOs$, 873.25708; Found, 873.25742. **FTIR** ῡ = 3383, 2866, 1689, 1599, 1526, 1457, 1418, 1354, 1287, 1256, 1203, 1096, 1036, 1013, 939, 821, 765, 728, 701, 660 cm^{-1}.

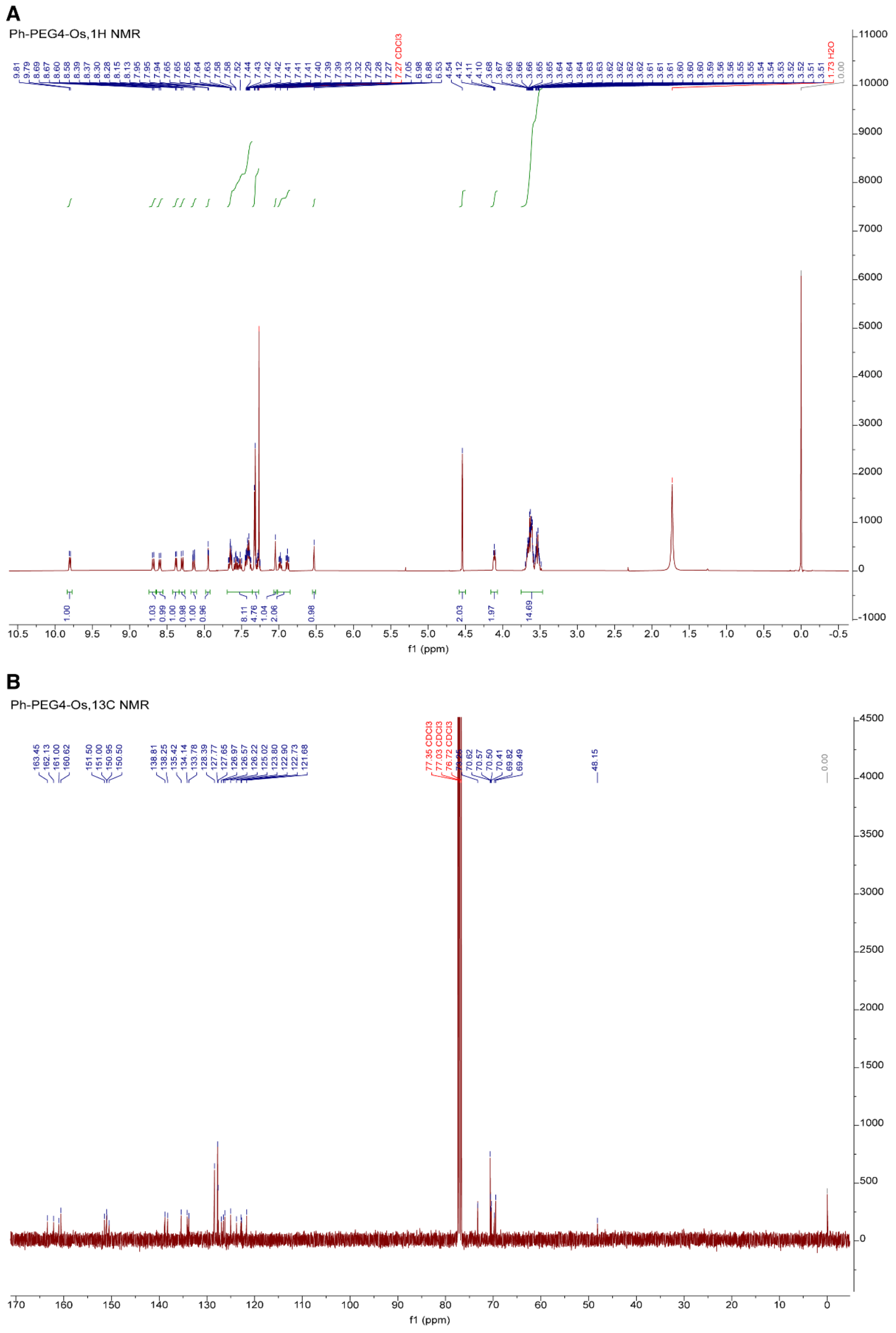

Figure 4. (**A**) ^{1}H NMR and (**B**) ^{13}C NMR spectra of Ph-PEG$_4$-Os in $CDCl_3$.

4.5 Naphthyl-PEG$_4$-Os (Na-PEG$_4$-Os, 5)

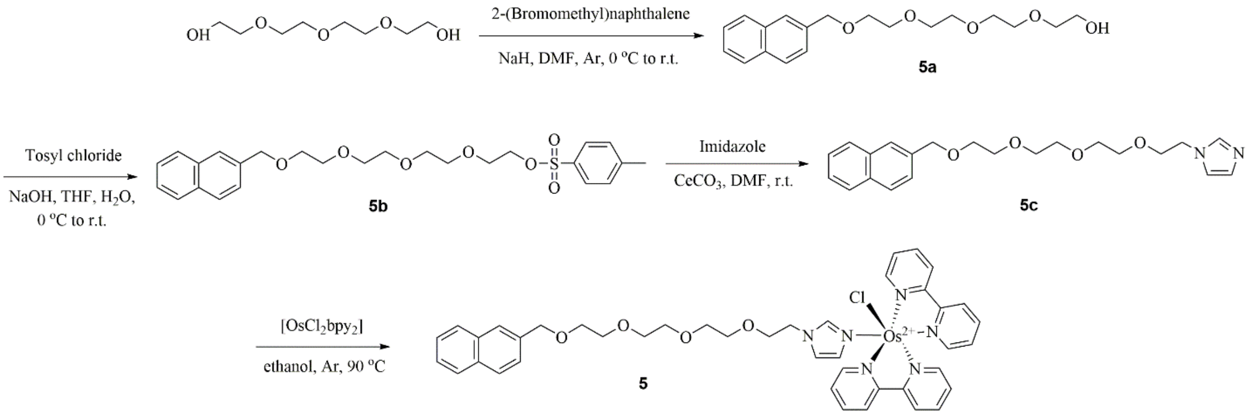

Na-PEG$_4$-Os (**5**) was synthesized through a similar route as Py-PEG$_4$-Os.

i. Na-PEG$_4$-OH (5a)

^{1}H NMR (400 MHz, CDCl$_3$) δ 7.76-7.86 (m, 4H, Na*H*), 7.42-7.51 (m, 3H, Na*H*), 4.73 (s, 2H, Na-C*H*$_2$-O), 3.56-3.74 (m, 16H, (C*H*$_2$-C*H*$_2$-O)$_4$), 2.50 (s, 1H, -O*H*). **^{13}C NMR** (101 MHz, CDCl$_3$) δ 135.8, 133.3, 133.0, 128.1, 127.9, 127.7, 126.5, 126.1, 125.8, 125.8, 73.4, 72.5, 70.7, 70.6, 70.4, 69.5, 61.8. **MALDI-TOF MS** (m/z): $[M + Na]^+$ Calcd for $C_{19}H_{26}O_5Na$, 357.168; Found, 357.358.

ii. Na-PEG$_4$-Ts (5b)

^{1}H NMR (400 MHz, CDCl$_3$) 7.75-7.87 (m, 6H, Na*H* and C$_{arom}$*H*-C-SO$_3$), 7.42-7.51 (m, 3H, Na*H*), 7.30 (d, *J* = 8.3 Hz, 2H, C$_{arom}$*H*-C-CH$_3$), 4.72 (s, 2H, Na-C*H*$_2$-O), 4.13 (t, *J* = 4.9 Hz, 2H, C*H*$_2$-SO$_3$), 3.53-3.73 (m, 14H, PEG backbone), 2.42 (s, 3H, -C*H*$_3$). **^{13}C NMR** (101 MHz, CDCl$_3$) δ 144.8, 135.8, 133.3, 133.1, 133.0, 129.8, 128.1, 128.0, 127.9, 127.7, 126.5, 126.1, 125.8, 125.8, 73.3, 70.8, 70.7, 70.6, 70.5, 69.5, 69.2, 68.7, 21.6. **MALDI-TOF MS** (m/z): $[M + Na]^+$ Calcd for $C_{26}H_{32}O_7SNa$, 511.177; Found, 511.718.

iii. Na-PEG$_4$-Im (5c)

^{1}H NMR (400 MHz, CDCl$_3$) δ 7.76-7.85 (m, 4H, Na*H*), 7.51 (s, 1H, Im*H*), 7.43-7.50 (m, 3H, Na*H*), 7.03 (s, 1H, Im*H*), 6.96 (s, 1H, Im*H*), 4.73 (s, 2H, Na-C*H*$_2$-O), 4.04 (t, *J* = 5.2 Hz, 2H, C*H*$_2$-N), 3.52-3.78 (m, 14H, PEG backbone). **^{13}C NMR** (101 MHz, CDCl$_3$) δ 137.5, 135.8, 133.3, 133.0, 129.2, 128.1, 127.9, 127.7, 126.5, 126.1, 125.9, 125.8, 119.4, 73.4, 70.7, 70.7, 70.6, 70.5, 69.5, 47.0. **MALDI-TOF MS** (m/z): $[M + H]^+$ Calcd for $C_{22}H_{29}O_4N_2$, 385.213; Found, 385.444.

iv. Na-PEG$_4$-Os (5) (Supplementary Fig. 5)

^{1}H NMR (400 MHz, CDCl$_3$) δ 9.77 (d, *J* = 5.6 Hz, 1H, bpy), 8.67 (d, *J* = 8.1 Hz, 1H, bpy), 8.60 (d, *J* = 8.1 Hz, 1H, bpy), 8.34 (d, *J* = 5.6 Hz, 1H, bpy), 8.26 (d, *J* = 8.1 Hz, 1H, bpy), 8.11 (d, *J* =

8.1 Hz, 1H, bpy), 7.94 (s, 1H, Im*H*), 7.75-7.86 (m, 4H, Na*H*), 7.33-7.68 (m, 11H, Na*H* and bpy), 7.03 (s, 1H, Im*H*), 6.83-7.01 (m, 2H, bpy), 6.51 (s, 1H, Im*H*), 4.70 (s, 2H, Na-CH_2-O), 4.09 (t, *J* = 4.8 Hz, 2H, CH_2-N), 3.46-3.70 (m, 14H, PEG backbone**).** ^{13}**C NMR** (101 MHz, $CDCl_3$) δ 163.4, 162.1, 161.0, 160.6, 151.5, 151.0, 150.9, 150.5, 138.8, 135.8, 135.4, 134.1, 133.8, 133.3, 133.0, 128.2, 127.9, 127.7, 127.7, 127.0, 126.6, 126.5, 126.2, 126.2, 125.9, 125.8, 125.0, 122.9, 122.7, 121.7, 73.3, 70.6, 70.6, 70.5, 70.4, 69.8, 69.5, 48.1. **HR-MS** (ESI, positive, methanol) (m/z): $[M]^+$ Calcd for $C_{42}H_{44}O_4N_6ClOs$, 923.27273; Found, 923.27374. **FTIR** $\bar{\upsilon}$ = 3388, 2867, 1599, 1526, 1516, 1458, 1418, 1354, 1287, 1256, 1096, 1039, 1015, 949, 859, 822, 765, 727, 700, 660 cm^{-1}.

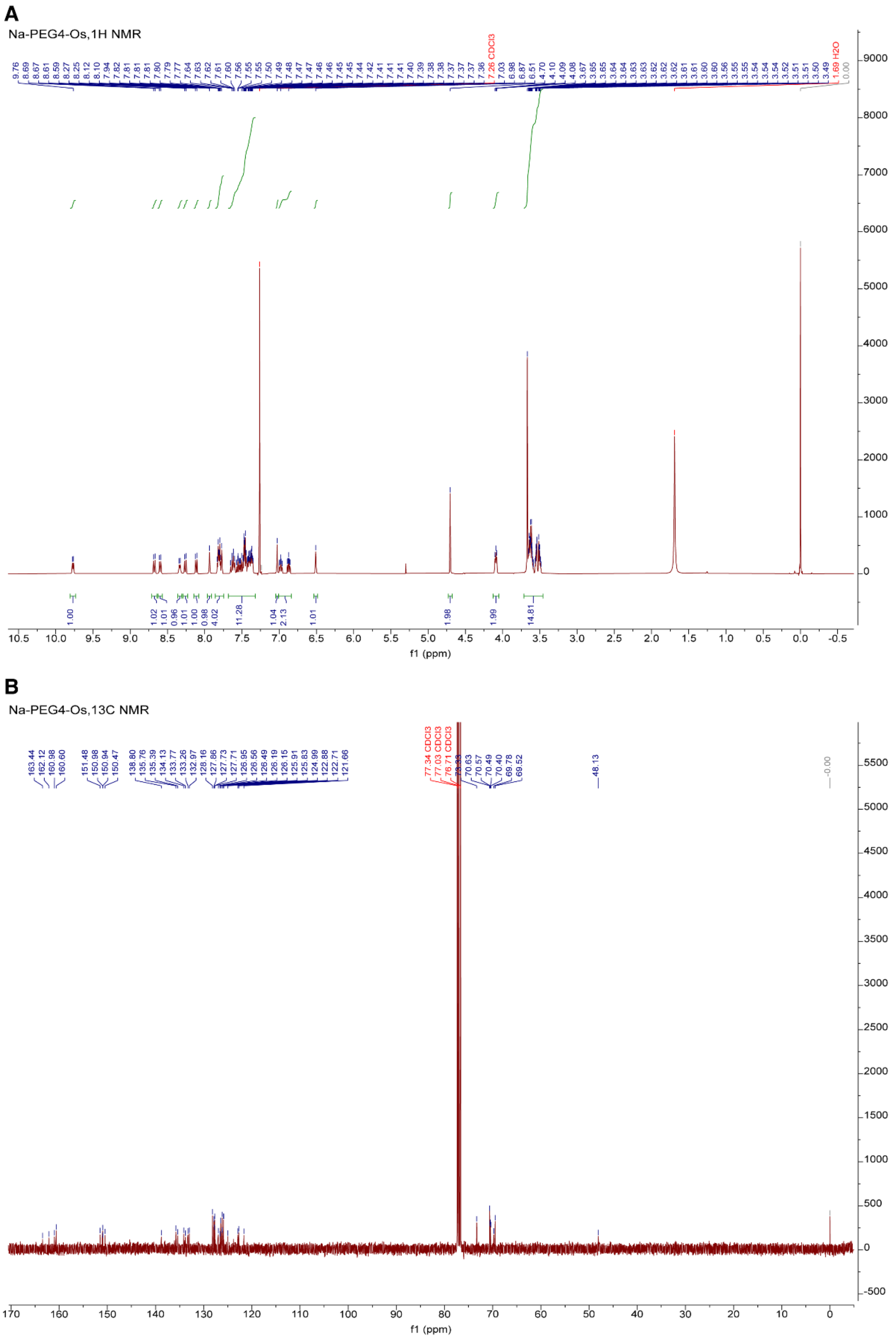

Figure 5. (**A**) ^{1}H NMR and (**B**) ^{13}C NMR spectra of Na-PEG$_4$-Os in $CDCl_3$.

4.6 Pyrenyl-Os (Py-Os, 6)

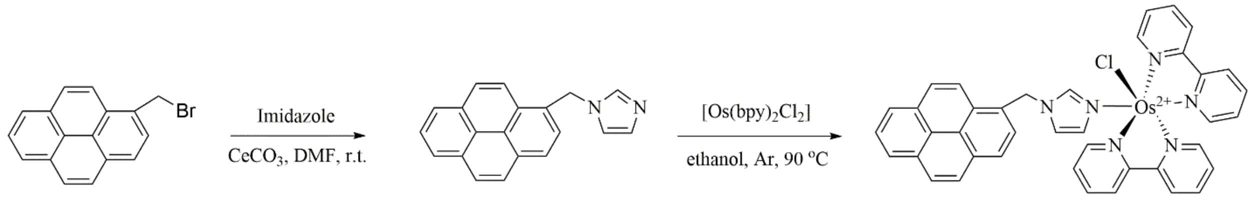

6a 6

1-(bromomethyl)pyrene (59 mg, 0.20 mmol, 1.3 eq.) was dissolved in DMF (2 mL). Imidazole (11 mg, 0.16 mmol, 1 eq) and $CeCO_3$ (196 mg, 0.60 mmol, 3.8 eq.) were added. After stirring for 24 h, DCM was added and the resulting dispersion was filtrated to remove the salt. Py-Im was purified by column chromatography (gradient, DCM→DCM/AcOEt, 1:1 v/v). Py-Im **6a** was obtained as a yellow solid (29 mg, 64%). Py-Os was prepared by refluxing Py-Im (5.6 mg, 20 μmol, 1.0 eq.) and $[Os(bpy)_2Cl_2]$ (12.6 mg, 22 μmol, 1.1 eq.) in EtOH. Py-Os was purified by column chromatography (gradient, DCM→DCM/MeOH, 9:1 v/v). Py-Os **6** was obtained as a dark-red solid (12 mg, 70%).

i. Py-Im (6a)

^{1}H NMR (400 MHz, $CDCl_3$) δ 8.01-8.27 (m, 8H, Py*H*), 7.74 (d, *J* = 7.8 Hz, 1H, Py*H*), 7.63 (s, 1H, Im*H*), 7.10 (s, 1H, Im*H*), 6.95 (s, 1H, Im*H*), 5.85 (s, 2H, Py-C*H_2*-N). **^{13}C NMR** (101 MHz, $CDCl_3$) δ 137.5, 131.7, 131.3, 130.6, 129.8, 128.8, 128.7, 128.4, 128.0, 127.3, 126.5, 126.3, 125.9, 125.7, 125.0, 125.0, 124.6, 121.7, 119.4, 48.9. **MALDI-TOF MS** (m/z): $[M + H]^+$ Calcd for $C_{20}H_{15}N_2$, 283.124; Found, 281.991.

ii. Py-Os (6) (Supplementary Fig. 6)

^{1}H NMR (400 MHz, $CDCl_3$) δ 9.61 (d, *J* = 5.6 Hz, 1H, bpy), 8.03-8.42 (m, 12H, Py*H* and bpy), 7.89 (d, *J* = 9.2 Hz, 1H, bpy), 7.80 (d, *J* = 7.8 Hz, 1H, Py*H*), 7.66 (s, 1H, Im*H*), 7.47-7.57 (m, 2H, bpy), 7.23-7.41 (m, 5H, bpy), 7.08-7.14 (m, 1H, bpy), 6.97 (s, 1H, Im*H*), 6.78-6.86 (m, 2H, bpy), 6.64 (s, 1H, Im*H*), 5.78-5.94 (m, 2H, Py-C*H_2*-N). **^{13}C NMR** (101 MHz, $CDCl_3$) δ 163.1, 161.9, 160.7, 160.4, 151.4, 151.0, 150.7, 150.5, 138.3, 135.2, 134.2, 133.9, 133.7, 132.0, 131.2, 130.6, 129.0, 128.9, 128.3, 127.7, 127.4, 126.9, 126.6, 126.3, 126.2, 126.1, 125.9, 125.1, 125.0, 124.6, 124.5, 123.4, 122.9, 122.8, 121.5, 121.2, 50.3. **HR-MS** (ESI, positive, methanol) (m/z): $[M]^+$ Calcd for $C_{40}H_{30}N_6ClOs$, 821.18353; Found, 821.18388. **FTIR** ῡ = 2906, 1596, 1519, 1457, 1417, 1318, 1254, 1162, 1092, 1038, 1012, 849, 745, 660 cm^{-1}.

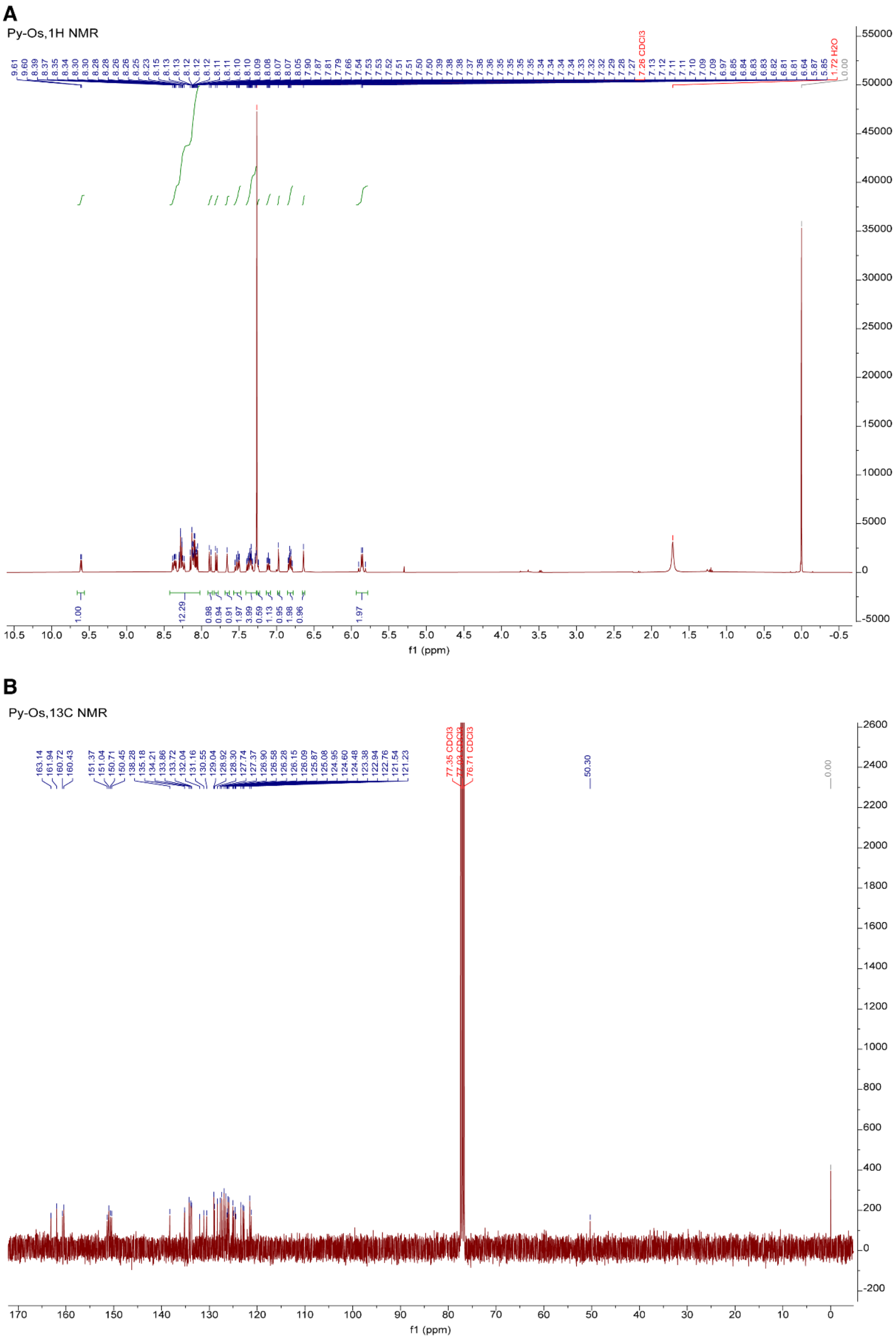

Figure 6. (**A**) ^{1}H NMR and (**B**) ^{13}C NMR spectra of Py-Os in $CDCl_3$.

4.7 Pyrenyl-PEG$_4$-Os (ester) (Py-PEG$_4$-Os (ester), 7)

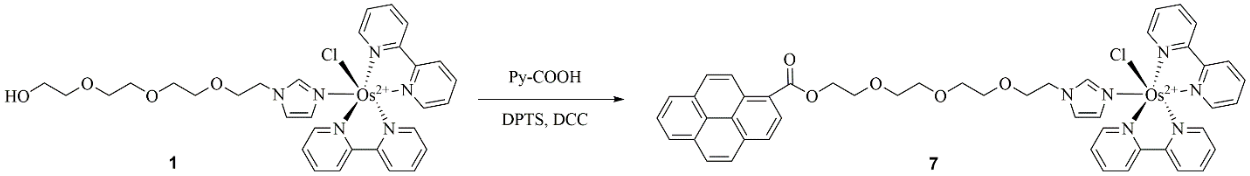

Hy-PEG$_4$-Os (3.9 mg, 5.0 µmol, 1 eq.), Py-COOH (3.7 mg, 15 µmol, 3 eq.), DPTS (0.75 mg, 25 µmol, 0.5 eq.), and DCC (2.0 mg, 10 µmol, 2 eq.) was added to anhydrous DCM (0.5 mL). After stirring for 40 h, the reaction solution was filtrated. Py-PEG$_4$-Os (ester) was purified by column chromatography (gradient, DCM → DCM/MeOH, 9:1 v/v). The silica that could be potentially washed-out during column chromatography was removed by dissolving Py-PEG$_4$-Os (ester) in DCM and filtrating through a thin sand layer. After vacuum drying, Py-PEG$_4$-Os (ester) were obtained as a dark-red solid (4.6 mg, 91%).

Py-PEG$_4$-Os (ester) (Supplementary Fig. 7)

^{1}H NMR (400 MHz, $CDCl_3$) δ 9.77 (d, *J* = 5.6 Hz, 1H, bpy), 9.24 (d, *J* = 9.4 Hz, 1H, Py*H*), 8.57-8.66 (m, 2H, Py*H* and bpy), 8.50 (d, *J* = 8.1 Hz, 1H, bpy), 8.34 (d, *J* = 5.6 Hz, 1H, bpy), 8.04-8.30 (m, 8H, Py*H* and bpy), 7.94 (s, 1H, Im*H*), 7.30-7.64 (m, 8H, bpy), 7.00 (s, 1H, Im*H*), 6.80-6.98 (m, 2H, bpy), 6.47 (s, 1H, Im*H*), 4.65 (t, *J* = 4.8 Hz, 2H, CH_2-N), 3.43-4.11 (m, 14H, PEG backbone). **^{13}C NMR** (101 MHz, $CDCl_3$) δ 167.9, 163.4, 162.1, 161.0, 160.5, 151.5, 151.0, 150.9, 150.4, 138.8, 135.3, 134.4, 134.1, 133.9, 133.8, 131.1, 131.0, 130.4, 129.8, 129.5, 128.5, 127.6, 127.3, 126.9, 126.5, 126.5, 126.4, 126.3, 126.2, 124.9, 124.8, 124.2, 123.6, 123.5, 122.9, 122.7, 121.6, 70.7, 70.6, 70.5, 70.4, 69.7, 69.3, 64.3, 48.1. **HR-MS** (ESI, positive, methanol) (m/z): $[M]^+$ Calcd for $C_{48}H_{44}O_5N_6ClOs$, 1011.26765; Found, 1011.26910. **FTIR** ῡ = 2919, 1706, 1598, 1516, 1457, 1418, 1355, 1323, 1254, 1231, 1197, 1089, 1048, 1013, 949, 852, 753, 720, 660 cm^{-1}.

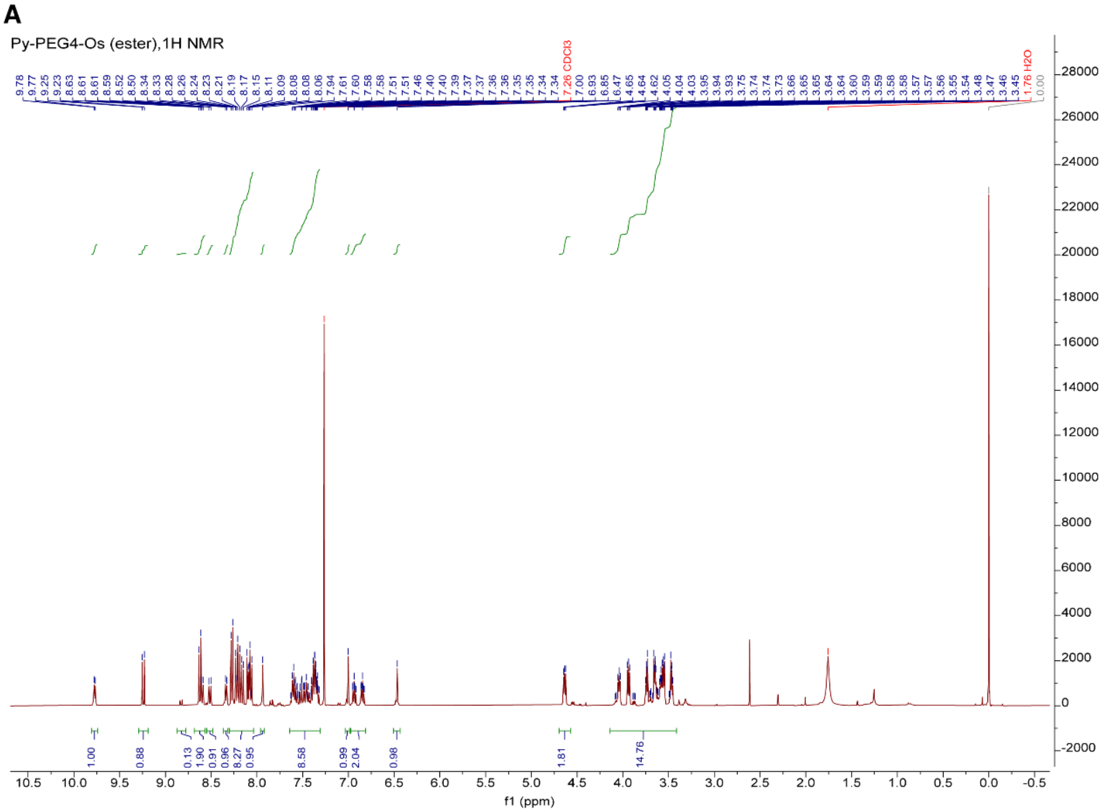

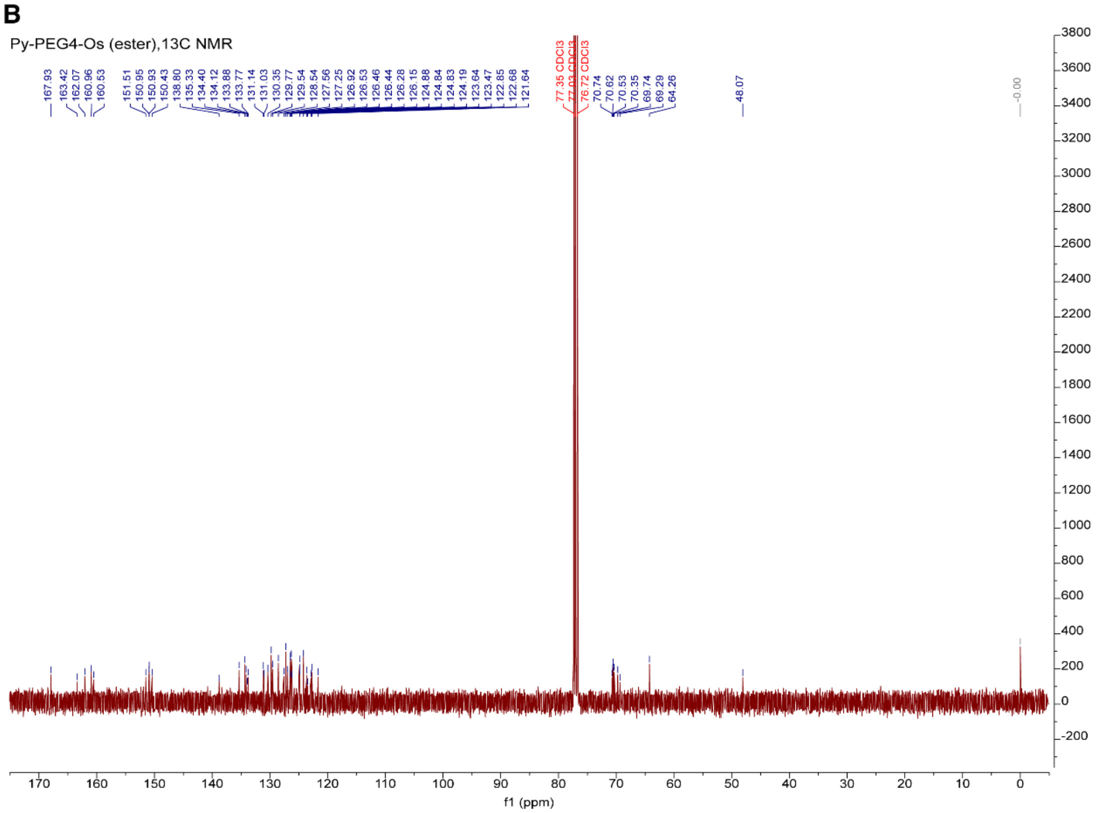

Figure 7. (**A**) ^{1}H NMR and (**B**) ^{13}C NMR spectra of Py-PEG$_4$-Os (ester) in $CDCl_3$.

4.8 Pyrenyl-PEG$_{13}$-Os (ester) (Py-PEG$_{13}$-Os (ester), 8)

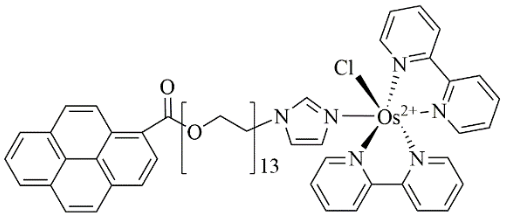

8

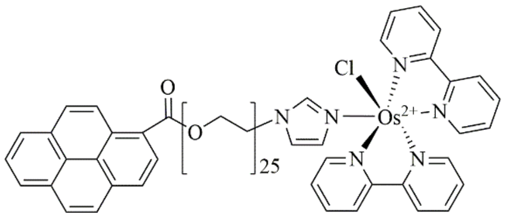

9

Py-PEG$_{13}$-Os (ester) and Py-PEG$_{25}$-Os (ester) were prepared through the same route as Py-PEG$_4$-Os (ester). PEG with a molecular weight of 400 and 1000 Da was used for tosylation. Monotosylated PEG was purified by column chromatography (gradient, AcOEt → acetone), which was confirmed by MALDI-TOF. As-prepared monotosylated PEG was used for the synthesis of Py-PEG$_{13}$-Os (ester) and Py-PEG$_{25}$-Os (ester).

Py-PEG$_{13}$-Os (ester) (Supplementary Fig. 8)

^{1}H NMR (400 MHz, $CDCl_3$) δ 9.80 (d, *J* = 5.6 Hz, 1H, bpy), 9.27 (d, *J* = 9.4 Hz, 1H, Py*H*), 8.63-8.71 (m, 2H, Py*H* and bpy), 8.59 (d, *J* = 8.1 Hz, 1H, bpy), 8.39 (d, *J* = 5.6 Hz, 1H, bpy), 8.03-8.31 (m, 8H, Py*H* and bpy), 7.96 (s, 1H, Im*H*), 7.35-7.70 (m, 8H, bpy), 7.06 (s, 1H, Im*H*), 6.84-7.03 (m, 2H, bpy), 6.54 (s, 1H, Im*H*), 4.66 (t, *J* = 4.8 Hz, 2H, CH_2-N), 3.45-4.20 (m, 50H, PEG backbone). **^{13}C NMR** (101 MHz, $CDCl_3$) δ 168.0, 163.4, 162.1, 161.0, 160.6, 151.5, 151.0, 150.9, 150.5, 138.8, 135.4, 134.4, 134.1, 134.0, 133.8, 131.2, 131.0, 130.4, 129.7, 129.5, 128.6, 127.8, 127.6, 127.2, 127.0, 126.6, 126.4, 126.2, 125.0, 124.2, 123.8, 123.6, 122.9, 122.7, 121.7, 70.8, 70.7, 70.6, 70.5, 70.4, 70.3, 69.8, 69.3, 64.3, 48.1. **FTIR** ῡ = 2866, 1707, 1598, 1516, 1457, 1418, 1348, 1323, 1254, 1234, 1197, 1092, 1045, 1013, 946, 852, 765, 720, 660 cm^{-1}.

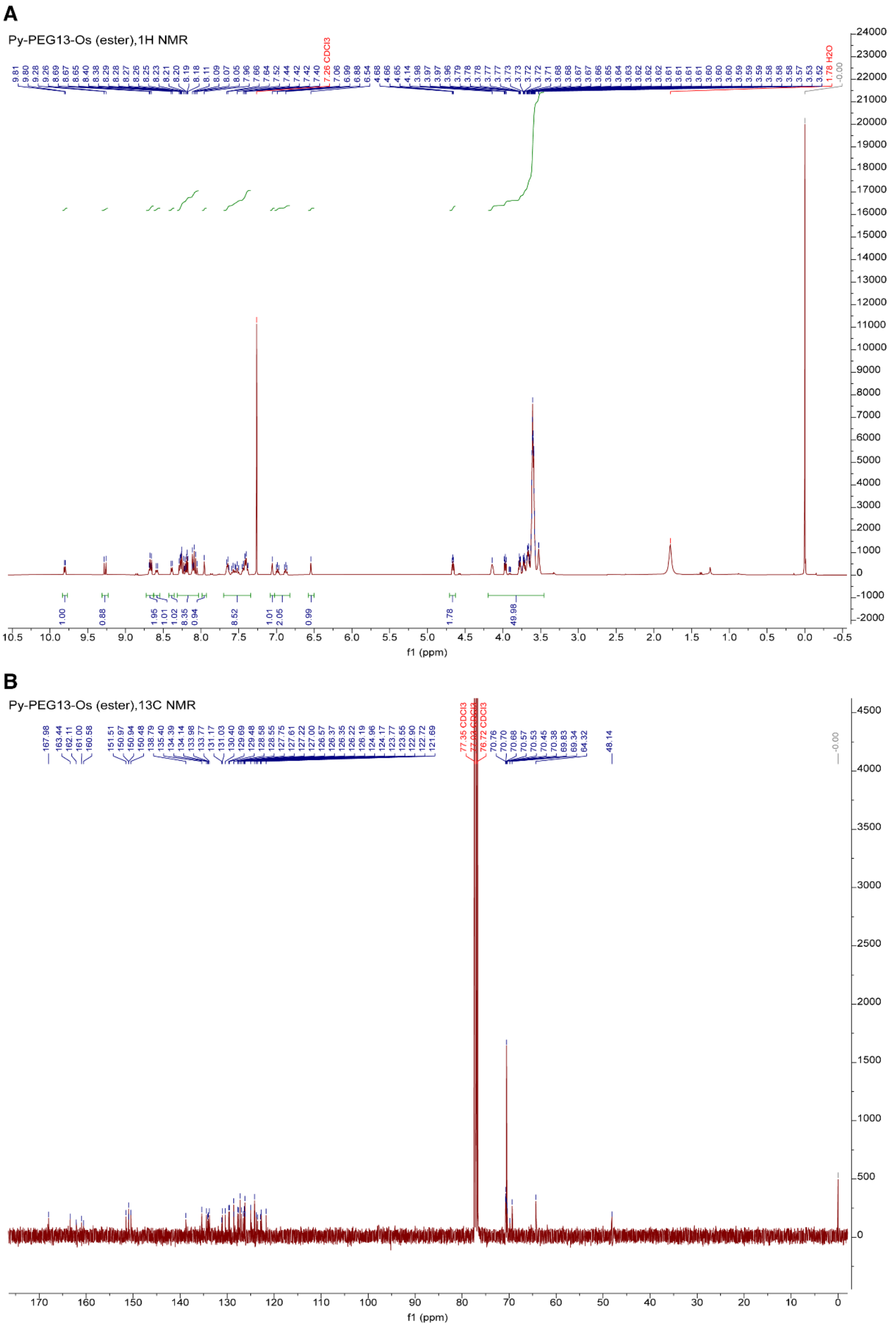

Figure 8a. (**A**) ^{1}H NMR and (**B**) ^{13}C NMR spectra of Py-PEG$_{13}$-Os (ester) in $CDCl_3$.

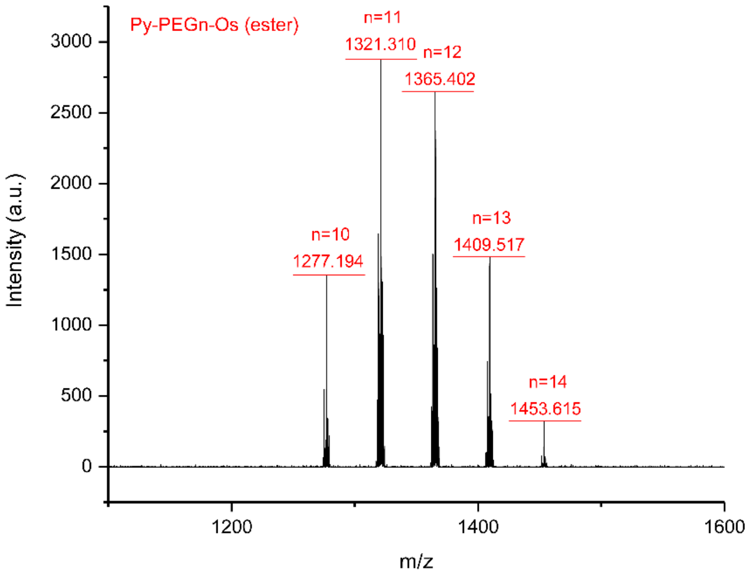

Figure 8b. MALDI-TOF spectrum of Py-PEG$_n$-Os (ester).

4.9 Pyrenyl-PEG$_{25}$-Os (ester) (Py-PEG$_{25}$-Os (ester), 9)

Py-PEG$_{25}$-Os (ester) (Supplementary Fig. 9)

^{1}H NMR (400 MHz, $CDCl_3$) δ 9.81 (d, *J* = 5.6 Hz, 1H, bpy), 9.27 (d, *J* = 9.4 Hz, 1H, Py*H*), 8.67 (d, 2H, Py*H* and bpy), 8.58 (d, *J* = 8.1 Hz, 1H, bpy), 8.40 (d, *J* = 5.6 Hz, 1H, bpy), 8.04-8.31 (m, 8H, Py*H* and bpy), 7.95 (s, 1H, Im*H*), 7.35-7.74 (m, 8H, bpy), 7.06 (s, 1H, Im*H*), 6.84-7.03 (m, 2H, bpy), 6.57 (s, 1H, Im*H*), 4.67 (t, *J* = 4.8 Hz, 2H, CH_2-N), 3.48-4.23 (m, 98H, PEG backbone). **^{13}C NMR** (101 MHz, $CDCl_3$) δ 168.0, 163.5, 162.1, 161.0, 160.6, 151.6, 151.0, 150.5, 138.8, 135.4, 134.4, 134.2, 134.0, 133.9, 131.2, 131.0, 130.4, 129.7, 129.5, 128.6, 128.4, 127.8, 127.6, 127.2, 127.0, 126.6, 126.4, 126.3, 126.2, 126.2, 125.0, 124.9, 124.2, 123.7, 123.6, 122.9, 122.8, 121.7, 70.8, 70.7, 70.5, 70.4, 70.3, 69.8, 69.3, 64.3, 48.1. **FTIR** $\bar{\upsilon}$ = 2866, 1707, 1598, 1526, 1458, 1418, 1350, 1325, 1254, 1236, 1197, 1093, 1038, 1013, 946, 851, 767, 718, 658 cm^{-1}.

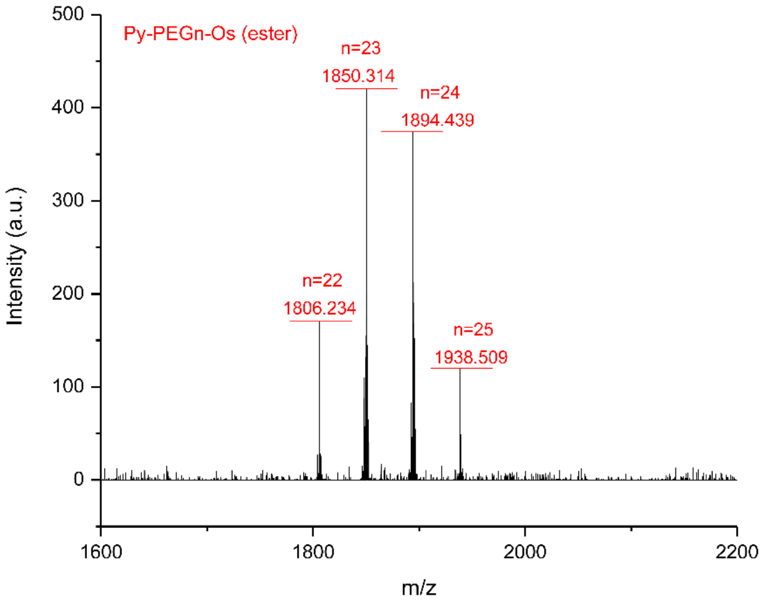

Figure 9a. MALDI-TOF spectrum of Py-PEG$_n$-Os (ester).

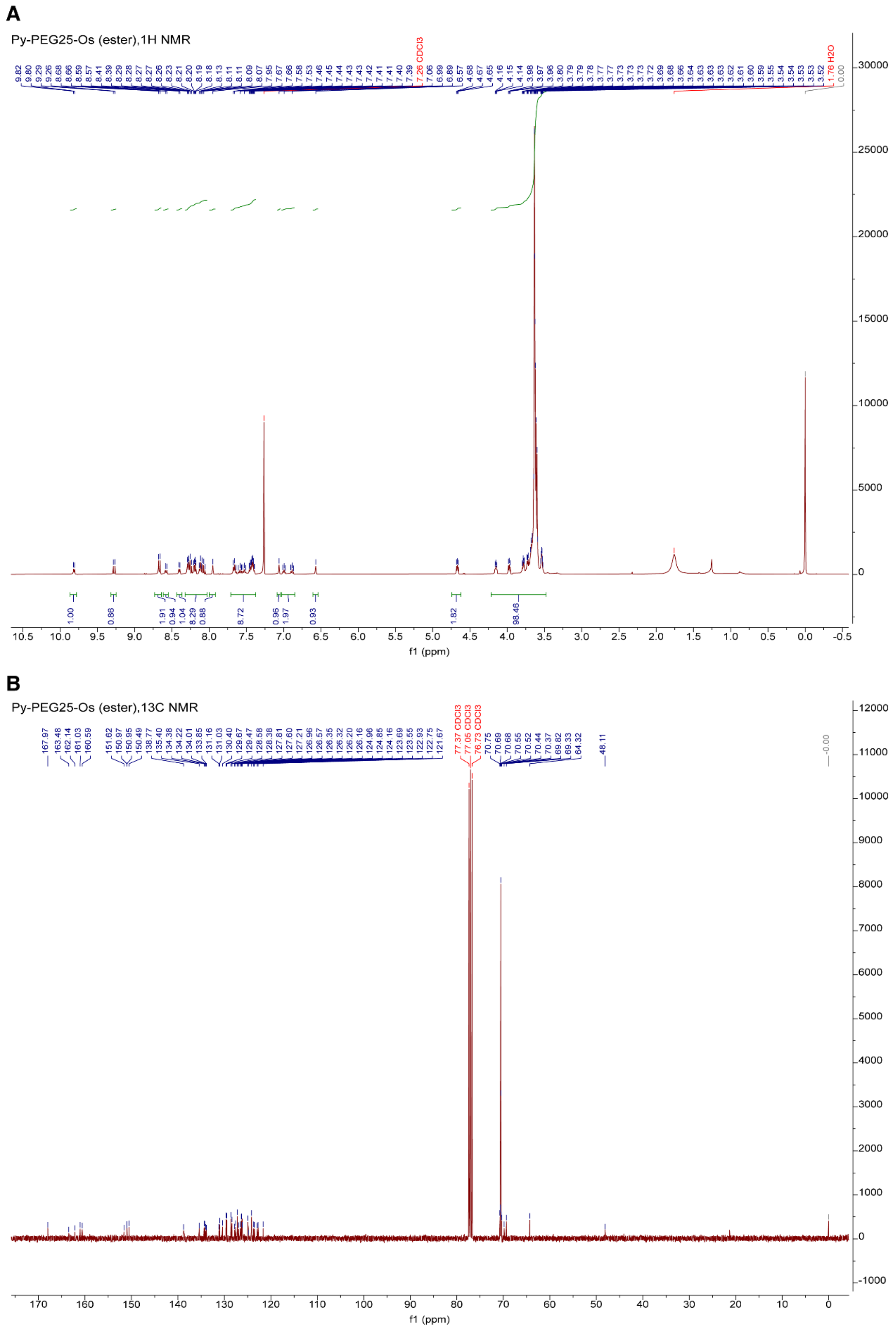

Figure 9b. (**A**) ^{1}H NMR and (**B**) ^{13}C NMR spectra of Py-PEG_{25}-Os (ester) in $CDCl_3$.

5. Conformation of molecular probes dissolved in water

5.1 Critical micelle concentration of molecular probes

Critical micelle concentration (cmc) of Py-PEG$_4$-Im: ~2 mg Py-PEG$_4$-Im was added into the Eppendorf tube with a precise analytical scale, after which 1 mL water was added. The Eppendorf tube was immersed in an ultrasonication bath (VMR ultrasonic cleaner, 200 W) to obtain the homogenous colloid. Different concentration of Py-PEG$_4$-Im was prepared by adding 4-24 μL of the freshly prepared colloid into 600 uL water. After short ultrasonication, the fluorescence of Py-PEG$_4$-Im was measured. I_1/I_3 of the fluorescence was calculated and used for cmc measurements. The point at which I_1/I_3 start to decrease rapidly was assigned to cmc[3]. The concentration of Py-PEG$_4$-Im was measured with a UV-vis spectrometer by diluting the colloid to a concentration lower than cmc.

The cmc of Py-PEG$_4$-Os: To avoid the degradation of the Os by ultrasonication, concentrated Py-PEG$_4$-Os was prepared by dissolving Py-PEG$_4$-Os into a mixture of 50 vol.% methanol and 50 vol.% water. 2-16 uL of the concentrated Py-PEG$_4$-Os was added into 600 uL water for fluorescence measurements. The concentration of Py-PEG$_4$-Os was measured with a UV-vis spectrometer by diluting the colloid to a concentration lower than the cmc.

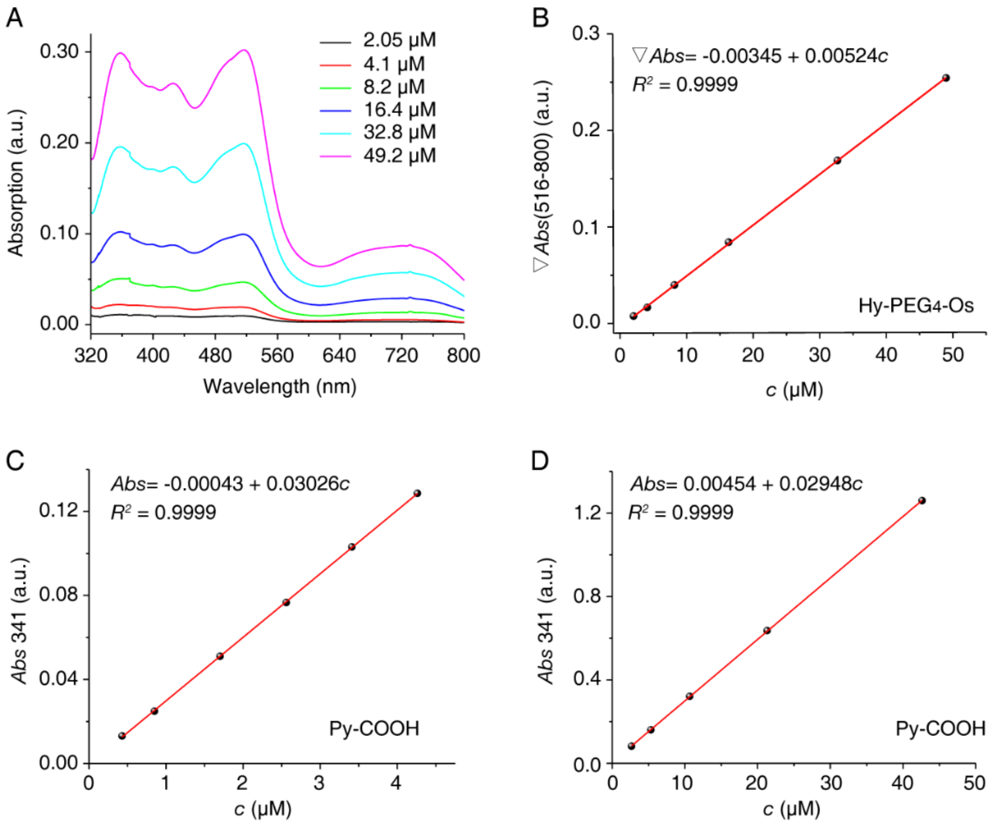

Figure 10. (**A**) UV-vis spectra of different concentrations of Hy-PEG$_4$-Os in water. (**B**) The plot of the absorption difference between 516 nm and 800 nm ($\triangledown$*Abs*(516-800)) of Hy-PEG$_4$-Os in water as a function of concentration. (**C**, **D**) Representative plot of the absorption at 341 nm of Py-COOH in water (pH=7.0) as a function of concentration.

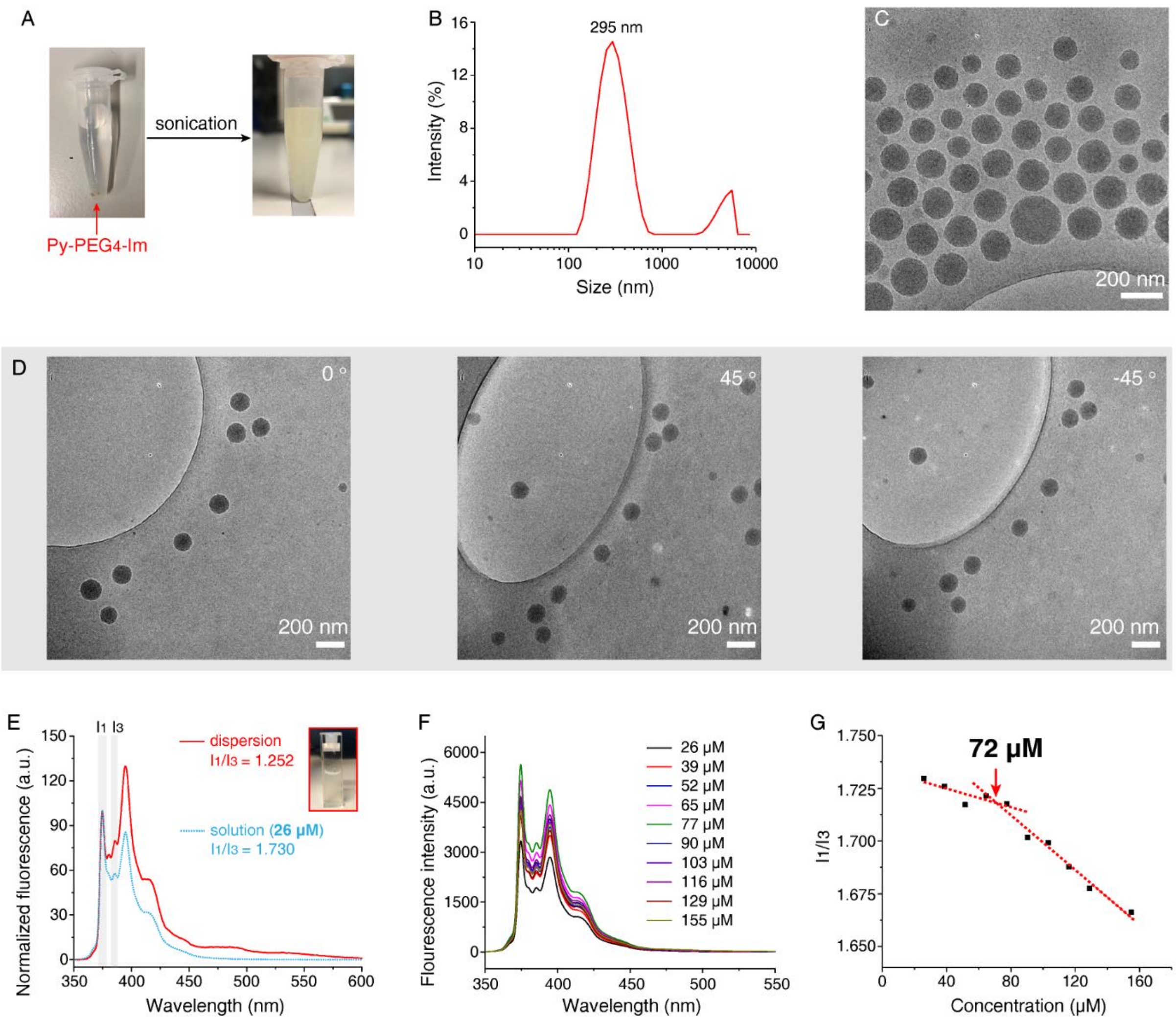

Figure 11. Self-assembly and the cmc of Py-PEG$_4$-Im in water. (**A**) Optical image of Py-PEG$_4$-Im in water before and after ultrasonication. The concentration of Py-PEG$_4$-Im was ~1 mg mL^{-1}. (**B**) DLS intensity size distribution of the aggregates of Py-PEG$_4$-Im. The sample was prepared by diluting the colloid in (A) with water 4 times. (**C**) Cryo-TEM image of the aggregates of Py-PEG$_4$-Im. (**D**) Cryo-TEM images of the aggregates of Py-PEG$_4$-Im at different angles: 0°, 45°, and -45°. (**E**) Normalized fluorescence of the colloid and solution of Py-PEG$_4$-Im. The concentration of Py-PEG$_4$-Im was 26 μM for the solution, and 492 μM for the colloid. (**F**) Fluorescence of different concentrations of Py-PEG$_4$-Im, which was prepared by adding colloid of Py-PEG$_4$-Im (~2 mg mL^{-1}) into water. (**G**) I_1/I_3 of different concentrations of Py-PEG$_4$-Im. The dotted line was generated by the linear fit of Origin 2017.

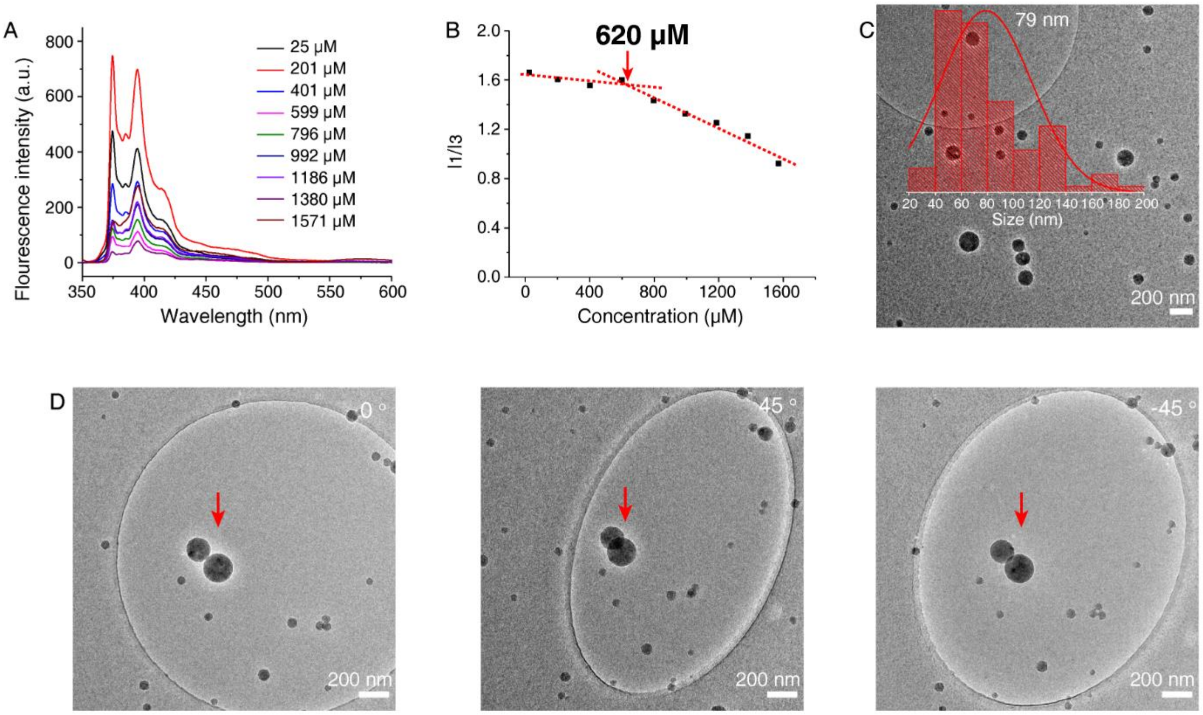

Figure 12. The cmc of Py-PEG$_4$-Os in water. (**A**) Fluorescence of different concentrations of Py-PEG$_4$-Os, which was prepared by adding different volumes of Py-PEG$_4$-Os (60.5 mM) dissolved in the mixture of 50% vol methanol and 50% vol water into water. (**B**) I_1/I_3 of different concentrations of Py-PEG$_4$-Os. The dotted line was generated by the linear fit of Origin 2017. (**C**) Cryo-TEM image of the aggregates in 3 mM Py-PEG$_4$-Os, which was prepared by injecting 32 uL Py-PEG$_4$-Os (60.5 mM) into 600 uL water. The size distribution of the aggregates was obtained by measuring 100 particles with Image J. (**D**) Cryo-TEM images of the aggregates in 3 mM Py-PEG$_4$-Os at different angles: 0°, 45°, and -45°.

5.2 Fluorescence of molecular probes dissolved in water

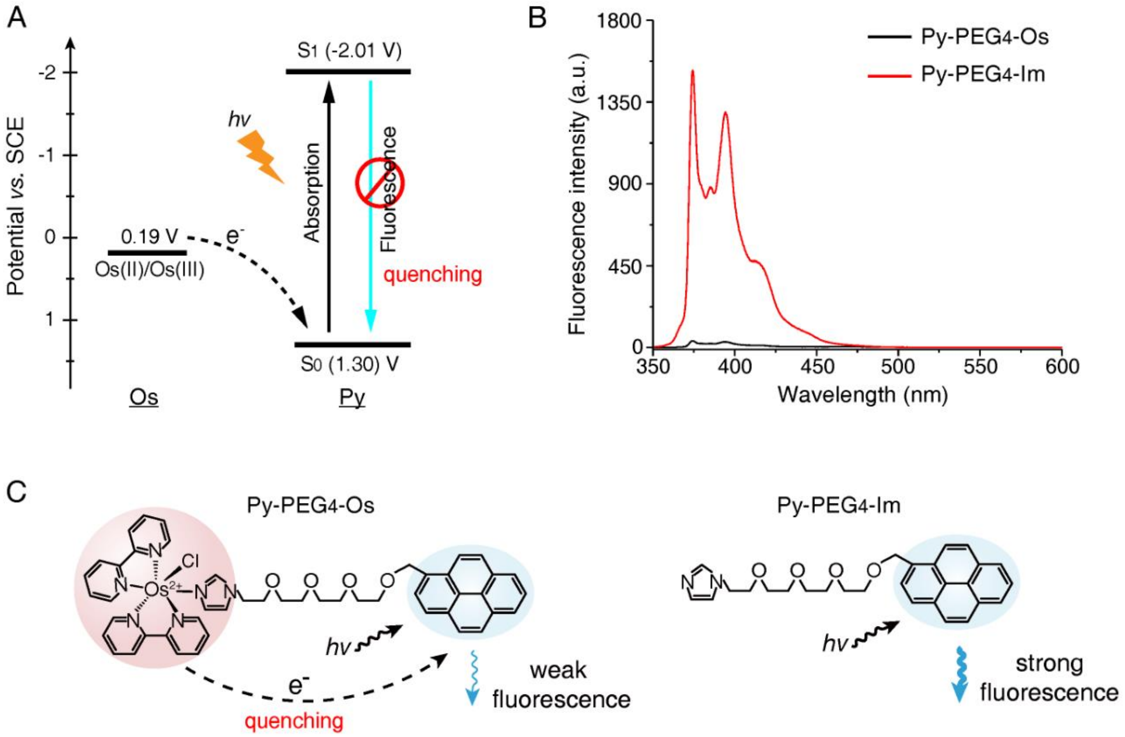

Figure 13. (**A**) Schematic fluorescence quenching of Py by photoinduced electron transfer from Os to Py[4,5]. (**B**) Fluorescence of the aqueous solution of 1.5 μM Py-PEG$_4$-Os and 1.0 μM Py-PEG$_4$-Im. (**C**) Schematic fluorescence of Py-PEG$_4$-Os and Py-PEG$_4$-Im.

5.3 Conformation of molecular probes dissolved in water

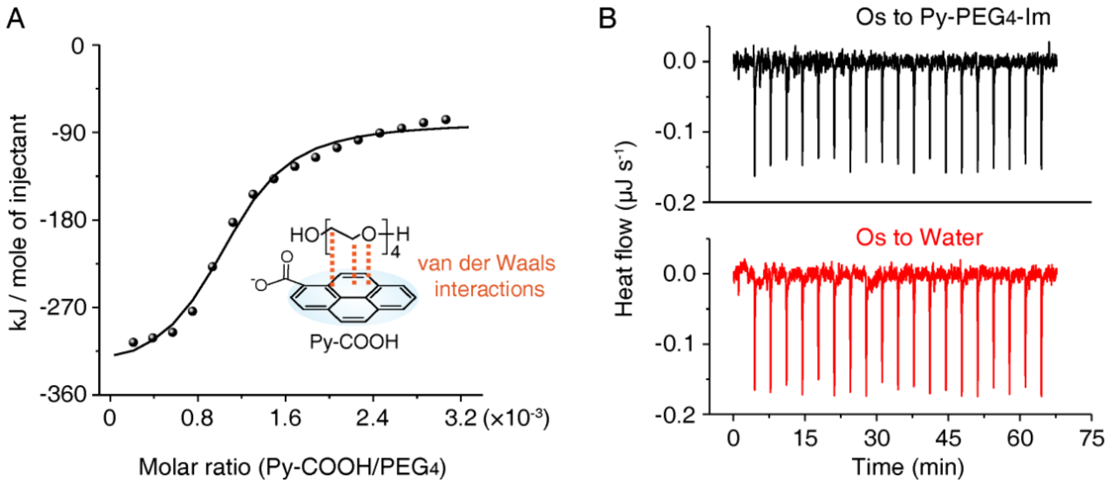

Figure 14. (**A**) ITC analysis of the interaction between Py and PEG_4. 133 μM Py-COOH (aqueous solution, pH=7.0) was injected into 7.5 mM PEG_4 for ITC measurement. (**B**) ITC analysis of the interaction between Py and Os. 397 μM Os (aqueous solution) was injected into a saturated solution of Py-PEG_4-Im for ITC measurement. Similar heat flow suggested a negligible interaction between Os and Py. The potential cation-π interaction between Os and Py should be shielded by the electrically neutral ligand around the Os atom.

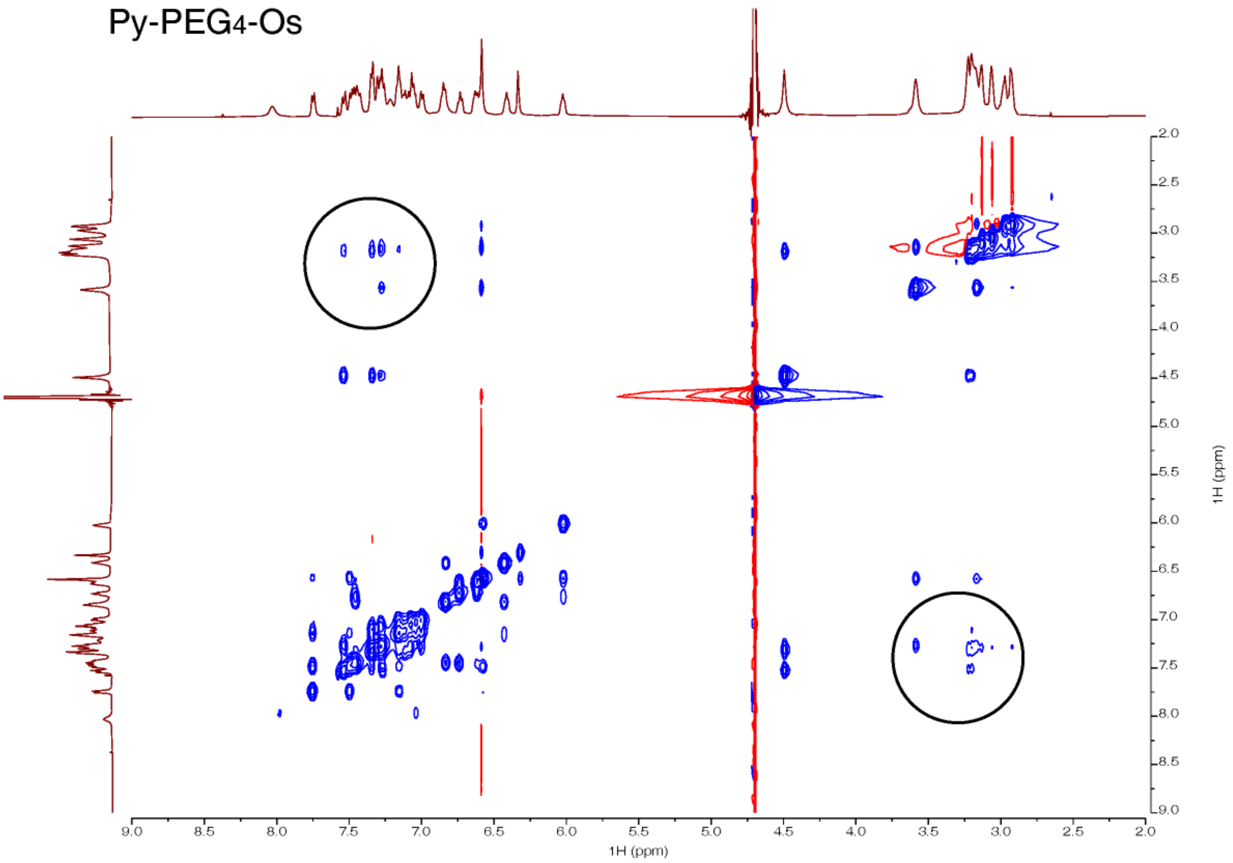

Figure 15. 2D ^{1}H-^{1}H NOESY NMR spectra of Py-PEG$_4$-Os in D_2O. NS = 8, Increments = 256, D1 = 1.5 s, AQ = 0.34 s, mix = 0.5 s. NOE were measured from PEG to imidazole, as they are closely bound (6.59 and 6.66 respectively). NOE was measured from PEG to imidazole, as they are closely bound (6.59 and 6.66 respectively).

For Py-PEG$_4$-Os, clear interaction (black circles) was seen between the signals from PEG$_4$ (3.70-2.83) and Py aromatics (7.60-7.12), proving the proximity between PEG$_4$ spacer and Py (Supplementary Fig. 15). No NOE was observed between the 2,2'-bipyridine (ligands of Os) and PEG$_4$ spacer or Py, suggesting that Os was not interacting with Py. This was consistent with ITC results, *i.e.* no cation-π interaction was detected between Os and Py (Supplementary Fig. 14). The NOESY spectra suggested that Py-PEG$_4$-Os can adopt a folded structure when dissolved in water, where the PEG$_4$ spacer was physically close to Py.

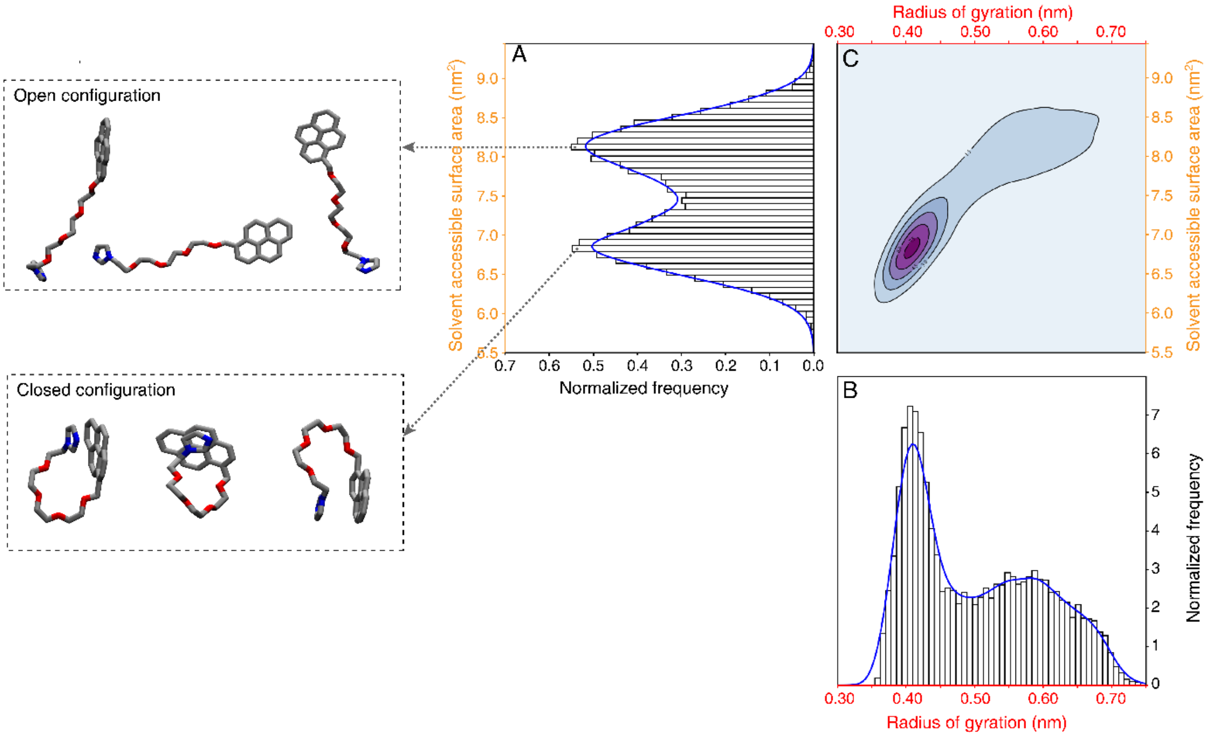

Figure 16. Conformational distribution of Py-PEG_4-Im dissolved in water from molecular dynamics simulation. (**A**) Frequency histograms of solvent accessible surface area for Py-PEG_4-Im. (**B**) Frequency histograms of radius of gyration for Py-PEG_4-Im. The continuous line in (A) and (B) shows the kernel density estimation of probability distribution. (**C**) Two-dimensional kernel density estimation of the probability distribution for Py-PEG_4-Im. Two of the visited conformations of Py-PEG_4-Im are shown on the left side.

Py-PEG_4-Im can adopt a series of conformations when dissolved in water. According to the solvent accessible surface area, two most probable conformations with nearly equal normalized frequency are defined as the closed and open configuration, respectively (Supplementary Fig. 16). For the closed configuration, the PEG_4 spacer is folded, trying to shield Py from water. For the open configuration, the PEG_4 spacer is unfolded, exposing Py to water. According to the two-dimensional kernel density estimation of the probability distribution, the closed configuration has a lower radius of gyration (Rg) than that of the open configuration, which is consistent with the compact structure for closed configuration and extended structure for open configuration (Supplementary Fig. 16).

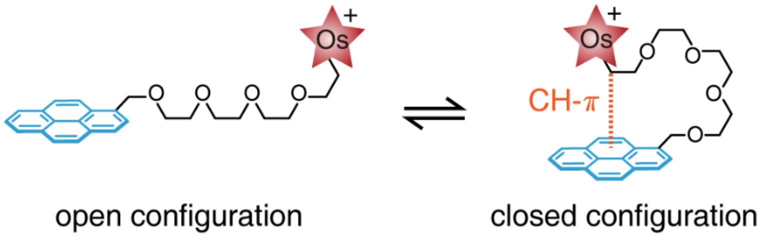

Figure 17. Proposed conformation of Py-PEG4-Os dissolved in water based on 2D ^{1}H-^{1}H NOESY NMR spectra and molecular dynamics simulation. Py-PEG4-Os stays in equilibrium between the open and closed configuration.

6. The loading of molecular probes onto the PEG-modified surface

6.1 The loading of molecular probes onto PEG-*b*-PS polymersomes

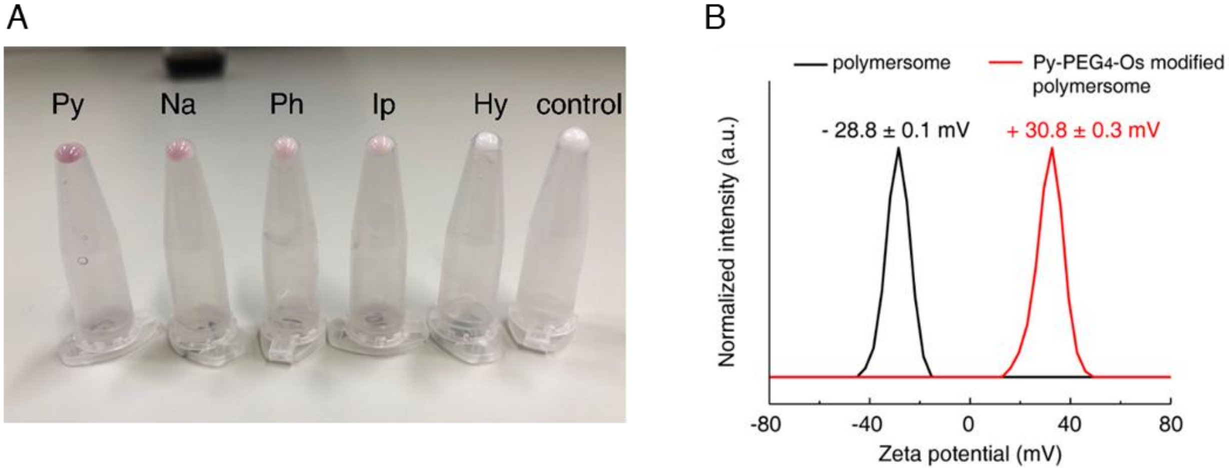

Figure 18. (**A**) Optical image of the precipitated PEG-*b*-PS polymersomes modified by Py-/Na-/Ph-/Ip-/Hy-PEG_4-Os. The control was blank PEG-*b*-PS polymersomes. (**B**) Zeta potential of PEG-*b*-PS polymersomes before and after the modification by Py-PEG_4-Os.

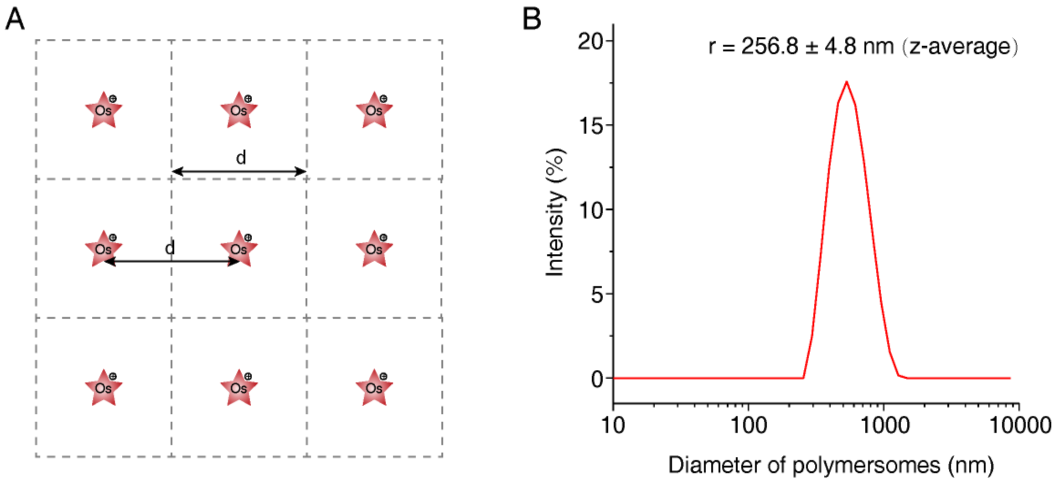

Figure 19. The packing density of Py-PEG4-Os on PEG-*b*-PS polymersomes. (**A**) Schematic distribution of Py-PEG4-Os on the surface of PEG-*b*-PS polymersomes. (**B**) DLS intensity size distribution of PEG-*b*-PS polymersomes dispersed in water. The radius (r) of PEG-*b*-PS polymersomes was 256.8 ± 4.8 nm based on the Z-average size.

The density of Os can be calculated by equation (*S1*):

$$\text{density} = \frac{n}{S} \qquad (S1)$$

where S is the surface area of polymersomes, and n is the number of loaded Os per polymersome.

S was calculated to be 828285 nm^2 with (*S2*):

$$S = 4\pi r^2 \qquad (S2)$$

n was calculated to be 23095 or 25000 with (*S3*):

$$n = \sqrt{\frac{\text{amount of loaded } molecular \text{ probes}}{number\ of\ polymersomes}} \qquad (S3)$$

where the *amount of loaded molecular probes* was measured to be 9.7 or 10.5 nmol with UV-vis spectrometer, the *number of polymersoms* was measured to be 4.2×10^{-4} nmol with NanoSight LM10.

Thus, the density of Os was calculated to be **0.029 ± 0.002 Os/nm^2**.

Here, the separation distance between the adjacent Os was defined as d. The area occupied by each Os on the surface of the PEG-*b*-PS polymersomes was defined as a square (d × d). d was calculated to be **5.9±0.2 nm** by equation (*S4*):

$$d = \sqrt{\frac{S}{n}} \qquad (S4)$$

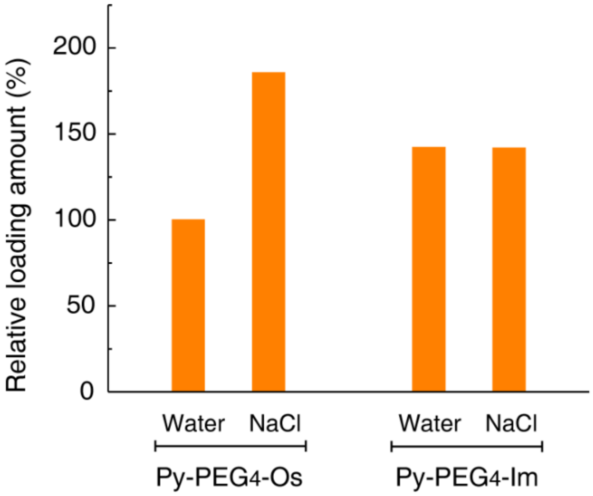

Figure 20. The relative loading amount of Py-PEG4-Os and Py-PEG4-Im on PEG-*b*-PS polymersomes in water or 20 mM NaCl. Each value corresponds to mean of n = 2 replicates.

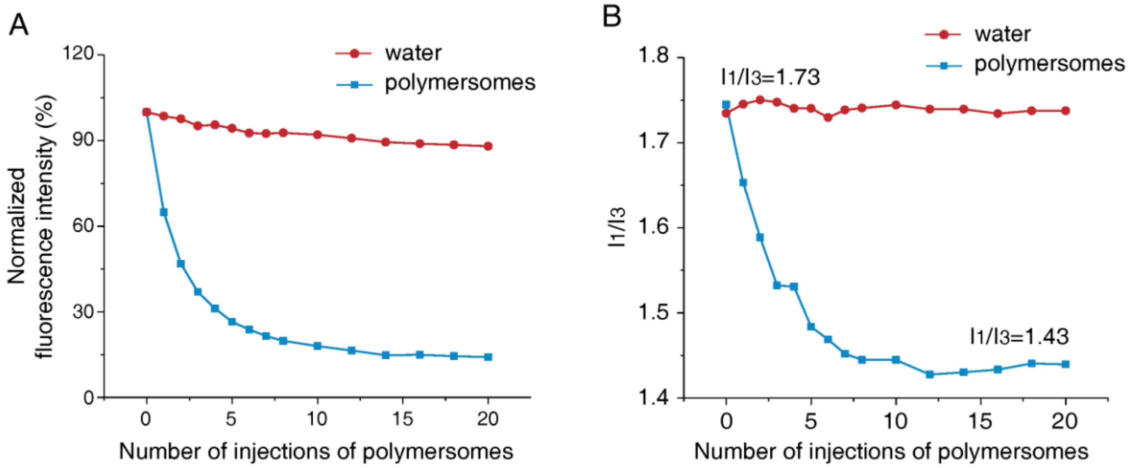

Figure 21. Change of (**A**) fluorescence intensity and (**B**) I_1/I_3 during the loading of Py-PEG$_4$-Im onto PEG-*b*-PS polymersomes. 100 uL dispersion of PEG-*b*-PS polymersomes ($5.6\times10^{-9} \pm 2.2\times10^{-10}$ mol L^{-1}) or water was injected into Py-PEG$_4$-Im (4.5 μM, 1.2 mL) in 20 times.

6.2 Distribution of molecular probe on polymersomes

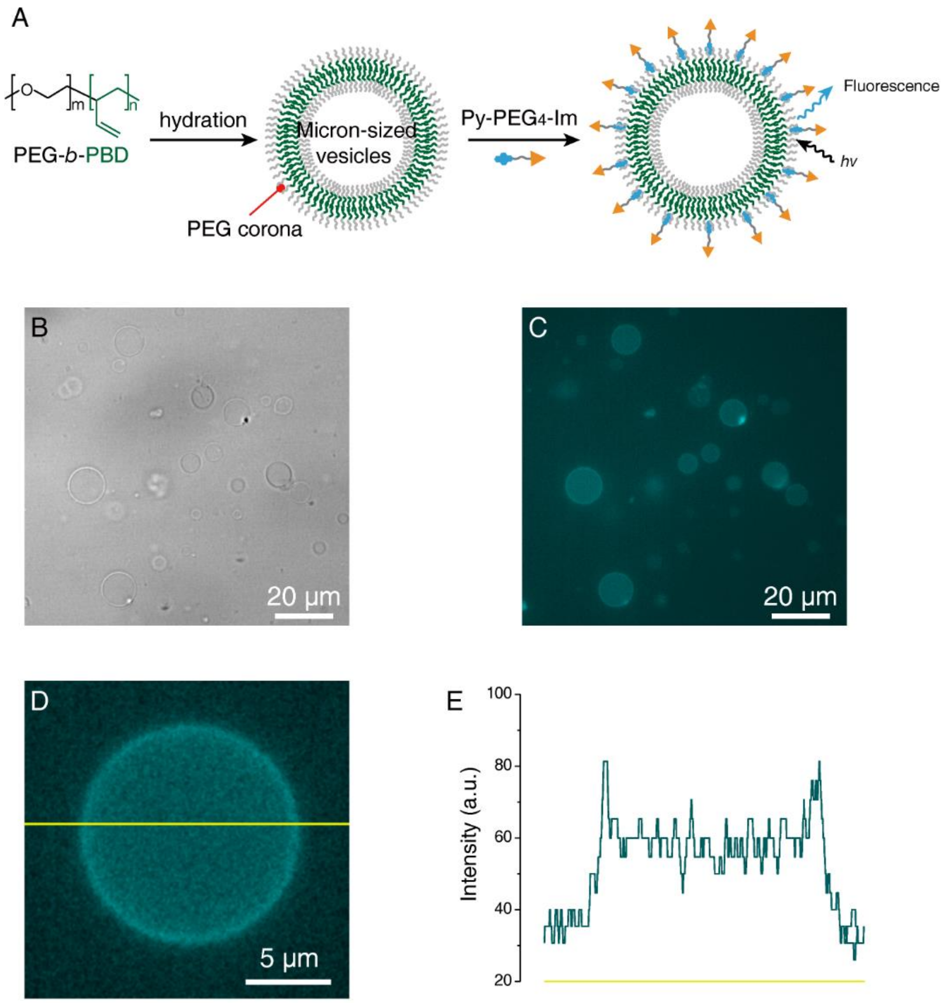

Figure 22. (**A**) Schematic preparation of micron-sized polymersomes. (**B**) Brightfield, and (**C**) fluorescence image of micron-sized polymersomes loaded with Py-PEG$_4$-Im. (**D**) Fluorescence image of a single micron-sized polymersome loaded with Py-PEG$_4$-Im. (**E**) Fluorescence intensity along the yellow line of (D). The plot was generated by Image J.

Micron-sized polymersome with PEG corona was prepared with poly(ethylene oxide)-*b*-poly(1,2-butadiene) (PEG-*b*-PBD) through hydration method[6]. Specifically, 1.25 mg PEG-*b*-PBD was dissolved in chloroform, which was dried by the stream of nitrogen. After overnight vacuum desiccation, 3 mL H_2O was added and the mixture was incubated in a 60 ^oC water bath for 24 h. The final volume was adjusted to be 3 mL. As-prepared micron-sized polymersomes were incubated with Py-PEG$_4$-Im in a chamber slide (300 μL). The concentration of Py-PEG$_4$-Im was

33 µM in the incubation solution. After incubating for at least 10 min, the micron-sized polymersomes were characterized by fluorescence microscopy.

6.3 The loading of molecular probes onto PEG and PS microparticles

6.3.1 Preparation of PEG microparticles

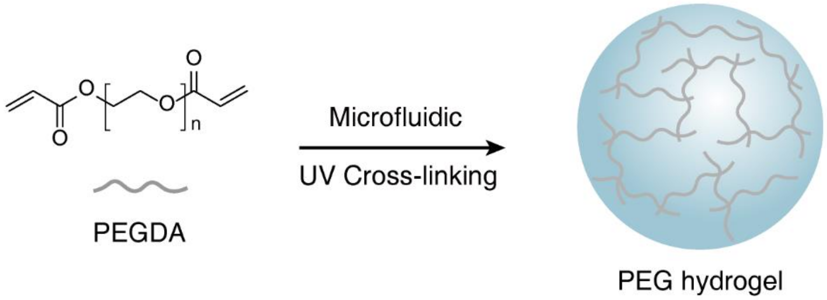

PEG microparticles were prepared by a microfluidic setup as reported in our previous publication[7]. All of the solutions were flushed with nitrogen for 0.5 h to remove dissolved oxygen. The fluorocarbon oil (HFE 7500) and the PEGDA (40% w/w) were injected respectively in the first and second inlet of the PDMS microfluidic device. Irgacure 2959 (0.4 wt% final concentration) was added to the PEGDA solution before injection. The droplets were formed at the cross-junction by the introduction of an outer phase consisting of fluorocarbon oil (HFE 7500) and surfactant (SS01, 1% w/w). The resulting emulsion was collected in an Eppendorf and then UV cured by exposure to a focused UV beam (λ = 320-500 nm, 5 min, 60% intensity). The emulsion was broken by adding 1H,1H,2H,2H-Perfluoro-1-octanol (100µL, 20% w/w in hexane), after which the microparticles were washed with Milli-Q. Flow rates were 600 µL h^{-1} for fluorocarbon oil, and 60 µL h^{-1} for PEGDA. PEG microparticles were dispersed in 0.5 mL of water. When used for the loading of Py-PEG_4-Im, the dispersion of PEG microparticles was diluted 200 times with water.

6.3.2 Preparation of PS microparticles

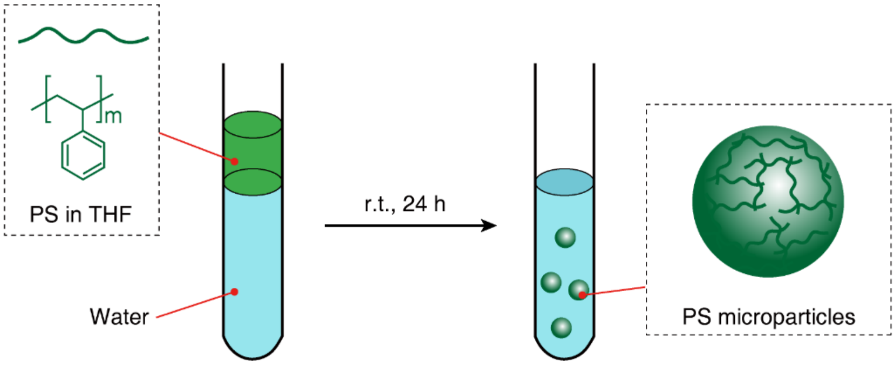

PS microparticles were prepared according to previously reported procedures[8]. Commercial polystyrene (PS, M_w 20kDa, PDI = 1.02) was used for the preparation. 2 mg PS was dissolved in 2 mL THF. 200 uL PS solution was gently poured onto 1 mL water. After incubating for 24 h at room temperature, PS microspheres were obtained as a dispersion in water. The residual THF was removed by rotary evaporation. The final volume of the PS dispersion was adjusted to 1 mL by adding water. This dispersion was directly used for the loading of Py-PEG_4-Im.

6.3.3 The loading of Py-PEG_4-Im onto PEG and PS microparticles

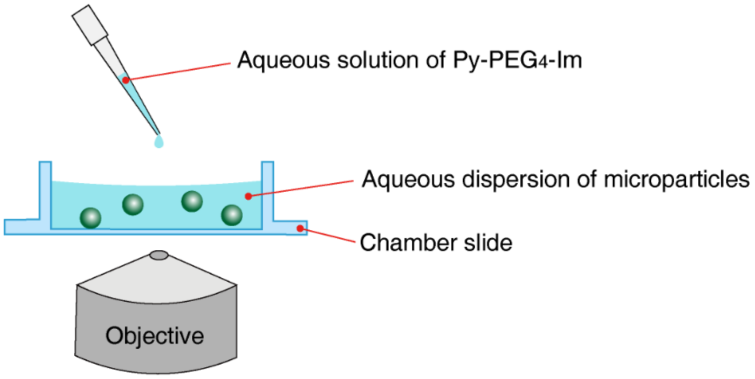

We built an experimental device to visualize the loading of molecular probes. PS or PEG microparticles was incubated with Py-PEG_4-Im in the chamber slide. The concentration of Py-PEG_4-Im was 33 μM in the incubation solution. After incubating for at least 10 min, PS or PEG microparticles were visualized by a fluorescence microscope.

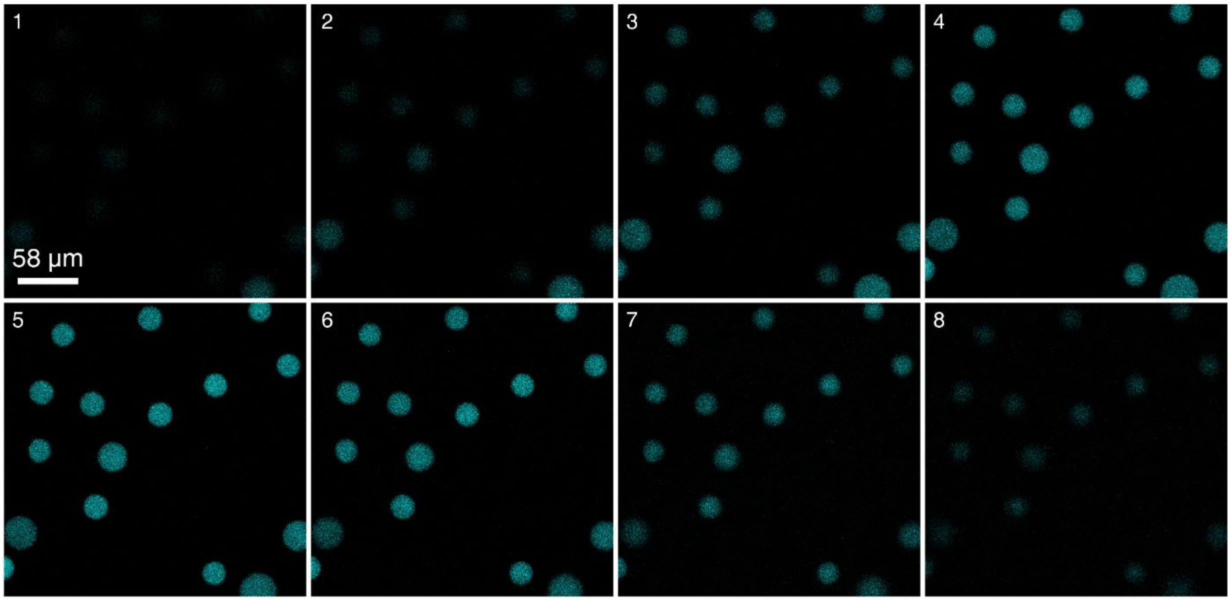

Figure 23. Confocal z-stack images of Py-PEG_4-Im modified PEG microparticles. The image was taken every 4 μm along the z-axis.

6.4 Loading mechanism by molecular dynamics simulation

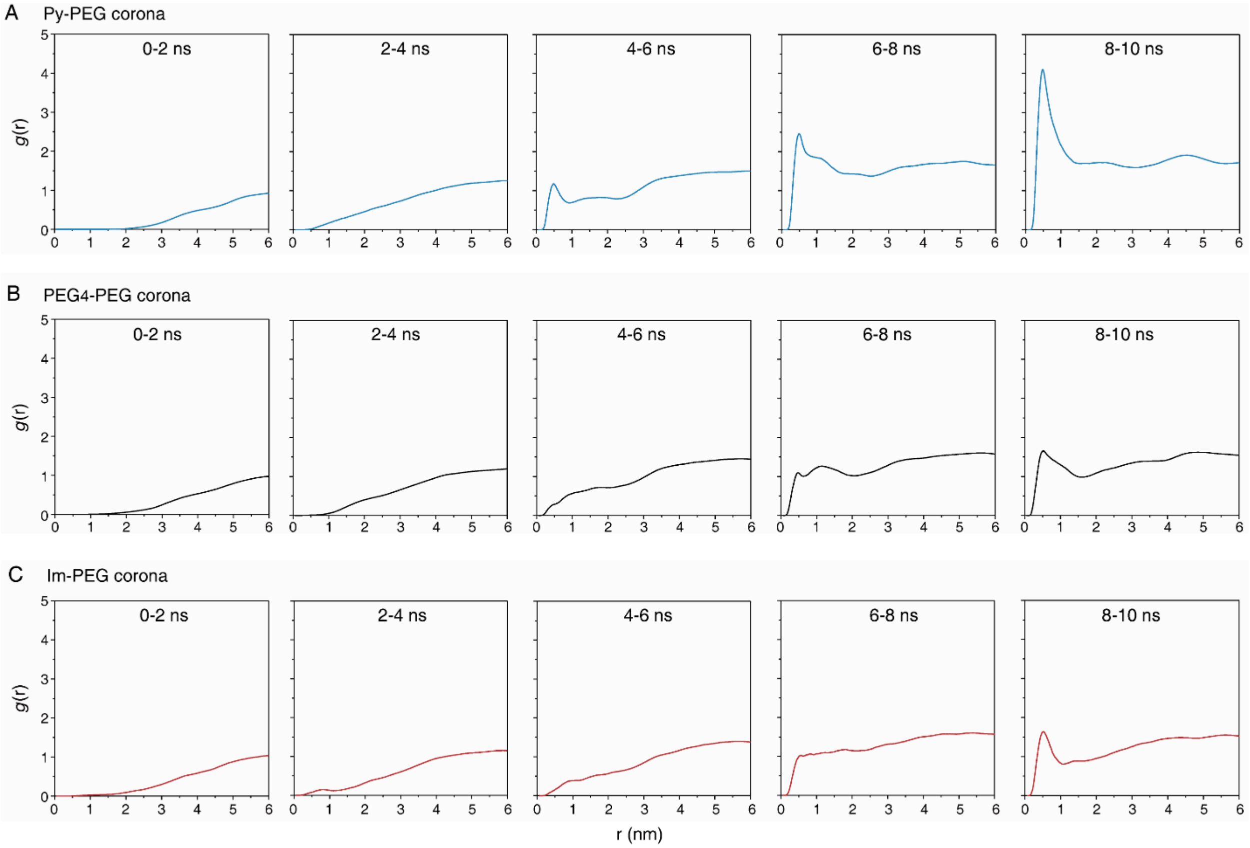

Figure 24. Radial distribution function (RDF) of (**A**) Py-PEG corona, (**B**) PEG_4-PEG corona, and (**C**) Im-PEG corona at 0-2, 2-4, 4-6, 6-8, and 8-10 ns. At 0-4 ns, Py was far away from PEG corona. At 4-6 ns, a peak at ~0.49 nm appeared in the RDF for Py and became much larger in the next 4 ns, demonstrating that Py is approaching PEG corona. At 8-10 ns, the peak height in the RDF for Py was much higher than that for PEG_4 and Im. This suggested the stronger interaction between Py and PEG corona, confirming the role of Py as the anchor of PEG corona.

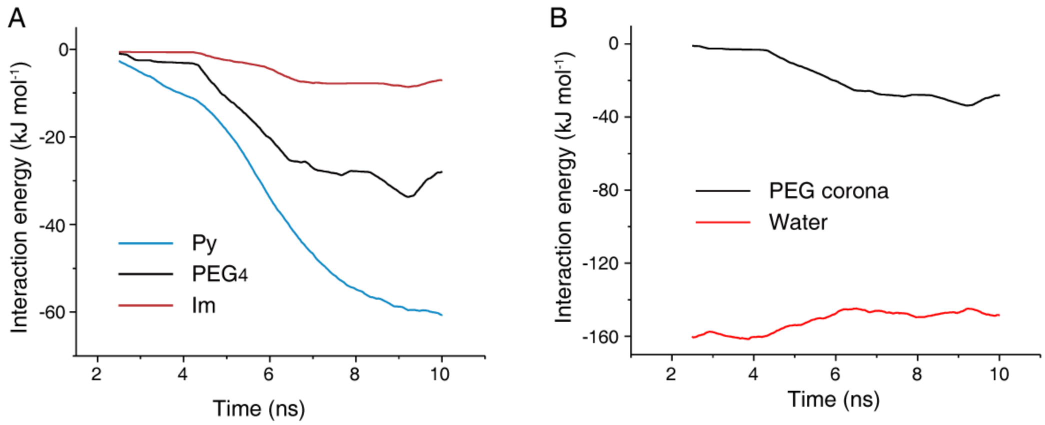

Figure 25. (**A**) The interaction energy between different segments of the molecular probe and PEG corona. (**B**) The interaction energy between PEG_4 of the molecular probe, and PEG corona or water.

6.5 Interaction between molecular probes and PEG-*b*-PS polymersomes

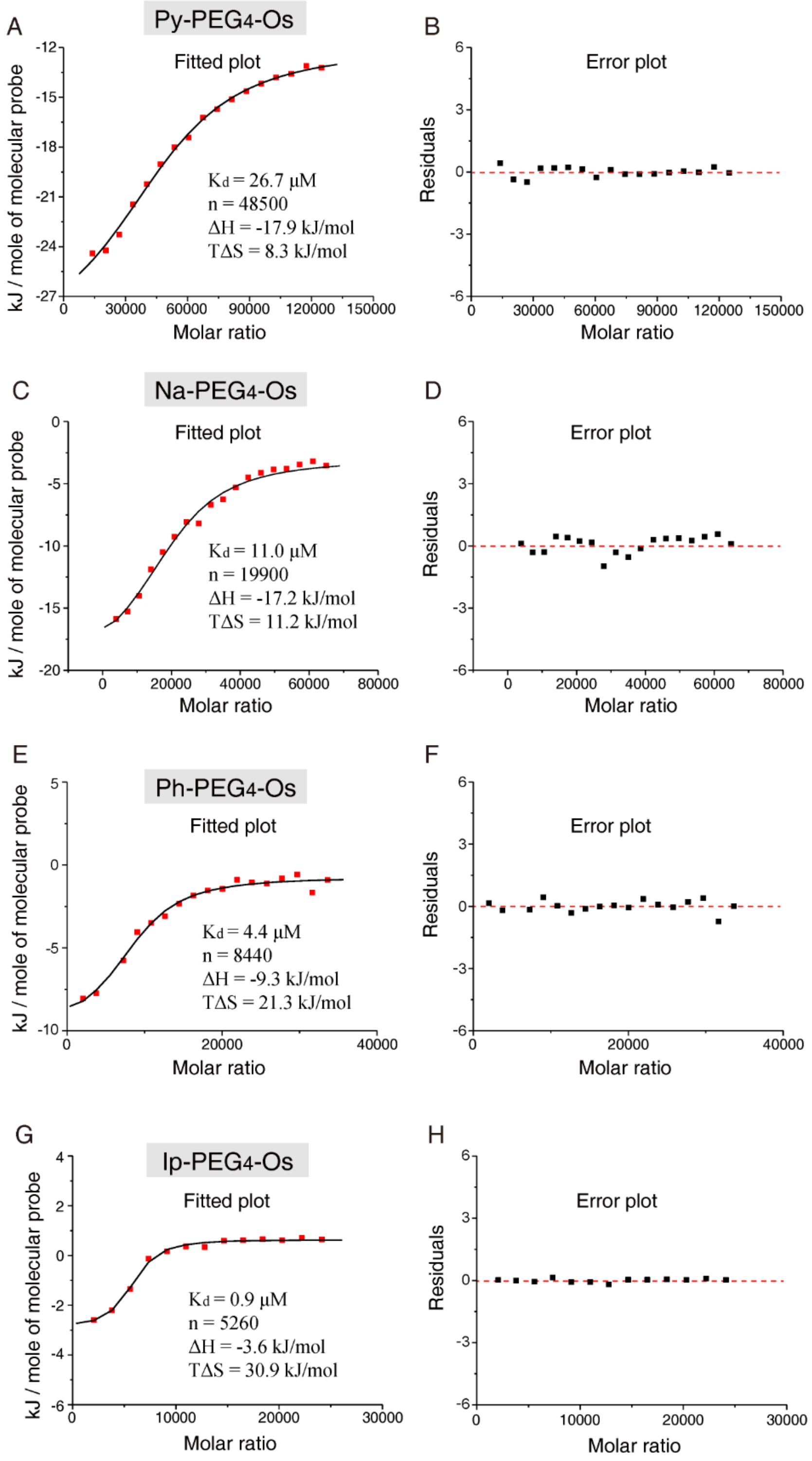

Figure 26. (**A**, **C**, **E**, **G**) Fitted plots and (**B**, **D**, **F**, **H**) error plots of ITC data from the titration of molecular probes into polymersomes. The plots were generated by MicroCal PEAQ-ITC Analysis Software.

Table 1. Thermodynamic parameters for the binding of molecular probes and PEG-*b*-PS polymersomes by ITC

Parameter	Ip	Ph	Na	Py
K_d (μM) [a]	0.9	4.4	11	26.7
n [b]	5260	8440	19900	48500
ΔH (kJ/mol) [c]	-3.6	-9.3	-17.2	-17.9
ΔG (kJ/mol) [d]	-34.5	-30.6	-28.3	-26.1
TΔS (kJ/mol) [e]	30.9	21.3	11.2	8.3

[a] K_d, Dissociation constant

[b] n, Binding stoichiometry

[c] ΔH, Enthalpy change

[d] ΔG, Gibbs free energy change

[e] ΔS, Entropy change

It should be noted that ΔG and ΔS are not comparable for different molecular probes due to their dramatic different binding stoichiometry. However, the negative ΔG demonstrates that the insertion of the hydrophobic anchor into the PEG corona is thermodynamically favorable.

Table 2. Sensitivity test by setting n as different values

	n	100	1,000	500,000	Optimized
Ip-PEG$_4$-Os	TΔS (kJ/mol)	**-242**	2.5	**-65.4**	30.9 (n=5250)
Ph-PEG$_4$-Os	TΔS (kJ/mol)	-1030	**-76.6**	**-205**	21.3 (n=8440)
Na-PEG$_4$-Os	TΔS (kJ/mol)	—	**-451**	**-787**	11.2 (n=19900)
Py-PEG$_4$-Os	TΔS (kJ/mol)	—	**-1230**	**-39.3**	8.3 (n=48500)

To confirm the positive ΔS, a sensitivity test was performed by setting n as the significantly different values from the optimized one. As shown in Supplementary Table 2, negative ΔS were obtained at high or low n (grey shade). Thus, the positive ΔS at optimized n was attributed to the loading of molecular probes onto polymersomes.

$$Py + PEG\ corona \cdot mH_2O \longrightarrow [Py \cdot PEG\ corona] \cdot (m\text{-}x)H_2O + xH_2O$$

enthalpy change ($\triangle H < 0$)
(i) $\triangle$**H**$<$**0: The formation of non-covalent interaction between Py and PEG corona**;
(ii) $\triangle H > 0$: The disruption of the well-defined aqueous shell of PEG corona.

entropy change ($\triangle S > 0$)
(i) $\triangle S < 0$: The conformational restriction of molecualr probe upon loading;
(ii) $\triangle S > 0$: **The release of water from PEG corona upon loading**.

Figure 27. Simplified binding equation of Py and PEG corona, and the factors that determined the enthalpy change (ΔH) and entropy change (ΔS) of the binding process. According to previous results, no aqueous shell was detected around Py[9]. Thus, the binding equation of Py and PEG corona was simplified as Supplementary Fig. 27.

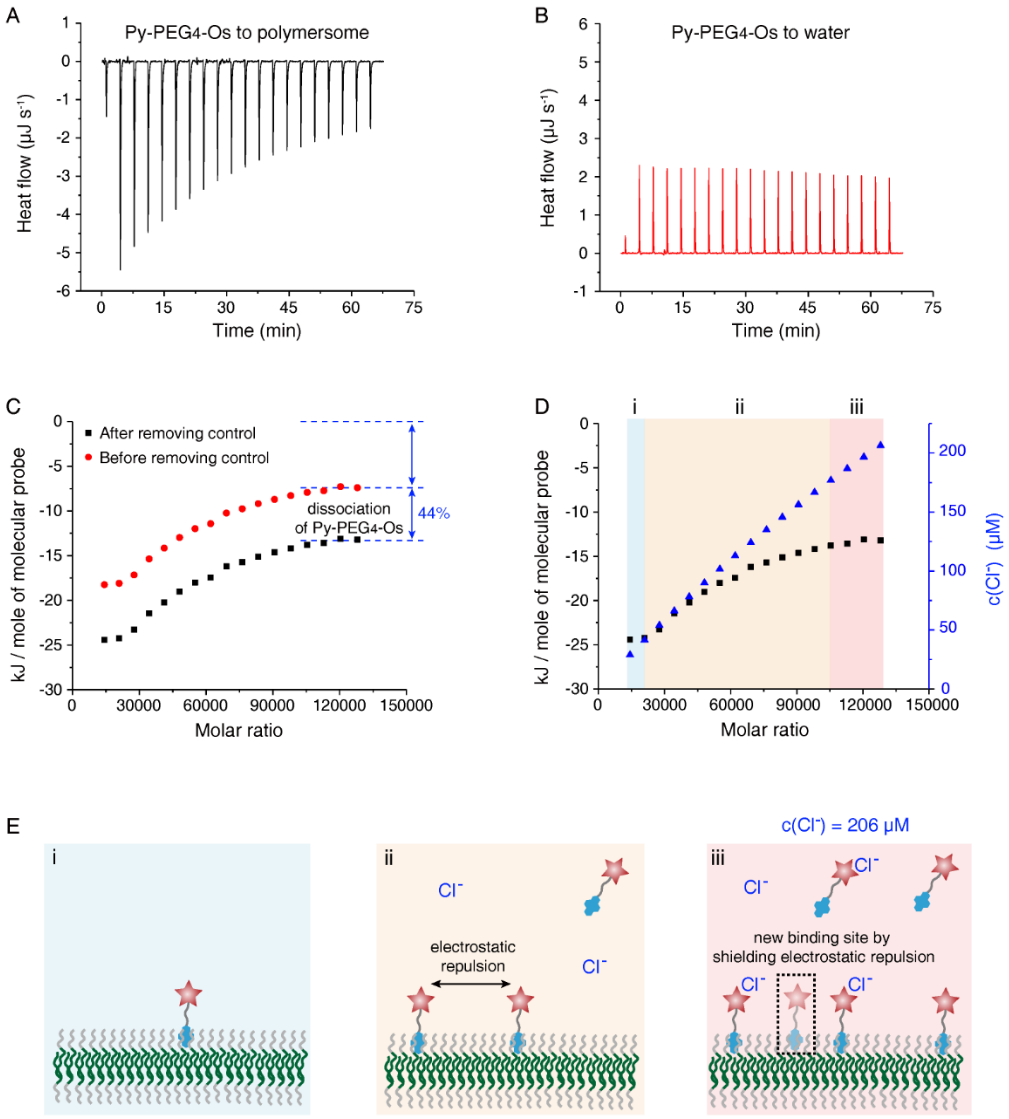

Figure 28. Raw data plot of heat flow over time for the titration of 1.34 mM Py-PEG4-Os into (**A**) dispersion of polymersome and (**B**) water (control). The endothermic peaks in (B) should be caused by the dissociation of the aggregates of Py-PEG4-Os formed at a concentration higher than cmc. (**C**) ITC isotherm obtained by integrating the heat flow peaks. The heat of dissociation deviated the ITC isotherm from zero at the end of the titration. (**D**) The integrated heat and concentration of chloride anion (Cl^-) in the sample cell during the titration of Py-PEG4-Os. The binding between Py-PEG4-Os and polymersome was divided into three stages in ITC. (**E**) Schematic loading of Py-PEG4-Os onto polymersome at different stages. At the i stage, most of the added Py-PEG4-Os were loaded onto the polymersome, generating the most significant heat flow. At the ii stage, part of the added Py-PEG4-Os was loaded onto the polymersome. At the iii stage, the accumulated Cl^- in the sample cell would shield the electrostatic repulsion between

loaded Os, creating new binding sites for the excess molecular probes, which would also deviate ITC isotherm from zero at the end of the titration.

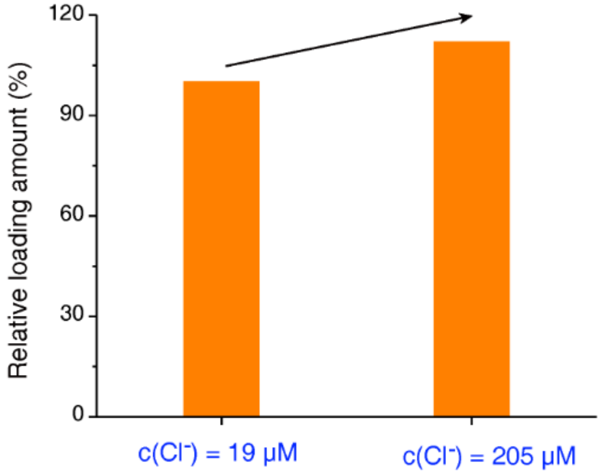

Figure 29. The loading of Py-PEG$_4$-Os onto polymersome under 19 μM and 205 μM of Cl^-. The concentration of Py-PEG$_4$-Os was 19 μM. For 19 μM, the Cl^- was the counter ion of Py-PEG$_4$-Os. For 205 μM, 186 μM Cl^- was introduced by adding NaCl. As compared with the loading under normal conditions (19 μM Py-PEG$_4$-Os), 205 μM of Cl^- increased the loading amount of Py-PEG$_4$-Os by ~10%. This demonstrated that the accumulated Cl^- during ITC can create new binding sites for Py-PEG$_4$-Os, deviating ITC isotherm from zero at the end of the titration.

6.6 The loading of molecular probes onto PEG-*b*-PDLLA polymersomes

6.6.1 Preparation of PEG-*b*-PDLLA polymersomes

PEG-*b*-PDLLA polymersomes were prepared according to our previous study (Supplementary Fig. 30)[10]. 5 mg PEG_{22}-$PDLLA_{90}$ and 5 mg PEG_{44}-$PDLLA_{86}$ were dissolved in 1 mL of a mixture of THF and 1,4-dioxane (THF: 1,4-dioxane = 1:4 by volume). 1 mL water was injected into the above mixture at 1 mL h^{-1}. 10 mL of water was added. After centrifugation and water wash (3 times), PEG-*b*-PDLLA polymersomes were dispersed in water.

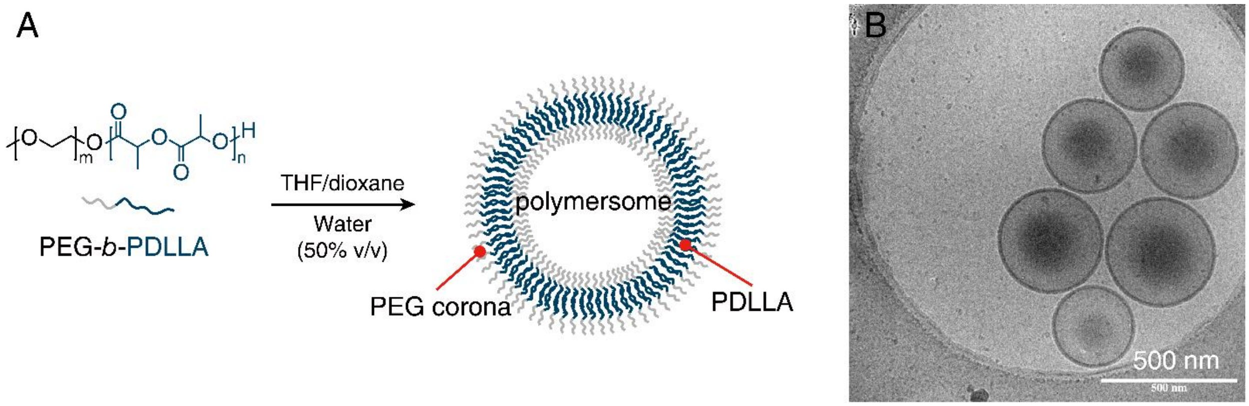

Figure 30. (**A**) Schematic preparation of PEG-*b*-PDLLA polymersome. (**B**) Cryo-TEM image of PEG-*b*-PDLLA polymersome.

6.6.2 The loading of molecular probes onto PEG-*b*-PDLLA polymersomes

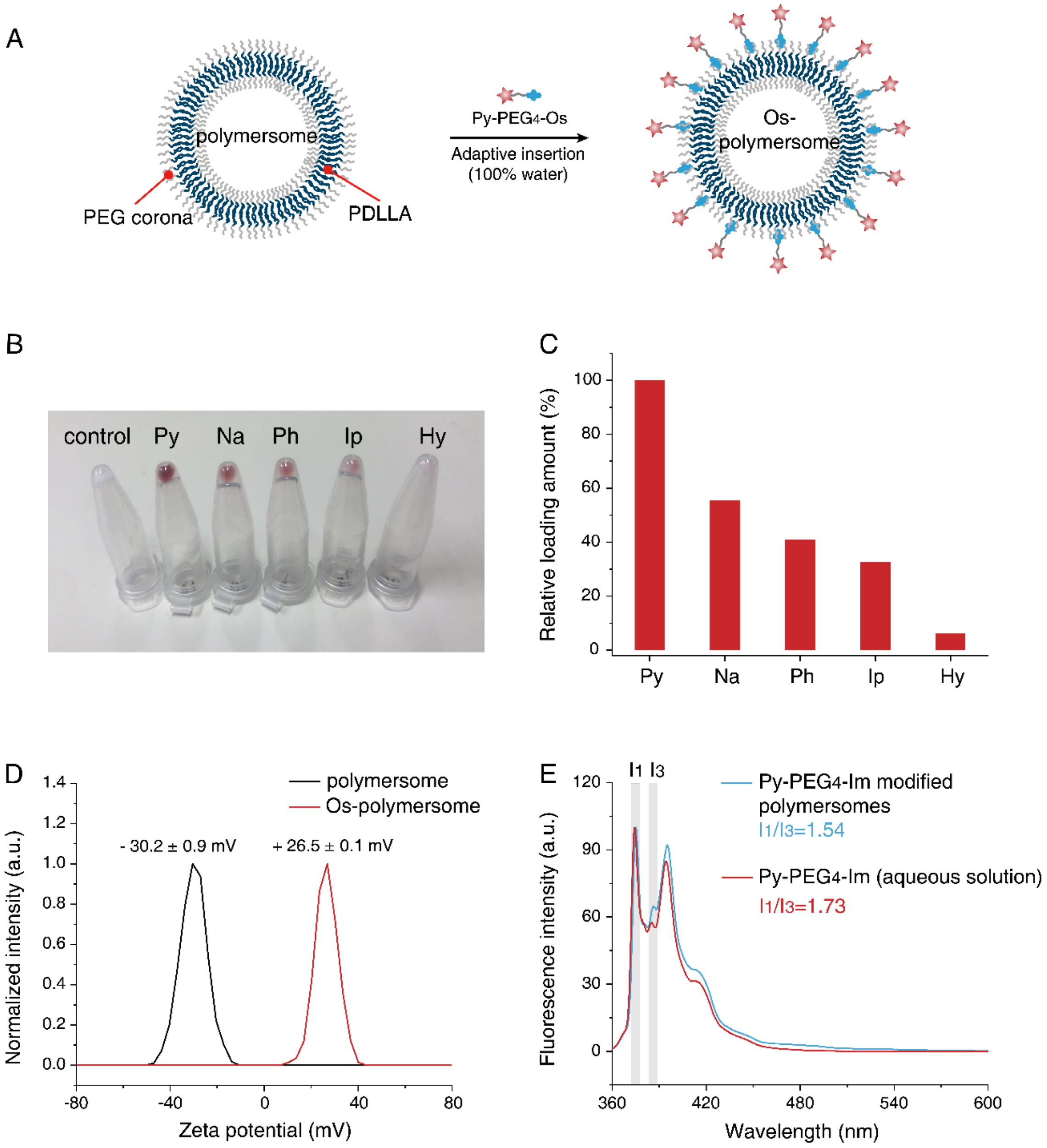

Figure 31. (**A**) Schematic loading of molecular probes onto PEG-*b*-PDLLA polymersomes. (**B**) Optical image of the precipitated PEG-*b*-PDLLA polymersomes loaded with Py-/Na-/Ph-/Ip-/Hy-PEG$_4$-Os. The control was blank PEG-*b*-PDLLA polymersomes. (**C**) The relative loading amount of Py-/Na-/Ph-/Ip-/Hy-PEG$_4$-Os on PEG-*b*-PDLLA polymersomes. Each value corresponds to mean of $n = 2$ replicates. (**D**) Zeta potential of PEG-*b*-PDLLA polymersomes before and after the modification by Py-PEG$_4$-Os. (**E**) Normalized fluorescence of Py-PEG$_4$-Im loaded onto PEG-*b*-PDLLA polymersomes and Py-PEG$_4$-Im dissolved in water.

6.7 The loading of molecular probes onto PEG-modified gold nanoparticles

6.7.1 Synthesis of PEGn-SH

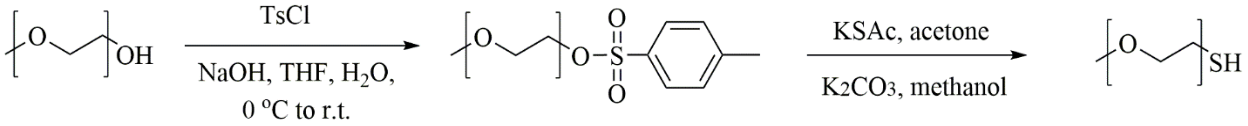

PEGn-SH was synthesized according to a previously published procedure[11]. Here, PEG with different molecular weights was used as the initial materials. PEGn-SH was characterized by ^{1}H NMR, ^{13}C NMR, and mass spectrometer. n was calculated from the integration of ^{1}H NMR.

PEG_{11}-SH

^{1}H NMR (400 MHz, $CDCl_3$) δ [ppm] = 3.52-3.85 (m, 42H, PEG backbone), 3.38 (s, 3H, CH_3O), 2.70 (dt, J = 8.2, 6.4 Hz, 2H, CH_2-CH_2-SH), (1.59, t, J = 8.2 Hz, 1H, -S*H*). ^{13}C NMR (101 MHz, $CDCl_3$) δ [ppm] = 72.91, 71.96, 70.67, 70.64, 70.60, 70.54, 70.27, 59.05, 24.29.

PEG_{26}-SH

^{1}H NMR (400 MHz, $CDCl_3$) δ [ppm] = 3.44-3.85 (m, 103H, PEG backbone), 3.38 (s, 3H, CH_3O), 2.70 (dt, J = 8.2, 6.4 Hz, 2H, CH_2-CH_2-SH), (1.59, t, J = 8.2 Hz, 1H, -S*H*). ^{13}C NMR (101 MHz, $CDCl_3$) δ [ppm] = 72.91, 71.96, 70.60, 70.54, 70.27, 59.05, 24.29.

PEG_{42}-SH

^{1}H NMR (400 MHz, $CDCl_3$) δ [ppm] = 3.42-3.87 (m, 166H, PEG backbone), 3.38 (s, 3H, CH_3O), 2.70 (dt, J = 8.2, 6.4 Hz, 2H, CH_2-CH_2-SH), (1.59, t, J = 8.2 Hz, 1H, -S*H*). ^{13}C NMR (101 MHz, $CDCl_3$) δ [ppm] = 72.91, 71.96, 70.60, 70.27, 59.05, 24.29.

PEG_{111}-SH

^{1}H NMR (400 MHz, $CDCl_3$) δ [ppm] = 3.43-3.89 (m, 444H, PEG backbone), 3.38 (s, 3H, CH_3O), 2.70 (dt, J = 8.2, 6.4 Hz, 2H, CH_2-CH_2-SH), (1.59, t, J = 8.2 Hz, 1H, -S*H*). ^{13}C NMR (101 MHz, $CDCl_3$) δ [ppm] = 70.59.

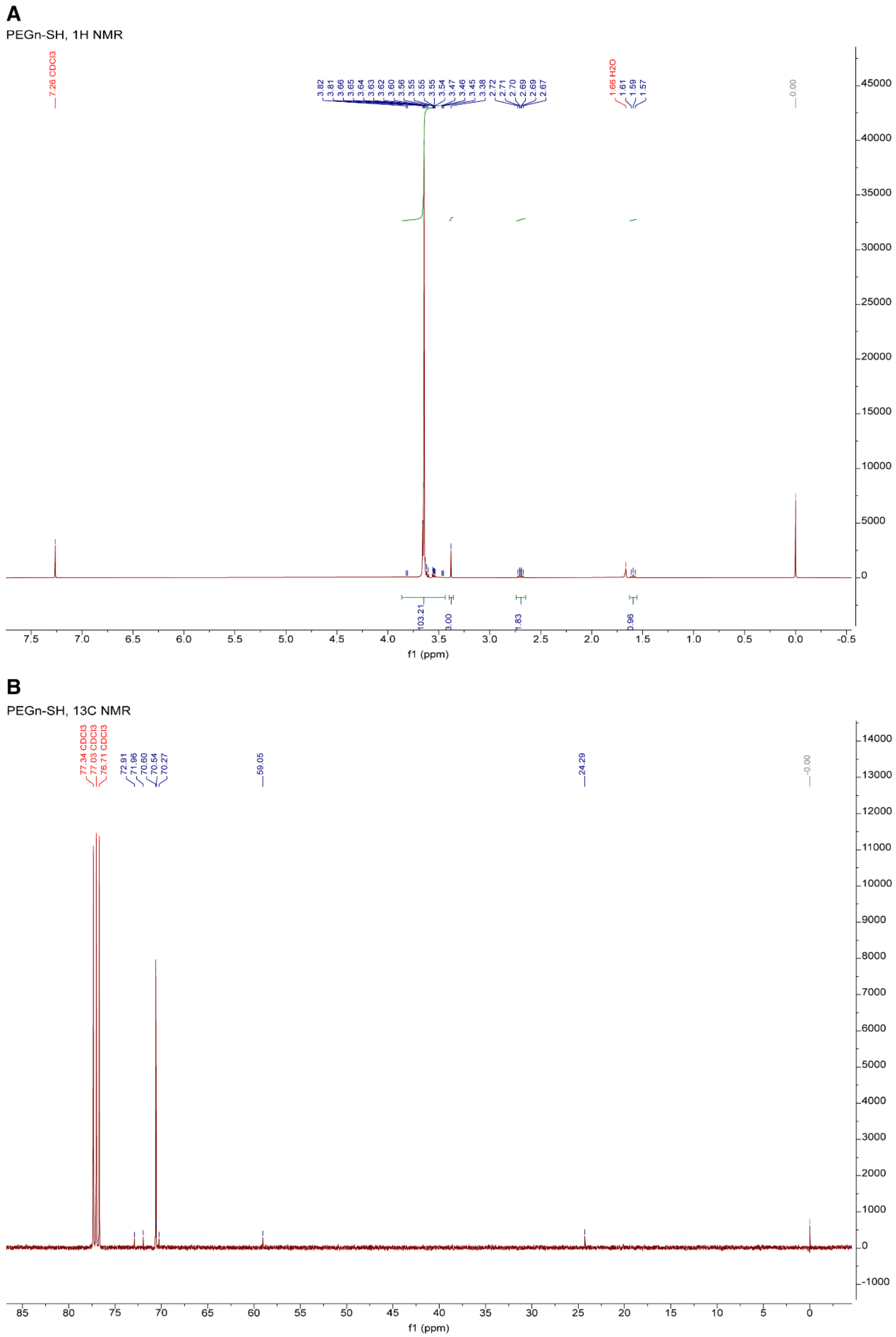

Figure 32a. (**A**) ^{1}H NMR and (**B**) ^{13}C NMR spectra of PEG_{26}-SH in $CDCl_3$.

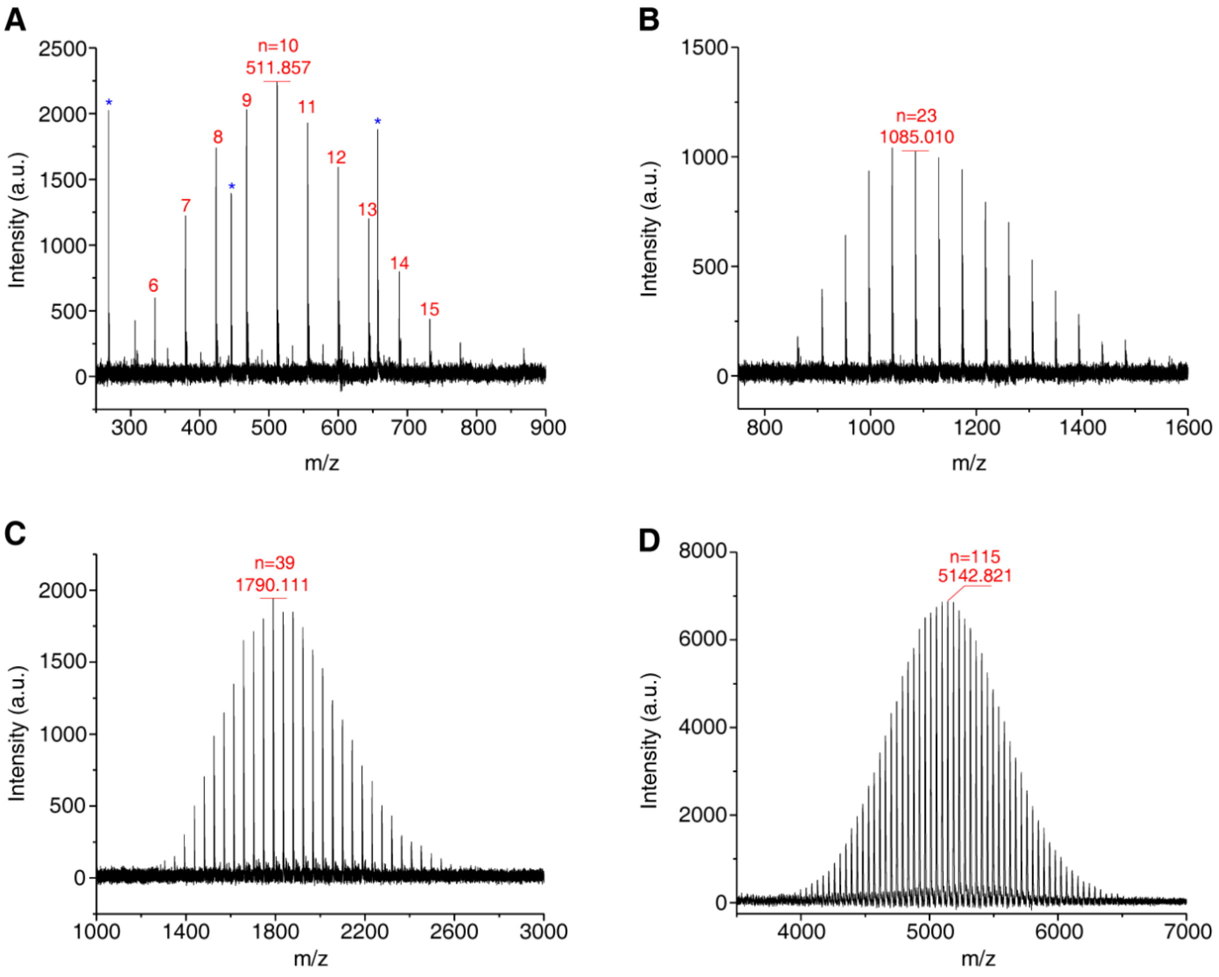

Figure 32b. MALDI-TOF spectra of PEG_n-SH with different molecular weights. The peaks marked by the blue * arise from the matrix of MALDI-TOF.

6.7.2 Preparation of PEG-modified gold nanoparticles

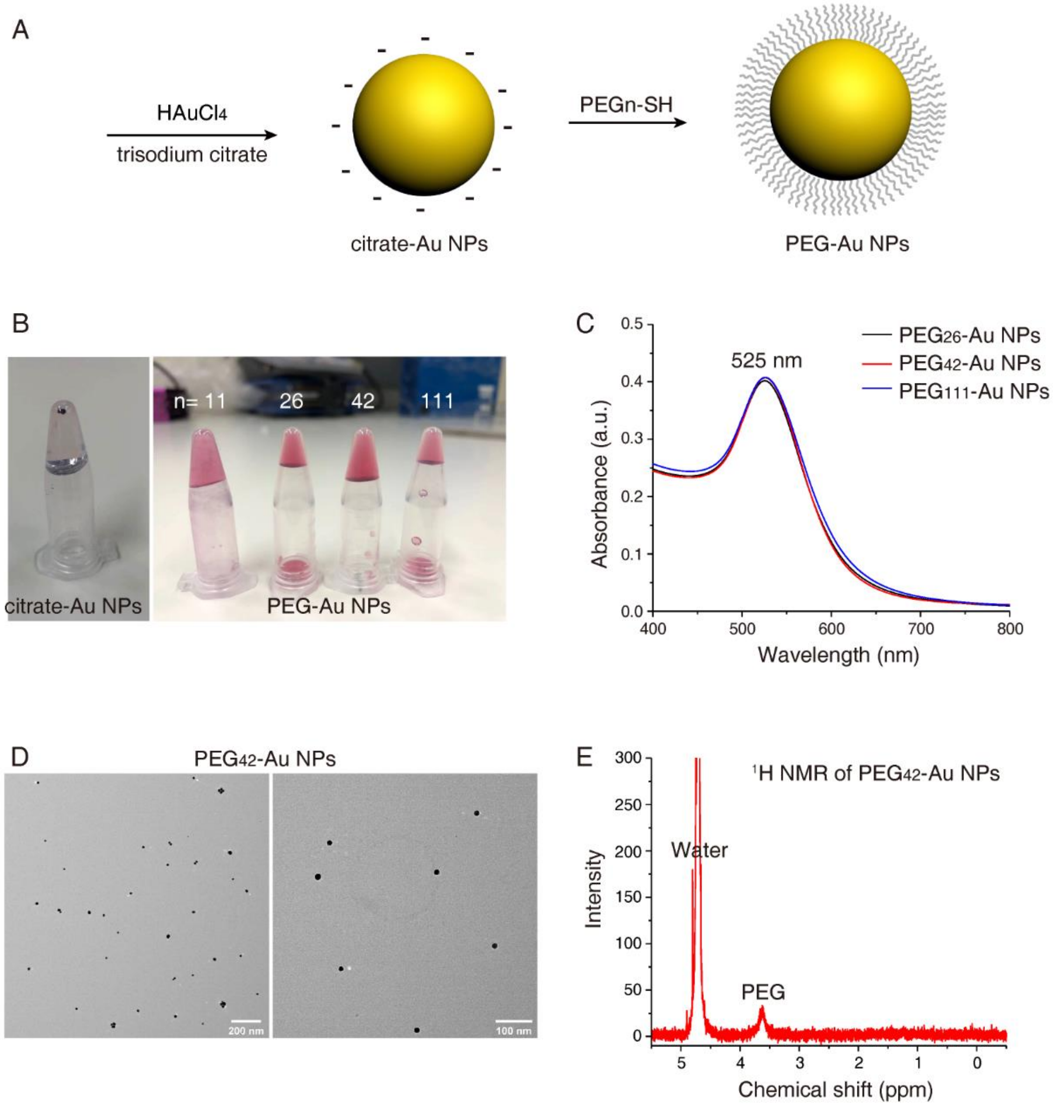

Figure 33. Preparation and characterization of PEG-Au NPs. (**A**) Schematic preparation of PEG-Au NPs. (**B**) Optical images of citrate-Au NPs and PEG-Au NPs after water wash and centrifugation 3 times. (**C**) UV-vis spectra of the aqueous dispersion of PEG_{26}-Au NPs, PEG_{42}-Au NPs, and PEG_{111}-Au NPs. (**D**) TEM images of PEG_{42}-Au NPs. (**E**) ^{1}H NMR spectrum of PEG_{42}-Au NPs.

PEG-modified gold nanoparticles (PEG-Au NPs) were prepared through the well-developed ligand exchange strategy (Supplementary Fig. 33)[12]. Citrate-modified gold nanoparticles (citrate-Au NPs) were first prepared according to the previously reported procedure[13]. The diameter of citrate-Au NPs was determined to be 13.6 ± 2.6 nm by TEM. The concentration of Au-NPs was

calculated to be 11.7 nM based on the previously reported method[14]. To prepare PEG-Au NPs, 200 uL PEG-SH solution (2 mM) was added to 1 mL citrate-Au NPs. After incubating at room temperature for 24 h, PEG-Au NPs were collected by centrifugation and water wash. As shown in Supplementary Fig. 33, citrate-Au NPs aggregated after being washed with water two times. After ligand exchange, PEG-Au NPs kept stable during the centrifugation and water wash, except PEG_{11}-Au NPs, which stuck to the Eppendorf tube. The aggregation of PEG_{11}-Au NPs should be attributed to the short PEG ligand, which failed in stabilizing Au NPs. The well-dispersed PEG-Au NPs were characterized by a UV-vis spectrometer. As shown in Supplementary Fig. 33, a narrow SPR peak was detected, confirming the homogenous dispersion of $PEG_{26/42/111}$-Au NPs[13]. TEM further confirmed the successful preparation of PEG_{42}-Au NPs (Supplementary Fig. 33). PEG on the surface of Au NPs was detected by ^{1}H NMR (Supplementary Fig. 33)[15]. These characterizations demonstrated the successful preparation of PEG-Au NPs.

6.7.3 The loading of molecular probes onto PEG-modified gold nanoparticles

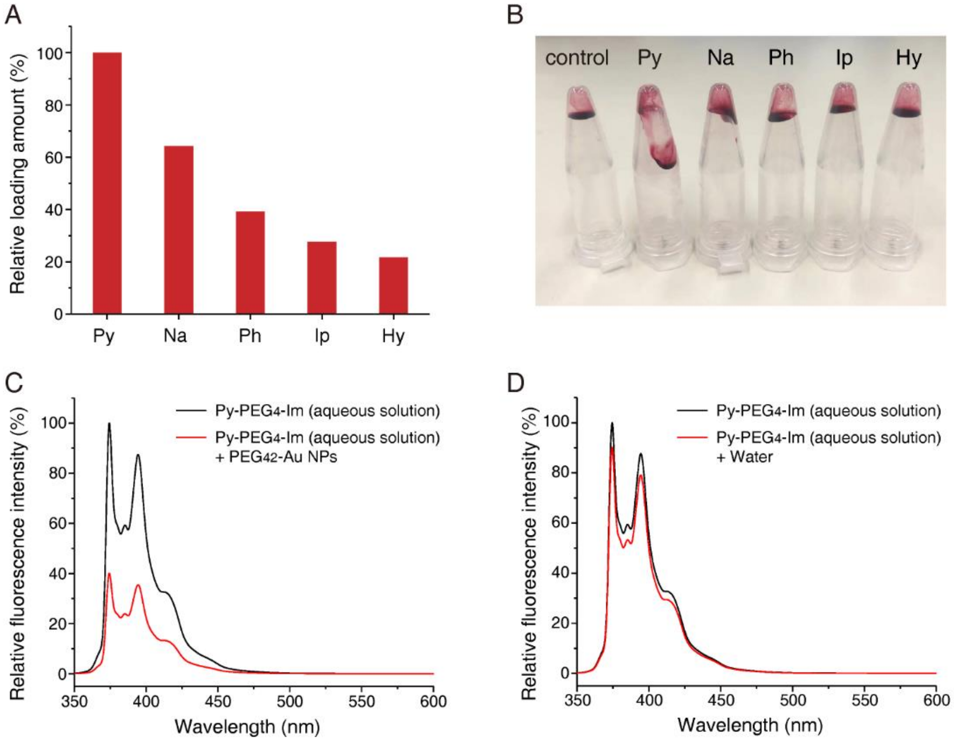

Figure 34. The loading of molecular probes onto PEG-Au NPs. (**A**) The relative loading amount of Py-/Na-/Ph-/Ip-/Hy-PEG_4-Os onto PEG_{42}-Au NPs. (**B**) Optical image of the precipitated PEG_{42}-Au NPs loaded with Py-/Na-/Ph-/Ip-/Hy-PEG_4-Os. The control was the blank PEG_{42}-Au NPs.

Relative fluorescence of Py-PEG_4-Im before and after the addition of (**C**) PEG_{42}-Au-NPs or (**D**) the same volume of water.

PEG_{42}-Au NPs with similar PEG in length as PEG-*b*-PS polymersomes, were used as the host to load molecular probes. In the loading solution, the concentration of molecular probes was 10 μM, and the concentration of PEG_{42}-Au NPs was ~4.9 nM. After incubation for 10 min, the absorbance of the supernatant without PEG-Au NPs was measured. As shown in Supplementary Fig. 34, the molecular probes were successfully loaded onto PEG-Au NPs. In analogous to PEG-*b*-PS polymersomes, the loading amount got increased with the anchor hydrophobicity. This again validated the interaction between the hydrophobic anchor and PEG host. However, unlike polymersomes, the loading of molecular probes onto PEG_{42}-Au NPs can not be visualized due to the purple color of PEG_{42}-Au NPs (Supplementary Fig. 34).

The loading of the molecular probe onto PEG_{42}-Au NPs was further studied by a fluorescence spectrometer. As reported previously, Au NPs can quench the fluorescence of Py by energy transfer, when they are physically close to each other[16]. Here, the fluorescence of Py-PEG_4-Im was monitored when PEG-Au NPs were added. As shown in Supplementary Fig. 34, the fluorescence intensity of Py got significantly decreased after the addition of PEG-Au NPs (~39 nM, 100 uL). However, when the same volume of water was added, the fluorescence intensity of Py was only slightly decreased (Supplementary Fig. 34). The quenching of Py by PEG_{42}-Au NPs was attributed to the loading of Py-PEG_4-Im onto PEG_{42}-Au NPs[16]. The above results confirmed the successful loading of molecular probes onto the PEG monolayer of PEG_{42}-Au NPs.

7. Conformation of molecular probes loaded onto PEG corona

7.1 The loading of different molecular probes onto PEG-*b*-PS polymersomes

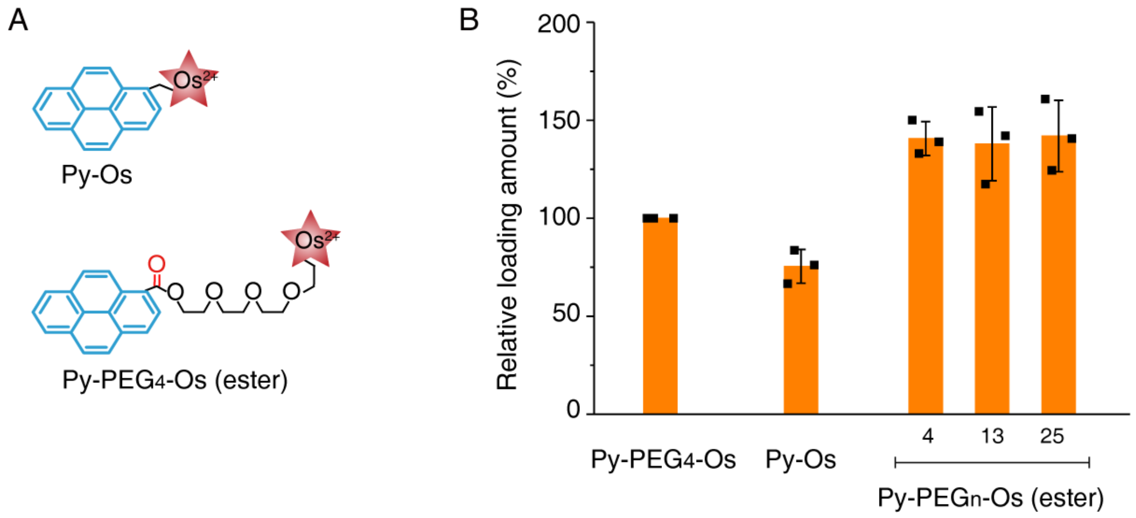

Figure 35. (**A**) Structure of Py-Os and Py-PEG$_4$-Os (ester). (**B**) The relative loading amount of Py-PEG$_4$-Os, Py-Os, and Py-PEG$_n$-Os (ester). Molecular probes were loaded onto PEG-*b*-PS polymersomes in water. Data are presented as the mean ± s.d. of n = 3 independent experiments.

7.2 Surface accessibility of the loaded Os

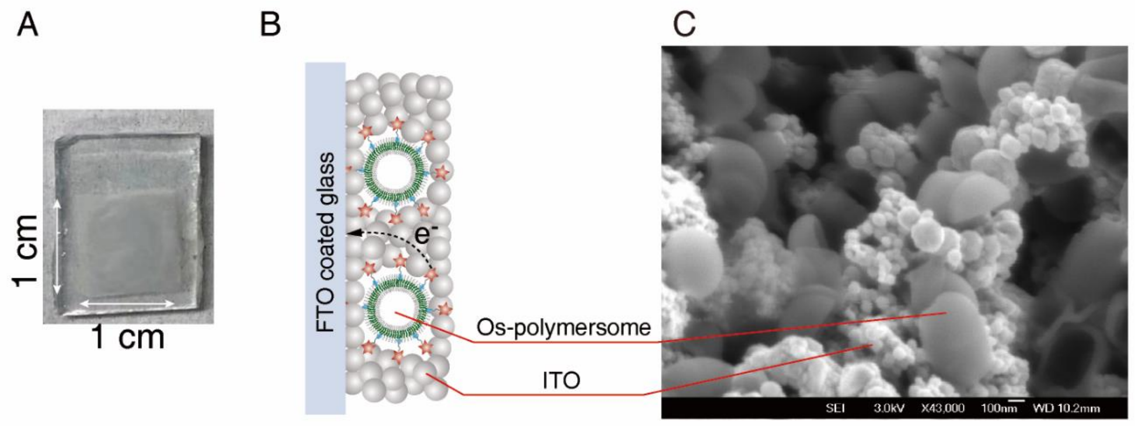

Figure 36. (**A**) The optical image, (**B**) schematic illustration, and (**C**) SEM image of the working electrode. The working electrode was prepared by drop-casting the mixture of ITO nanoparticles and polymersomes modified by Py-PEG$_4$-Os (ester) (Os-polymersomes) onto FTO-coated glass.

8. Adaptive loading of molecular probes

8.1 The adsorption of PSS onto PEG-*b*-PS polymersomes

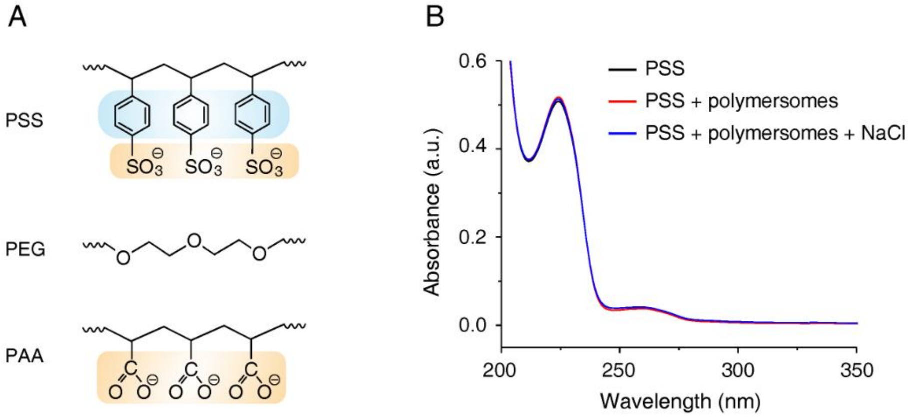

Figure 37. (**A**) Chemical structure of PSS, PEG, and PAA. (**B**) The absorbance of supernatant for the mixture of PSS and polymersomes. The concentration of PSS was 60 μM. The concentration of NaCl (if included) was 10 mM. PSS was not adsorbed onto PEG-*b*-PS polymersomes in both water and 10 mM NaCl.

8.2 Stability of PEG-*b*-PS polymersomes

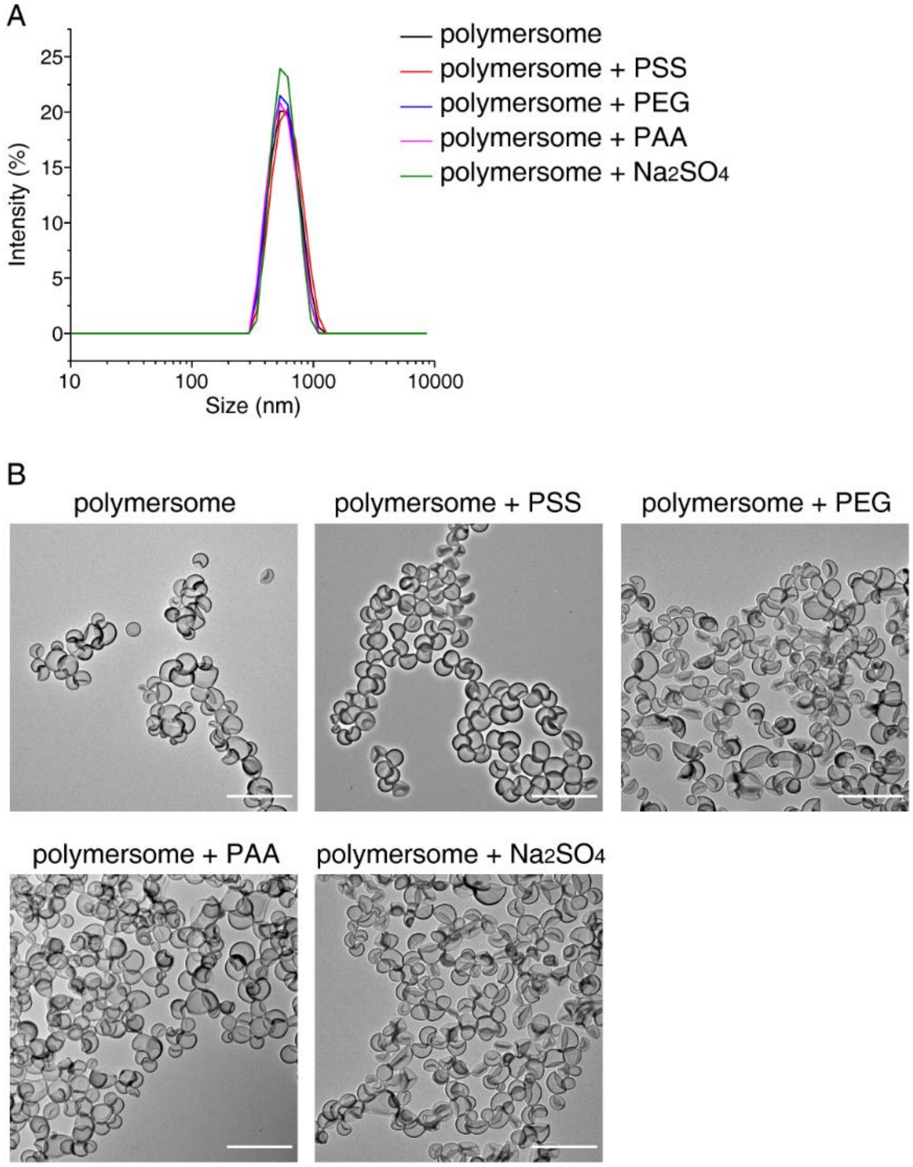

Figure 38. (**A**) DLS intensity size distribution of PEG-*b*-PS polymersomes incubated with 10 mM PSS, PEG, PAA, and Na_2SO_4 for 10 min. (**B**) TEM images of the corresponding polymersomes. The scale bar is 2 μm.

9. References

1. Tu, Y. et al. Self-propelled supramolecular nanomotors with temperature-responsive speed regulation. *Nat. Chem.* **9**, 480-486 (2017).
2. Habermüller, K., Ramanavicius, A., Laurinavicius, V. & Schuhmann, W. An oxygen-insensitive reagentless glucose biosensor based on osmium-complex modified polypyrrole. *Electroanalysis*, **12**, 1383-1389 (2000).
3. Tsai, C. Y., Chung, C. H. & Hong, J. L. Pyrene-terminated, amphiphilic polypeptide and its hydrogen bonded interpolymer complex as delivery systems of doxorubicin. *ACS Omega* **3**, 4423-4432 (2018).
4. Espinoza, E. M. et al. How do amides affect the electronic properties of pyrene? *ACS Omega* **3**, 12857-12867 (2018).
5. Sokol, K. P. et al. Bias-free photoelectrochemical water splitting with photosystem II on a dye-sensitized photoanode wired to hydrogenase. *Nat. Energy* **3**, 944-951 (2018).
6. Yan, L., Higbee, E., Tsourkas, A., & Cheng, Z. A simple method for the synthesis of porous polymeric vesicles and their application as MR contrast agents. *J. Mat. Chem. B* **3**, 9277-9284 (2015).
7. Keller, S., Teora, S. P., Hu, G. X., Nijemeisland, M. & Wilson, D. A. High-throughput design of biocompatible enzyme-based hydrogel microparticles with autonomous movement. *Angew. Chem. Int. Ed.* **57**, 9814-9817 (2018).
8. Yamamoto, Y., Okada, D., Kushida, S., Ngara, Z. S., & Oki, O. Fabrication of polymer microspheres for optical resonator and laser applications. *J. Vis. Exp.* **124**, e55934 (2017).
9. Xu, L. & Yang, X. Molecular dynamics simulation of adsorption of pyrene-polyethylene glycol onto graphene. *J. Colloid Interface Sci.* **418**, 66-73 (2014).
10. Toebes, B. J., Cao, F. & Wilson, D. A. Spatial control over catalyst positioning on biodegradable polymeric nanomotors. *Nat. Commun.* **10**, 1-6 (2019).
11. Gobbo, P. & Workentin, M. S. Improved methodology for the preparation of water-soluble maleimide-functionalized small gold nanoparticles. *Langmuir* **28**, 12357-12363 (2012).
12. Xia, X. et al. Quantifying the coverage density of poly (ethylene glycol) chains on the surface of gold nanostructures. *ACS Nano* **6**, 512-522 (2012).
13. D'Agata, R., Palladino, P. & Spoto, G. Streptavidin-coated gold nanoparticles: critical role of oligonucleotides on stability and fractal aggregation. *Beilstein J. Nanotechnol.* **8**, 1-11 (2017).

14. Xu, X. H. N., Huang, S., Brownlow, W., Salaita, K., & Jeffers, R. B. Size and temperature dependence of surface plasmon absorption of gold nanoparticles induced by tris(2,2‘-bipyridine) ruthenium(II). *J. Phys. Chem. B* **108**, 15543-15551 (2004).
15. Lu, J. et al. A non-sacrificial method for the quantification of poly(ethylene glycol) grafting density on gold nanoparticles for applications in nanomedicine. *Chem. Sci.* **10**, 2067-2074 (2019).
16. Xu, J. P. et al. Highly soluble PEGylated pyrene-gold nanoparticles dyads for sensitive turn-on fluorescent detection of biothiols. *Analyst* **135**, 2323-2327 (2010).
